# DNA traces the origin of honey by identifying plants, bacteria and fungi

**DOI:** 10.1038/s41598-021-84174-0

**Published:** 2021-02-26

**Authors:** Helena Wirta, Nerea Abrego, Kirsten Miller, Tomas Roslin, Eero Vesterinen

**Affiliations:** 1 Faculty of Agriculture and Forestry, University of Helsinki, P.O. Box 27, 00014 Helsinki, Finland; 2 Department of Biological and Environmental Science, University of Jyväskylä, P.O. Box 35, 40014 Jyväskylä, Finland; 3 Department of Ecology, Swedish University of Agricultural Sciences, P.O. Box 7044, 750 07 Uppsala, Sweden; 4 School of Natural and Environmental Sciences, Newcastle University, Newcastle-upon-Tyne, NE1 7RU UK; 5 Department of Biology, University of Turku, Turku, Finland

**Keywords:** Environmental microbiology, Biological techniques, Genomic analysis, Agroecology

## Abstract

The regional origin of a food product commonly affects its value. To this, DNA-based identification of tissue remains could offer fine resolution. For honey, this would allow the usage of not only pollen but all plant tissue, and also that of microbes in the product, for discerning the origin. Here we examined how plant, bacterial and fungal taxa identified by DNA metabarcoding and metagenomics differentiate between honey samples from three neighbouring countries. To establish how the taxonomic contents of honey reflect the country of origin, we used joint species distribution modelling. At the lowest taxonomic level by metabarcoding, with operational taxonomic units, the country of origin explained the majority of variation in the data (70–79%), with plant and fungal gene regions providing the clearest distinction between countries. At the taxonomic level of genera, plants provided the most separation between countries with both metabarcoding and metagenomics. The DNA-based methods distinguish the countries more than the morphological pollen identification and the removal of pollen has only a minor effect on taxonomic recovery by DNA. As we find good resolution among honeys from regions with similar biota, DNA-based methods hold great promise for resolving honey origins among more different regions.

## Introduction

Flowers of different plants offer divergent nectar, resulting in honeys that vary in their taste, look and smell, as well as in their texture (as an example in whether the honey stays liquid or crystallizes fast^1^). Likewise, health benefits vary among the honey collected from different flowers^2–4^. On top of these properties, honey, along with many other food products, is notably valued to its regional origin^5,6^. Much effort has been invested in methods for determining the exact origin of honey^5,6^ since, as with any commercial product, adulteration does occur. While the addition of inexpensive sugars or sugar syrups to honey to increase the volume, and heating or filtering honey to make honey stay liquid longer, are among the most common types of honey adulteration, so is the adulteration of honey origin^6^. This may occur as incorrect indication of origin, or by adding honey or pollen from another region than indicated^6–8^. There is thus an eminent need for accurate methods capable of identifying the regional origin of honey, on top of the need to detect the other sources of adulteration.

The traditional and most commonly used method for defining the regional origin of honey is to identify the plants from which bees have collected the nectar based on the morphological characters of pollen found in honey (melissopalynology^9^). Based on the plants identified and the distributional data of the plants, the regional origin of the honey is then assessed. This is a laborious and expertise-demanding method^5^. Also, using pollen morphology often limits the plant identification to the family level^1,9^, thereby constraining the resolution of the regional origin. Furthermore, honey can be filtered to make it stay liquid longer^7^. This practice removes the pollen, i.e. the very particles used for identification. Organoleptic properties and several chemical markers of honey are also used to define the origin of honey, but these markers are susceptible to details of the beekeeping techniques and honey processing. Thus they are most commonly used as complements to traditional analyses of origin, which are generally still based on the morphological identification of pollen^6^.

The last few years have seen the advent of DNA-based methods for identifying the floral sources in honey^10–13^. These methods have primarily targeted the pollen content of honey, as it is considered to provide key information on honey origin. Pollen is collected by the bees as a nutrient, but it is also carried inadvertently to the hive in the hair of bees who have visited flowers for nectar. Yet, plants do not offer pollen in equal amounts, and some plants offer nectar without any detectable amount of pollen entering the honey^5^. Thus, identifying plants from non-pollen parts could be a vital complement to pollen-based honey identification, as recently proposed^14^.

To improve the resolution of origin, we could use information also from other taxonomic groups found in honey, on top of plants. The microbial contents of honey could be identified by DNA based methods^15^. As the chemical properties of honey (e.g. low water activity, low pH, hydrogen peroxide) inhibit microbial growth^16^, dormant forms of microbes are typically found in honey, e.g. bacterial spores and yeasts. These particles will emanate from the flowers visited^17–20^, from the surroundings of the hive (e.g. from the soil and water^21^) and from the hive and the bee itself^22,23^. Thus, they may provide added clues to honey origins.

Establishing the origin of honey based on pollen will be particularly hard for samples originating from regions close and similar to each other, as characterised by few or no higher plant taxa unique to each region. To refine the distinction of honey by origin, we therefore ask:

1. Can honey samples from three neighbouring countries with similar biotas (Finland, Sweden and Estonia) be separated by DNA-based methods using plant, bacterial and/ or fungal taxa found in honey?
2. Does the identification method (morphological identification of pollen, DNA metabarcoding or metagenomics) affect the distinction among origins?
3. Does the removal of pollen and other larger particles by filtering affect which taxa can be detected in honey by DNA?

## Results

### Summary of the methods used

**Figure 1:**
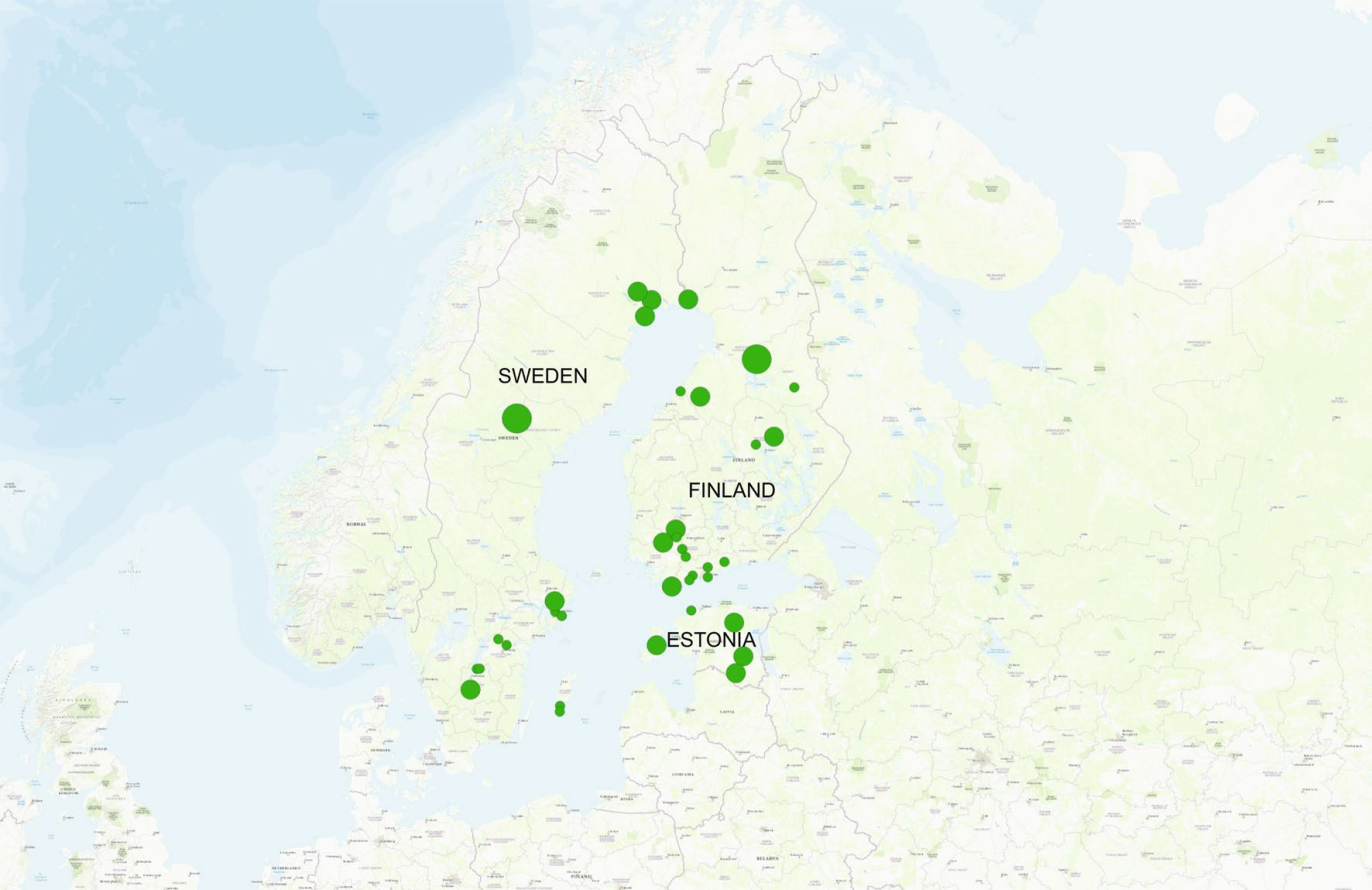
Map of the three northern Europe countries (Estonia, Finland, Sweden) from which honey samples were obtained. The size of the circles indicates the number of beekeepers the samples originate from, smallest circles indicating the honey samples originate from a single beekeeper and large circles from multiple beekeepers. The map was created with the program QGIS, version 3.10.4 (https://qgis.org/).

We obtained in total 46 honey samples from Finland, Sweden and Estonia (Fig. 1, Supplementary Table S1). First, six subsamples of 10 g of each honey sample were diluted in water, and two of the subsamples were filtered through a syringe to remove pollen and other larger particles. After centrifugation, the pellets from two subsamples from either non-filtered or filtered were combined, generating two non-filtered and one filtered samples per each original honey sample. DNA was extracted from all these samples. Two methods of DNA-based identification were used: DNA metabarcoding targeted at plants, bacteria and fungi, and DNA metagenomics. The filtered sample and one non-filtered sample were examined with DNA metabarcoding, and the other non-filtered sample was examined with metagenomics. On top of the DNA based identification, the pollen contents of each original honey sample were identified by an accredited commercial laboratory.

To examine the differentiation of the taxonomic composition of the honey samples among the three countries, we examined the data for the taxonomic groups of plants, bacteria and fungi, at the taxonomic levels of operational taxonomic units (OTU), genus and family, from both metabarcoding and metagenomics. First, to look at the clustering of the samples per country, we used nonmetric multidimensional scaling (NMDS)^24^. Second, to assess how much the countries share the taxa, we constructed Euler diagrams^25^. Third, we quantified how much of the variation in the composition of taxa among samples was explained by the country by fitting joint distribution models^26^ to each of the datasets. To assess how the removal of pollen and other larger particles affects the information available from honey with DNA based identification, we examined the filtered and non-filtered samples with NMDS^25^ plots and Euler diagrams^24^. For detailed description of the methods and analyses, see the Methods section.

### DNA contents of honey

From the honey samples from Finland, Sweden and Estonia, we identified plants with the gene regions ITS2, *rbc*L*a* and *trn*L, bacteria with the gene region 16S rRNA with two primer pairs (here called 16Sa and 16Sb) and fungi with the gene region ITS by metabarcoding (Supplementary Table S2). For these the number of reads obtained from sequencing varied among the gene regions, from one million (for *trn*L) to nearly seven million (for 16S rRNA with the primer pair 16Sa) paired-end reads. Of these, 70–93% were mapped to OTUs after merging and filtering (Supplementary Table S3). OTU mapping was followed by further filtering, yielding the final number of OTUs used for analyses which were then assigned to taxa (Table 1). For metagenomics, an initial total of 125 million reads were obtained for the samples, out of which eighty million reads (64%) could be assigned to taxa.

**Table 1:** Number of taxa identified by different methods. For metabarcoding of plants, we used gene regions ITS2, *rbc*L*a* and *trn*L, for metabarcoding of bacteria we used the 16S rRNA gene region with two primer sets (16Sa and 16Sb), and for metabarcoding of fungi we used ITS. Note that for the number of OTUs, the OTUs from both non-filtered and filtered samples are counted.

Taxonomic group	Taxonomic level	Morphology	ITS2	*rbc*L*a*	*trn*L	Metagenomics
Plants	OTUs		409	621	163	
	Species	4	125	55	7	
	Genera	37	163	145	42	399
	Families	32	59	90	49	145

				16Sa	16Sb	Metagenomics
Bacteria	OTUs			1050	867	
	Genera			359	227	1093
	Families			167	151	334

					ITS	Metagenomics
Fungi	OTUs				826	
	Species				101	
	Genera				118	201
	Families				89	117

Based on the taxonomic assignments of the metabarcoding and the metagenomics data, as well as morphological identification of pollen, a wide variety of organisms were detected from the honey samples (Table 1).

For the metabarcoding data on plants, somewhat different taxa were detected for the different gene regions (for summary numbers, see Table 1). With metagenomics, we identified 399 genera and 145 families of plants. For the metagenomics, as well as for the comparison among the methods, we consider genera and families (see Supplementary text S1). Metabarcoding of bacteria revealed far lower numbers of genera and families than were found by metagenomics, whereas for fungi, we found similar number of families with both metabarcoding and metagenomics, but clearly more genera with metagenomics (Table 1).

### Differentiation among countries by OTUs of different taxonomic groups

**Figure 2:**
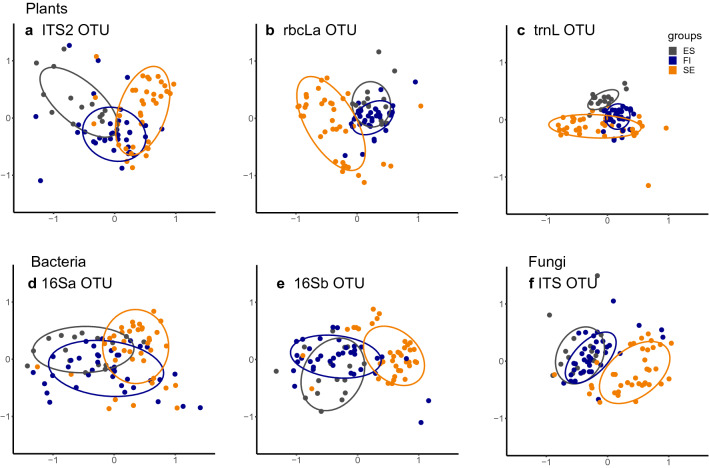
Nonmetric multidimensional scaling (NMDS) plots for plant, bacterial and fungal OTU community similarities among samples originating from the three different countries. The ellipses confine 75% of the data points. Stress values for the different gene region analyses are given in Supplementary Table S4. The panels show the data from different gene regions: plants: (**a**) ITS2, (**b**) *rbc*L*a*, (**c**) *trn*L, bacteria: (**d**) 16Sa, (**e**) 16Sb, and fungi: (**f**) ITS. Samples from Estonia (ES) are shown in gray, from Finland (FI) in blue and from Sweden (SE) in light orange.

In terms of the similarity of the community composition between countries, the nonmetric multidimensional scaling (NMDS) analyses applied at the OTU level revealed large variation among gene regions (Fig. 2, Supplementary Table S4 for NMDS stress values). The plant gene region, *trn*L (Fig. 2c) showed the most distinct clusters between countries. For plant *rbc*L*a* (Fig. 2b), bacterial 16Sb (Fig. 2e) and fungal ITS2 (Fig. 2f) the samples from Estonia and Finland largely overlapped, but were clearly separated from the Swedish samples.

Likewise, Euler diagrams revealed large variation among gene regions in the total number of OTUs (cf. Table 1), as well as which ones were unique to each country and which were shared between countries (Supplementary Fig. S1). For all but the bacterial gene regions, the samples from Sweden showed the highest number of OTUs, while for the bacterial gene regions, more OTUs were detected in the samples from Finland. The gene regions ITS2 for plants and ITS for fungi (Supplementary Fig. S1) showed the smallest proportion of OTUs shared among all three countries.

The joint species distribution models^26^ fitted to the different OTU level datasets explained 4.5 to 14% of the total variance in species composition, as measured by Tjur’s R^2^^27^, and 72.7 to 81.3% as measured by the Area Under the Curve (AUC)^28^ (Supplementary Table S5). The overall proportion of the data explained was the smallest for the bacterial OTU datasets, both 16Sa and 16Sb, while the largest proportion explained by the model was for the plant *trn*L. For all gene regions, the large majority of the explained variation (69.8 to 78.8%) was assigned to the country of origin of the honey samples, while 4.4 to 15.8% was assigned to the treatment (non-filtered or filtered) and 8.8 to 18.9% to the read count (Supplementary Table S5). In terms of the proportion of the total variation explained assigned to the country of origin (measured by as Tjur’s R^2^), the plant gene region *trn*L separated the samples from different countries most clearly, with plant *rbc*L*a* and fungal ITS also showing a clear distinction (Fig. 2). This analysis supported the same difference among gene regions and the separation of samples from different countries as revealed by the NMDS analysis (Fig. 2). While the proportion of data remaining unexplained may appear large on the basis of (1 − Tjur R^2^) values, it is noteworthy that Tjur R^2^ values small by R^2^ standards do not imply a bad model fit^27^. In fact, all of the models yielded AUC values larger than 0.70 (and in some cases 0.90), which is considered good (excellent) model accuracy^29^.

### Differentiation among countries at different taxonomic levels, with different methods

The total number of taxa identified for the different taxonomic groups varied greatly among methods (Table 1). Only 37 plant genera could be detected by morphological identification, whereas metagenomics yielded over ten times more (Table 1).

**Figure 3:**
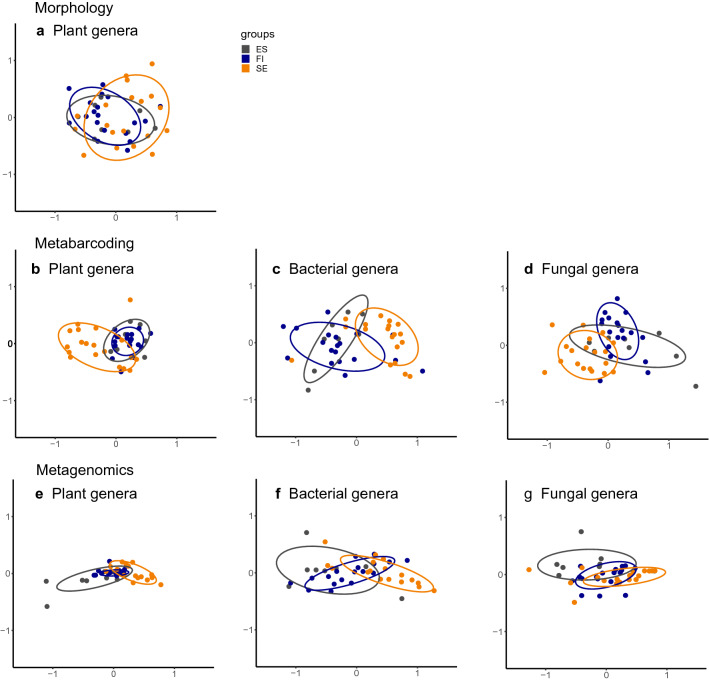
Nonmetric multidimensional scaling (NMDS) plots for plant, bacterial and fungal genera composition similarities among samples originating from the three different countries. The ellipses confine 75% of the data points. Stress values for the different gene region analyses are given in Supplementary Table S4. The panels show the data from different methods morphology (**a**), metabarcoding (**b**–**d**) and metagenomics (**e**–**g**), for different taxonomic groups: (**a**), (**b**) and (**e**) plants, (**c**) and (**f**) bacteria and (**d**) and (**g**) fungi. The samples from Estonia (ES) are shown in grey, from Finland (FI) in blue and from Sweden (SE) in light orange.

The NMDS analysis applied to the plant genera shows the smallest differences between the three countries when pollen was identified by morphology (Fig. 3a), whereas plant data from metabarcoding and metagenomics showed less overlapping clusters (Fig. 3b,e). For plants, the composition of genera in samples from Finland and Estonia were most similar by both metabarcoding and metagenomics (Fig. 3b,e). Also, for bacteria, the composition of genera from Finland and Estonia overlapped with each other to a greater extent than with the samples from Sweden—a pattern suggested by both metabarcoding and metagenomics (Fig. 3c,f). For fungi detected by metabarcoding, taxonomic assignment to genera reveal the most distinct clustering of samples per country, and this pattern was repeated among all the taxonomic groups and all methods (Fig. 3d). For fungi detected by metagenomics, the composition of genera show more overlap among samples from the different countries (Fig. 3g). Turning to family-level assignments, the overlap of samples from different countries was nearly identical to those based on genus-level taxonomic assignment. The only difference here was that when pollen is identified based on morphology, then the samples from Estonia and Finland were totally embedded within those from Sweden (Supplementary Fig. S2).

**Figure 4:**
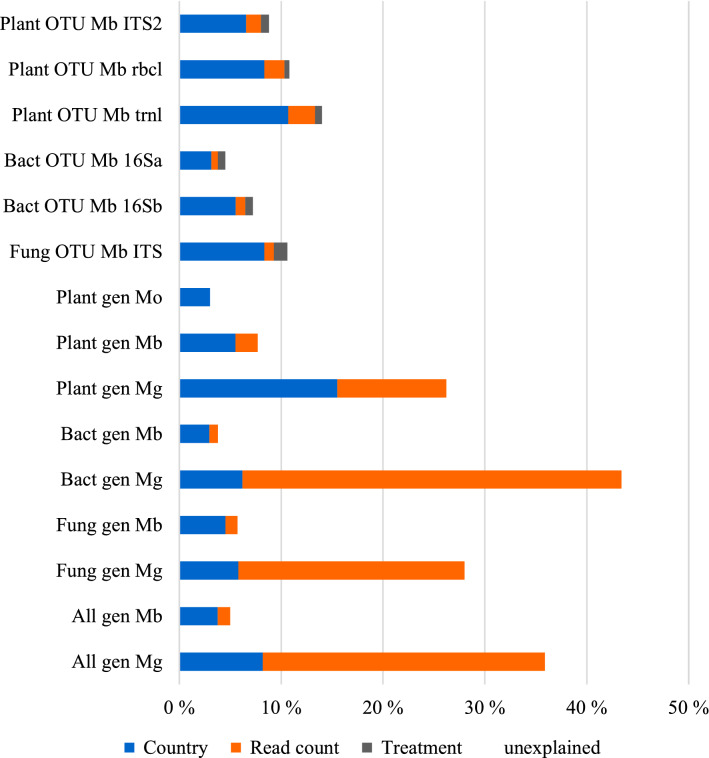
Variation explained by the joint species distribution models fitted to each of the data sets. The total amount of variance explained is quantified by Tjur R^2^, which is shown as the length of the bars. The colors indicate the proportion of variance explained by each of the variables included in the models: country, read count and treatment (filtering, applied only to part of the datasets). The taxonomic groups are plants (Plant), bacteria, (Bact), fungi (Fung) and all (All), the taxonomic levels are OTUs (OTU) and genera, and the methods metabarcoding (Mb), metagenomics (Mg) and morphological identification of pollen (Mo). For metabarcoding the different gene regions are examined at the OTU level separately, and the gene regions are given at the end of the dataset name.

The joint species distribution models fitted to the genus datasets explained 2.8 to 43.4% of the variance in the taxonomic composition of the samples, based on Tjur’s R^2^ (Supplementary Table S5, Fig. 4). Over all the species identification methods, morphological identification of plant genera generated the dataset that explained least variation in the composition of taxa. The models fitted to the metabarcoding datasets explained less variation in the composition of taxa (3.8–7.7%) than the models fitted to the metagenomics datasets (26.3–43.4%; Supplementary Table S5). This was largely due to the sequencing depth having a larger effect on the composition of taxa of the datasets generated with metagenomics, for which most of the explained variation was assigned to read count (40.8–79.3%). In fact, in absolute terms, the country of origin explained 71.6–79.4% for all taxonomic groups in the metabarcoding datasets and 14.3–59.2% with metagenomics datasets (Fig. 4). The genus and family datasets showed very similar patterns overall, in explained variation and the proportions of variation explained by the explanatory variables (Supplementary Table S5). The metagenomics plant genus dataset separates the samples from different countries more clearly than other datasets (15.5% of all variation) with the only exception of plant families identified by metagenomics (15.6%, others 2.8–10.7%; Supplementary Table S5 and Fig. 4). The data separating the samples from different countries second best were the OTU level identifications of plants by the gene region *trn*L (10.7%), followed by fungal ITS (8.4%) and plant *rbc*L*a* (8.3%; Supplementary Table S5, Fig. 4).

**Figure 5:**
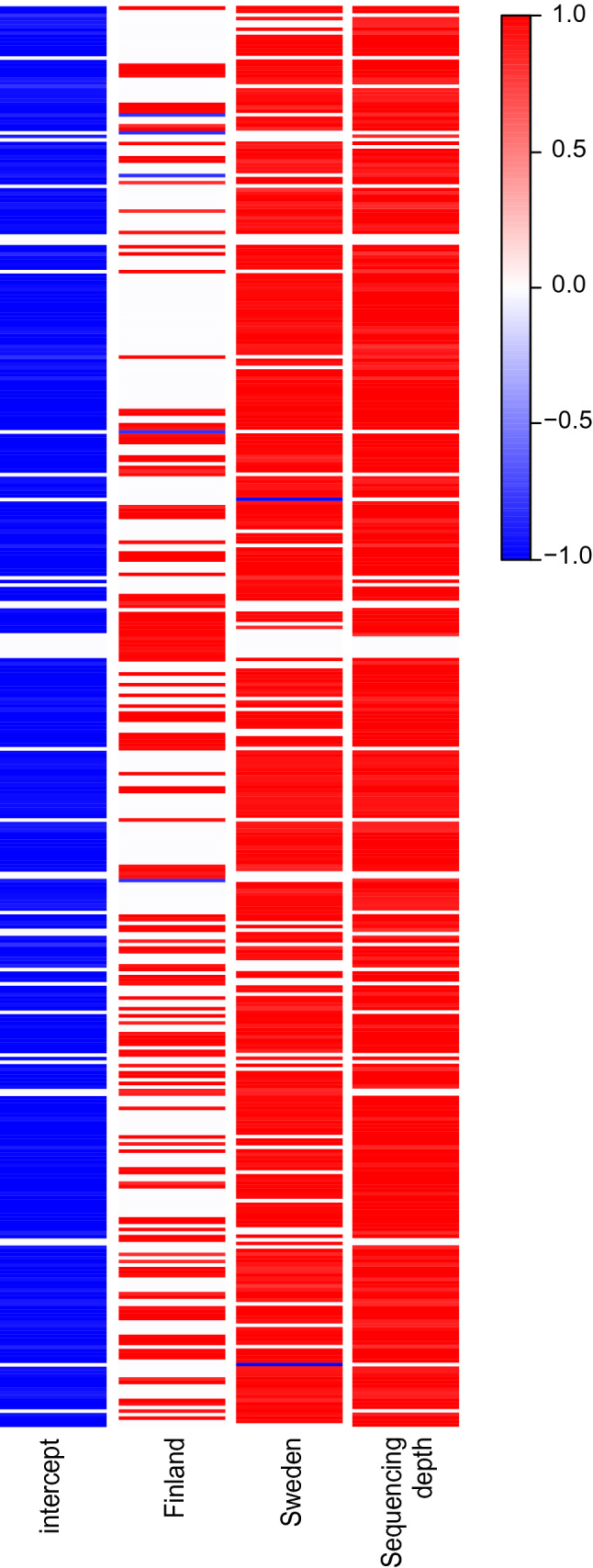
The effect of the covariates of the joint species distribution model, fitted to the plant genera by metagenomics, to each genus, shown by the beta values. Positive beta values showing the genera that respond positively to a covariate, are given in red, and negative values, showing the genera responding negatively, with blue. The strength of the posterior support to the beta values is indicated by the darkness of the colour, shown in the bar. The intercept is country Estonia, and the covariates are the two other countries and the sequencing depth (read count). The plant genera in the plot are listed in Supplementary Table S6, showing the beta values with high (> 95%) posterior support.

Considering joint species distribution model fitted to the metagenomics plant genus dataset, 326 (81.7%) plant genera responded with high posterior probability (> 95%) to the covariates of either Finland or Sweden based on their beta values (Supplementary Table S6 and Fig. 5), thus contributing to the separation of samples among the countries of origin^30^. 236 plant genera had positive beta values to Sweden, in other words being more common in the samples from Sweden than in the samples from the other countries, while 117 genera had positive beta values to Finland (Supplementary Table S6 and Fig. 5). Yet, the beta values that were supported with high posterior probability were very similar in this dataset (Supplementary Table S6).

For all the other datasets from metagenomics, and for all from metabarcoding and morphological identification of pollen, the genera and families are listed and beta values with high statistical support (over 95% of posterior distribution) from the joint species distribution models are given in the Supplementary Material (Supplementary Tables S7–S19). They total in a very large number of plant, bacterial as well as fungal genera and families that contribute to the separation of samples among different countries (Fig. 5).

### Filtering reveals additional taxa from honey

**Figure 6:**
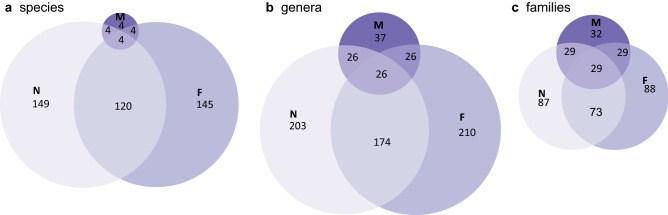
Euler diagrams showing the total number of plant taxa identified from all the samples by morphological identification of pollen (M) and metabarcoding (combined from ITS2, *rbc*L*a* and *trn*L gene regions) of the non-filtered (N) and filtered (F) subsamples as well as the number of shared and unique plant taxa, at different taxonomic levels, for (a) species, (b) genera and (c) families (Supplementary Table S20). The sizes of the graphs are drawn in proportion to the number of taxa.

To examine the role of pollen spores in the taxonomic composition found in honey by DNA, as well as that of other larger particles, we compared non-filtered subsamples with filtered ones. Most taxa detected were shared between non-filtered and filtered samples (Fig. 6). Thus, in terms of species composition, the samples form fully overlapping clusters for all datasets (except fungal ITS; Supplementary Fig. S3). Surprisingly though, some additional taxa were detected in the samples when larger particles were filtered away (Supplementary Table S20). This pattern was clearest for the plant ITS2, where the species detected in the non-filtered treatment proved a smaller part of the species found in the filtered samples. The joint species distribution models confirmed the minor role of sample processing on species detection, as the relative amount of variance explained by the filtering treatment was small (4.4 to 15.8%, Supplementary Table S5). This was especially true for the plant gene regions, for which the filtering treatment explained only 4.4–8.9%.

### Morphological identification complements metabarcoding in the identification of plant taxa

Morphological identification of pollen only captured a small number of plant taxa from the honey samples, as compared to what was found by metabarcoding (Fig. 6) and metagenomics (Table 1). Of all the taxa identified by morphology, all species were also found in both non-filtered and filtered samples analysed by metabarcoding. Yet, some taxa at the genus and family levels which were identified by morphology, were not identified by DNA-based methods (Fig. 6, Supplementary Table S21).

## Discussion

The DNA contents of a honey sample reveals a large number of plant, bacterial and fungal taxa. Plants come from the environment of the bee hive, while the bacteria found in honey can be of many origins, such as bee gut microbiota^22,31^, pathogens of bees^32^, and the environment^21^. Fungi also represent bee pathogens^33^ as well as yeasts able to grow in honey^16^ and fungi from the environment, a possible source being also the microbiota of pollen^17^. Based on our results, plant, bacterial and fungal taxa identified by either metabarcoding or metagenomics can all contribute to distinguishing between honey samples from different countries. DNA-based methods were also shown to be efficient at detecting plant taxa after the removal of pollen, as well as bacterial and fungal taxa after removal of particles of a similar size to pollen, such as spores. This is promising for separating honey samples based on their origin, without relying on pollen.

The three countries examined here, located next to each other in the same ecoregion, present an especially difficult set of countries to be teased apart as they largely share their biotas. Yet looking at the DNA contents of honey, the plant and fungal communities do separate among the countries. For all the metabarcoding datasets, the vast majority of the explained variation was assigned to the country of origin, at similar proportions across the taxonomic groups and taxonomic levels. Among single gene regions, the plant gene region *trn*L provides the most accurate distinction between countries, despite the relatively low number of taxa detected by this marker as compared to other plant gene regions. Of all taxa considered, the composition of plant taxa provided the highest accuracy in differentiating between samples from different countries, a pattern repeated across both metabarcoding and metagenomics, and across different taxonomic levels. Fungi came a close second by metabarcoding.

While plants have previously been used to differentiate among honey of different origins, we found no a priori reason to believe that the plant communities would provide the highest resolution among countries. It is obvious that any plant material entering the hive is from the environment, while bacterial and fungal taxa found in honey originate also from the hives and the bees themselves. The common practices of beekeepers of sharing equipment with compatriots and often using queens from the same country could support the distinction among countries based on bacteria and fungi originating from the hives, too. Yet, our results suggest that bacterial taxa found in honey are shared more widely among the three countries than are plant taxa. Bacterial taxa are also more commonly shared than fungal taxa. On the other hand, combining the data from the different taxonomic groups does not increase the resolution among countries. As a large part of the all the taxa are bacterial, adding plant and fungal taxa to those does not change the result from bacterial taxa only. Thus, our results support the use of both plant and fungal taxa identified by DNA to differentiate among honey samples from different countries. Based on our results, the choice would be to either use metabarcoding with OTUs for plants and fungi or to use metagenomics and opt for the plant taxa. Overall, a wide variety and a large number of plant, bacterial and fungal taxa give support to the distinction among the samples from different countries based on our analyses.

The overall distinction based on the communities of taxa identified by DNA is promising, but there are some topics to be considered, to be developed and to be taken into account when using this approach. A common issue in all research using DNA-based identification of taxa is that some of the sequences cannot be identified to low taxonomic levels due to incomplete reference databases or poor performance of assignment methods^34^. In our study, for the metabarcoding the majority of reads (which were first grouped to OTUs) could be assigned to at least genera, while for metagenomics this was not possible. While metabarcoding is restricted to detect only the taxa the selected primers attach to, metagenomics yields a large number of reads that cannot be taxonomically identified as there are no reference databases covering full genomes for even all higher taxonomic levels. Instead, the reference databases for the metabarcoding regions selected here, although far from complete^35^, contain a comparatively higher coverage of taxa^36–39^.

Instead of assigning the reads to taxa, using OTUs allows all the reads (after quality controls) to be used for analyses. With this highest level of precision, the metabarcoding of *trn*L and *rbc*L*a* for plants and ITS for fungi distinguish the countries better than the data on higher taxonomic groups, except for the metagenomics data on plants. On the other hand, comparing samples at the OTU level does not allow comparison between the datasets generated using different methods. Thus the ever-lasting task to enhance both the reference databases and the methods for taxonomic assignment of sequences continue to be essential to allow for assigning an even higher proportion of sequences to even lower taxonomic units^34^.

Another solution is to use sequence variants, such as zero-radius OTUs (ZOTU^40^) or Amplicon Sequence Variants (ASV^41^). Neither of these approaches uses a pre-defined percentage threshold when determining sequence units or variants. Instead, they take into account amplicon abundance and error rates to discard spurious sequences and retain the biologically meaningful ones and offer the resolution at the level of a phenotype^42^. The downside of these approaches is yet to ascertain the bioinformatics processing is able to remove sequence variants caused by PCR or sequencing errors from real sequences^40,41^. Once that will be overcome, sequence variants would allow comparison among studies. To trace the geographic origin of a sample based on sequence variants, a computer-readable, geolocated sequence database would be needed. Utilising the read archives and sequence databases that are already available, this could be initiated, and the current and future research campaigns would add to this endeavor (see for example^43^). A curated and geolocated sequence database would be useful for a number of purposes, when the origin of a product or specimen needs to be verified^44^.

Based on our analyses a large fraction of variation in the metagenomic datasets was assigned to read count. Thus, the sequencing depth affects which taxa are found and therefore the read count needs to be considered in the analyses. Yet, while the advantage of metagenomics is to be nonselective due to the avoidance of PCR and to the representation of DNA amounts of the original sample being directly sequenced, these advantages are tempered by the fact that a large fraction of the sequences cannot be assigned to any taxon (or only to very high taxonomic levels in our data). Thus, in the end, the number of assigned reads will not reflect actual abundance.

Several methods are currently being tested and developed to identify the regional origin of honey samples. These methods mainly build on different chemical markers such as phenolic compounds, sugars, volatile compounds, organic acids, proteins, amino acids and minerals^6–8^. While all these methods can contribute to reveal the origins of a honey batch, they can be affected by beekeeping and honey processing practises—and even if such impacts could be controlled for, they would require large reference databases to be created^6,45^. On the other hand, new methods using spectroscopic techniques, including nuclear magnetic resonance and infrared light^6^, appear promising in distinguishing honey from different floral sources. Their advantages are their overall simplicity of sample preparation, their speed and relatively moderate costs. Yet, they still lack sufficient databases, the data produced needs to be analysed by multivariate analyses (as do DNA markers) and difficulties remain in separating authentic and adulterated honeys by the resulting classification models^6^.

Compared to the plethora of emerging techniques, the use of DNA-based identification is a well-established method applied for more than two decades^46,47^. Thus, it provides a ready-to-use approach, to be adopted either on its own or in combination with analyses of e.g. organoleptic and/or physiochemical properties of honey, and/or with morphological identification of pollen^13,14^. All of the currently used methods have their disadvantages, and for many methods the caveats are not well known^5,6^. By contrast, the caveats of DNA-based identification are well known, as discussed above. The increased resolution in the identification of taxa achieved by DNA, as shown by our results, is thus a valuable addition to methods for identifying honey origin. Overall, our results support the proposed use of plant identification from honey based on DNA barcoding for various research questions from honey bee behaviour^48–50^ to authentication of honey floral origin^13,14,51,52^, also in regard to the greater diversity of plants detected in comparison to morphological identification of pollen in honey^52^. On top, here we suggest to use the knowledge from microbes identified in honey based on DNA, for differentiating among honey samples from different regional origin. Identifying the bee species or subspecies with DNA barcoding further complements the tool kit for examining the authenticity of honey^14,53^ when considering honey samples from a wider regional range or from a region with specific bee subspecies. As in our study, recently a wide variety of organisms have been detected from honey samples with metagenomics^54,55^. Of further specific interest could be taxa with large amounts of DNA in honey samples, such as the Apis mellifera filamentous DNA virus, offering detailed information of their genetic diversity, possibly leading to defining the honey origin even to the level of the apiary^55^.

Contrary to previous assumptions^6^, our results reveal that DNA-based methods can be used to reliably detect and identify plant taxa in honey samples from which pollen has been removed. This is one of the most important findings of our study. The plant, bacterial and fungal taxa detected are essentially the same with or without pollen (or other particles with a diameter larger than five micrometers), showing that the identification of plants in honey does not need to rely on pollen. Our interpretation is that plant DNA can originate from any other plant material brought to the hive by the bees, and thus DNA from cell fragments and cell organelles from them would contribute to this. Yet, the removal of large particles, which may have a large contribution to the total amount of DNA extracted from a sample, may allow smaller particles to more likely be amplified and sequenced. As a result, additional taxa may be detected after filtering.

Honey is valued by its origin, as are many other food products. Thus it is important the origin can be precisely determined and declared. We show that the identification of multiple taxa by the DNA contained in honey adds new resolution to differentiating between samples of different origins. In fact, the resolution achieved is sufficient to distinguish between honey from three neighboring countries with very similar biota. This indicates that these methods hold great promise for resolving honey origins among different regions globally. Turning to DNA also releases us from the dependency on pollen for honey origins, as the plant taxa can still be identified after the removal of pollen. Overall, the approach of using all DNA in a product for discerning its regional origin could be valuable for other natural products too.

## Methods

### Samples

To examine how DNA can be used to differentiate among honey samples from neighbouring countries, we obtained 19 samples of honey originating from Finland, 19 samples originating from Sweden, and eight samples originating from Estonia. From Finland and Sweden, eight and nine samples, respectively, were obtained directly from beekeepers. Additional samples (eleven from Finland, ten for Sweden and eight for Estonia), were obtained from local stores. These samples are likely comprised of honey from multiple apiaries (Fig. 1, Supplementary Table S1).

### DNA extraction, amplification and sequencing

To prepare the samples for DNA extraction, six subsamples of 10 g were diluted each to 30 ml of DNA pure water (Lonza, AccuGENE Molecular Biology Water) in a 50 ml tube. These subsamples were allowed to dissolve for one hour in + 60 °C. To examine how much of the total information retrievable by DNA in a honey sample is provided by the pollen grains and other larger particles compared to the very small particles, we filtered two of the subsamples through a Ø 5 µm syringe (Sartorius, Minisart) after the honey had completely dissolved. To collect all the tissue material and to remove excess water, all the subsamples were centrifuged for 60 min 8000 *g* (Centrifuge 5810 R, Eppendorf, Germany). Most of the supernatant was discarded and the pellets from two subsamples from either non-filtered or filtered were combined into a 2 ml tube, thus generating two non-filtered and one filtered samples per each original honey sample. These were further centrifuged for 5 min 11000 *g* (Heraeus Pico 21 centrifuge, Thermo Scientific, USA). Remaining supernatant was discarded and the pellets stored in -20 °C until DNA extraction.

Total DNA was extracted from each sample with the DNeasy Plant Mini Kit (Qiagen, Germany) with the following modifications to the protocol. Initially, the pellet was resuspended in 400 µl of buffer AP1, and then 4 µl RNase, 4 µl proteinase K (20 mg/ml, Macherey–Nagel) and one 3 mm tungsten carbide bead was added to each sample tube. The sample was then disrupted 2 × 2 min 30/ rpm (Mixer Mill MM 400, Retsch, Germany). DNA extraction then followed the protocol with the exception of skipping the QIAshredder column step as well as the washing with the buffer AW2 to avoid loss of DNA.

The DNA extracted from one non-filtered and the filtered sample were examined with the DNA metabarcoding of plant, bacterial and fungal taxa. The initial amplifications were done with a total volume of 15 μl, each containing 7.5 μl MyTaq Red Mix (Bioline, London, UK), 4.6 μl DNA- and RNA-free water, 0.45 μl of each primer (10 μM) and 2 μl of DNA extract. PCR cycling conditions were as follows, with primer-specific annealing temperatures (Supplementary Table S2): initial denaturation for 5 min at 95 °C, 35 cycles of 40 s 95 °C (denaturation), 60 s 48 °C (annealing), 30 s 72 °C (extension), and ending with final extension for 5 min at 72 °C. To minimize initial bias of amplification, each reaction was carried out as two replicates. All the amplicons were checked on a 1% agarose gel and imaged with a BioRad imager and when a reaction had not produced a clear band, the PCR was repeated. The successful PCR replicates were combined before library-PCR. Illumina‐specific adapters and unique dual‐index combinations for each sample was used^56^. The library PCR had a total volume of 10 μl, each containing 5 μl MyTaq Red Mix (Bioline, London, UK), 1.2 μl of each primer (2.5 μM) and 2.6 μl of the locus-specific 1^st^ PCR product. Cycling program was the same for all gene regions for the library PCR: 4 min 95 °C, 15 cycles of 20 s 98 °C, 15 s 60 °C, 30 s 72 °C, and ending with 3 min 72 °C. DNA libraries were pooled per gene region and purified using a SPRI bead protocol^57^. The DNA concentration of the cleaned pools were measured with Qubit 2.0 (dsHS DNA Kit, ThermoFisher Scientific). The gene region *trnL* for plants was sequenced on Illumina MiSeq Nano run with v2 chemistry and 2 × 250 cycles. Based on the compatible lengths of the targeted gene regions, *rbc*L*a* and ITS2 for plants pools were combined in equimolar ratios and sequenced on MiSeq sequencing run with v3 chemistry with 300 cycles and 2 × 300 bp paired-end read length. The two 16S rRNA pools (called for short 16Sa and 16Sb) and and the fungal ITS pool were in equimolar ratios for the third MiSeq sequencing run with v3 chemistry with 300 cycles and 2 × 300 bp paired-end read length.

For the metagenomics approach, the DNA extracted from the other non-filtered samplewas used. The DNA was then fragmented to 150 bp pieces and prepared into a sequencing library with NexteraXT Kit DNA Library Preparation Kit (Illumina, Inc.), and sequenced in an Illumina NextSeq 500 Sequencer Mid Output (2 × 150 bp) run. All sequencing was performed by the Functional Genomics Unit^58^ at the University of Helsinki, Finland. To detect possible contamination, we added blank controls, to all the DNA extraction and PCR batches, and sequenced these along with the other samples as well as a blank DNA extraction control also for the whole-genome sequencing.

In the laboratory all the steps before the amplification of DNA were done in a laminar hood wiped with ethanol and cleaned of DNA with 1 h UV light every night and we only used DNA-free tubes, pipet tips and PCR plates as well as DNA-free water.

### Bioinformatic processing of sequences

For the metabarcoding sequences of plants, bacteria and fungi, the bioinformatic processing of reads firstly involved merging the paired ends for each gene region using PEAR^59^ with a minimum overlap of 10 base pairs (bp) and a minimum assembly length of 50 bp. The merged reads were only retained if they contained the expected primers at each end. Primers were then removed using ‘Split_on_Primer.py’ (github:Y-Lammers/Split_on_Primer) before cleaning and filtering using PRINSEQ with a minimum mean quality score of 26 and a minimum length of 50 bp^60^. Dereplication was done using VSEARCH^42^, and the removal of singletons, clustering to OTUs, at 97% and mapping of reads against OTUs using UPARSE^61^. Taxonomic assignations were made using RDP^62^ for all other gene regions but *trn*L, by comparison against a specific reference databases for each gene region. Specifically, ITS2 and *rbc*L*a* for plants were compared against an ITS2 and a *rbc*L*a* reference databases, respectively^37,39^, ITS for fungi against the UNITE fungal ITS reference database^36^ and 16Sa and 16Sb for bacteria against the 16S rRNA reference database^38^. For *trn*L, the taxonomic assignations were made using blastn^63^ against the NCBI reference database^64^ followed by the lowest common ancestor (LCA) analysis, assigning each read to the lowest common ancestor of the set of taxa that it hit in the NCBI database, in MEGAN^65^. To remove possible misassigned reads and false positives, due to tag jumping or contamination, we followed a conservative approach of further filtering for all reads and OTUs (following e.g.^66,67^). This approach included three means, as follows. As small numbers of reads were found in all controls, we subtracted the maximum number of reads for a negative sample from all the samples for each OTU. All samples with fewer than 50 reads in total were removed and all OTUs from a sample with less than 20 reads for that OTU or with less than 0.05% of the total read number (all reads assigned to OTUs) of that sample were removed.

For the metagenomics sequencing, the bioinformatic processing of reads was carried out at University of Helsinki’s Biomedicum Functional Genomics Unit^58^. Overall quality of the sequencing was checked with FASTQC and light quality trimming was performed with trimmomatic^68^. After the reads had passed quality control, taxonomic labels were assigned to sequencing reads using Kraken2^69^. Kraken2 was run against custom-built National Center for Biotechnology Information (NCBI)^70^ non-redundant nucleotide database (NT). The database was built limiting Kraken2 hash table size to 100 GB. To obtain abundance estimates for different species, Kraken2 report files were used as an input for Bracken^71^. Kraken2 results were examined and combined in Pavian^72^. Further filtering of the reads assigned to families, genera and species was done as follows: the number of reads found in the negative sample was subtracted from all the samples for each taxon and all taxa from a sample with less than 20 reads for that taxon were removed.

### Morphological identification of pollen

Thirty grams of each honey sample was sent to a commercial laboratory (FoodQS GmbH, Germany), for the accredited pollen morphology identification procedure to assign the floral origin of honey^9,73^. In this procedure the pollen grains from 10 g of the honey sample are identified, based on reference material and literature^74^ and counted under a microscope. The pollen grains are identified to the lowest possible taxonomic level. For the samples the quantities for the dominant pollen were given as proportions and plant taxa that occurred in the sample as single pollen grains were listed.

### Statistical analyses

To estimate how much the taxonomic composition differed among samples from the three countries, and to establish which markers and methods provided the highest resolution, we used a multistep approach. Below we will outline the methods and models for each question asked at the end of the introduction.

### Does OTU composition separate honey samples from neighboring countries?

To evaluate how well the country of origin explained the biotic contents of the samples, we first analysed OTUs obtained from metabarcoding the three plant gene regions, two bacterial gene regions and one fungal gene region described above. Using OTUs is a useful way to compare samples between marker regions when compared to using a taxonomic assignment (species, genus or family for example) as it removes the variation caused by reference database incompleteness or bias^75^. For comparisons based upon taxonomy (see below), some OTUs cannot be assigned with confidence and are thus omitted from the analyses based taxonomic assignments. For the analyses based on OTUs, we excluded singletons and doubletons (i.e. OTUs that were found in one or two samples only) and given the zero dominance in the data, we used presence-absence data as input. Here we examined both non-filtered and filtered subsamples.

The first approach consisted of applying nonmetric multidimensional scaling (NMDS) to assess whether samples from different countries grouped into distinct clusters based on their OTU composition. We applied the ‘metaMDS’ function of the R package vegan^24^, with 500 iterations and three dimensions. We used the Bray–Curtis dissimilarity index as the dissimilarity metric. The second approach involved assessing the proportion of shared and unique OTUs between samples originating from different countries. For this, we constructed Euler diagrams using the ‘euler’ function of the R package eulerr^25^. The third approach consisted of quantifying how much of the variation in OTU composition among samples was explained by the country of origin. For this, we fitted joint species distribution models^26^ and applied variance partitioning using the R package Hmsc^30^. As response data in the model, we considered a matrix of the presence-absences of the OTUs, and as explanatory variables we included the country of origin and treatment (filtering) as categorical variables and the log-transformed number of reads per sample as a continuous variable. The latter variable was included to account for the differences in “observation effort” among samples with different number of reads. We followed Tikhonov et al.^30^ for evaluating the model fit and convergence, as well for calculating the model’s explanatory power. The explanatory power of the models was measured by calculating Tjur’s R^2^^27^ and AUC^28^ statistics.

To evaluate which of the gene regions shows most separation among samples from different countries, we repeated the above analyses to all the datasets of different gene regions (ITS2, *rbc*L*a* and *trn*L for plants, 16Sa and 16Sb for bacteria and ITS for fungi), and compared the results.

### How well do metabarcoding, metagenomics and morphological identification separate samples from different countries?

To compare how well the three different methods, metabarcoding, metagenomics and morphological identification of pollen, distinguish between honey samples from different countries of origin, we used the same statistical methods as for OTUs from metabarcoding above. To allow for comparison among methods, we compared taxonomic groups of plants, bacteria and fungi. From the metabarcoding data, we combined the data from the different gene regions for each taxonomic group by summing up the reads per each taxon (genus or family) detected by any of the gene regions (ITS2, *rbc*L*a* and *trn*L for plants and 16Sa and 16Sb for bacteria, respectively; for fungi there was only one region, ITS). From the metagenomics data we analyzed plants (Eukaryota: Streptophyta), bacteria (Bacteria) and fungi (Eukaryota: Fungi) as separate datasets. Taxonomic groups detected by metagenomics but not amplified by the metabarcoding markers (e.g. animals) are explicitly left out of the current comparative analyses. As morphological identification of pollen will only identify plants, the data set provided by this method was used for comparison with plants only. For this comparison between morphological and DNA-based methods, we used presence-absence data as for the OTUs, but retained all occurrences regardless of whether the taxon was found in only one or two samples. We compared the data at the taxonomic levels of genus and family. The taxonomic level to be used was chosen as low as possible to offer resolution, yet a level with reliable identifications, as well as a level to which the majority of reads were assigned to (see supplemental Text S1).

To assess which taxa contributed most to the separation of countries, we explored the posterior estimates of the joint species distribution models measuring how each taxa responded to the environmental predictors included in the models. In the HSMC joint species distribution framework used in our study, such parameters are called the beta parameters^76^. For our purpose, we focused on the beta parameters corresponding to the effects of the countries. Positive and negative values of the beta parameters indicate respectively positive and negative responses of the taxa. We considered statistically supported beta parameter values those with posterior probability ≥ 95%.

### Does the removal of pollen and other larger particles by filtering affect which taxa can be detected in honey by DNA?

To examine how much of the information in honey provided by DNA analyses comes from pollen spores and other large particles, we compared the taxa identified from the filtered and non-filtered subsamples. In the above analyses based on OTUs from metabarcoding data, we used both the data from the non-filtered and the filtered subsamples and used the treatment (non-filtered or filtered) as an explanatory variable in the fitting of the joint species distribution models applied to each of the datasets. Furthermore, we compared the shared and unique plant taxa identified by metabarcoding of the non-filtered and the filtered subsamples with those identified by morphological identification of pollen. For this we used Euler diagrams and NMDS plots as above.

## Supplementary Information

Supplementary Information 1.

Supplementary Information 2.

## Data Availability

Additional data is available in the supplementary material, and the datasets generated during the current study are available in the Sequence Read Archive repository, in the BioProject PRJNA662672 (https://www.ncbi.nlm.nih.gov/sra/PRJNA662672).
