# Supplementary material for: DNA traces the origin of honey by identifying plants, bacteria and fungi
Source: Sci Rep. 2021 Feb 26;11:4798. doi: 10.1038/s41598-021-84174-0 (PMC7910293; doi:10.1038/s41598-021-84174-0)
Supplement: Supplementary file 1 — Supplementary Information 1. [file 41598_2021_84174_MOESM1_ESM.pdf]

DNA traces the origin of the honey by identifying plants, bacteria and fungi

Supplementary material

Helena Wirta[1,*], Nerea Abrego[1,2], Kirsten Miller[3,4], Tomas Roslin[1,3], Eero Vesterinen[3,5]

[1]Faculty of Agriculture and Forestry, P.O. Box 27, FI00014, University of Helsinki, Finland

[2]Department of Biological and Environmental Science, P.O. Box 35, FI-40014, University of Jyväskylä, Jyväskylä, Finland

[3]Department of Ecology, P.O. Box 7044, SE750 07, Swedish University of Agricultural Sciences, Sweden

[4]School of Natural and Environmental Sciences, Newcastle University, Newcastle-upon-Tyne, NE1 7RU, United Kingdom

[5]Department of Biology, University of Turku, Finland

[*]Corresponding author

email address of corresponding author: helena.wirta@helsinki.fi

Text S1. *Selection of the taxonomic level for comparison of methods*

To allow comparison among taxa and among methods, we needed to select a suitable taxonomic level, with as much resolution as possible, yet one with reliable identifications and with majority of the reads to be assigned at least to that taxonomic level. It was particularly challenging as simultaneously dealing with data on plants, bacteria and fungi. To estimate the reliability of the taxonomic assignments, we first validated whether the plant taxa detected by DNA methods from

the samples have been found in the countries considered by other records. As the distributions of fungi and bacteria are not well established, this test was restricted to plants. Thus, we checked for all the plant species, genera and families identified, whether they are known to occur in the three countries using occurrence records from GBIF [1], or known to be a common garden plant, cultivated or a commonly imported plant in Finland [2] or Sweden [3].

From metabarcoding the three plant gene regions, the great majority of plant taxa detected were previously known in the three countries. For the species detected by ITS2, *rbc*L*a* and *trn*L 94.4%, 95.5% and 100%, respectively, are known to occur in the studied countries. For genera (98.2%, 89.0% and 100%) and families (100%, 95.6% and 100%, respectively) nearly all taxa are known to occur in the focal countries. From the metagenomics plant species assignations a far lower portion (72.6%) are known to occur in the focal countries. Instead, for genera and families from the metagenomics plant data, 87.0% and 99.1% are known from the three countries. From the morphological identifications, all taxa were found from the occurrence records. Based on this comparison, we considered the genus and family level taxonomic assignments relatively reliable and use them in the analyses. Also in the case of bacteria identified by metabarcoding, the lowest taxonomic level to which OTUs could be identified is the genus [4].

In another study using DNA based identification of pollen in honey, the approach wasto cut off the plant species known not to occur in the study area from the results[5]. Yet, this approach is not suitable when looking for a tool to define the origin of a sample and therefore we keep all taxa found in the analyses.

In regard to the portions of all reads assigned to different taxonomic levels, for metabarcoding, the majority of reads were assigned to genera (51-90% for the different gene regions, data not shown) and to families (67-100%) while for metagenomics 44.0% of the reads were assigned to genera and 45.1% to families. Of the morphological identifications with quantities of the total number of pollen, 30.1% were at genus level and 87.9% at family level. For taxa identified from single pollen

spores 50.9% of the cases were identified to genus and 89.2% to family. Thus, for all the methods a part of the reads, or pollen spores, could be identified to only a higher taxonomic level, meaning part of the data is omitted from analyses when comparing genus or family level data. On the other hand, in the case of bacteria identified by metabarcoding, the lowest taxonomic level to which OTUs could be identified is the genus [4], so we decided to use the levels genera and families in the analyses to compare the morphological identification of pollen, metabarcoding and metagenomics. Yet, for the effect of filtering as well as comparison among morphological identification and metabarcoding, we report also numbers of species identified.

Table S1. Origin of honey samples used in the study.

Sample code	Country	Specific region, if known	Number of apiaries
ES13	Estonia	Länsi-Virumaa	more than one
ES14	Estonia	Länsi-Virumaa	more than one
ES15	Estonia	Harjumaa	more than one
ES16	Estonia	Saarenmaa	more than one
ES17	Estonia	Lõuna-Eesti	more than one
ES18	Estonia		more than one
ES19	Estonia	Valgamaa	more than one
ES20	Estonia	Pandiveren ylänkö	more than one
FI01	Finland	Tammela	more than one
FI02	Finland	Tammela	more than one
FI03	Finland	Lapland	more than one
FI04	Finland		more than one
FI05	Finland	Uusimaa	more than one
FI06	Finland	Uusimaa	more than one
FI07	Finland	Porvoo	more than one
FI08	Finland		more than one
FI09	Finland		more than one
FI10	Finland	South-Finland	more than one
FI11	Finland		more than one
FI12	Finland	Karkkila	one

FI21	Finland	Kalajoki	one
FI22	Finland	Kuhmo	one
FI23	Finland	Inkoo	one
FI24	Finland	Maaninka	one
FI25	Finland	Helsinki	one
FI26	Finland	Urjala	one
FI27	Finland	Ylivieska	one
SE01	Sweden	Östergötland	one
SE02	Sweden	Stockholm	one
SE03	Sweden	Södra Gotland	more than one
SE04	Sweden	Södra Gotland	more than one
SE05	Sweden	Älgö	more than one
SE06	Sweden	Älgö	more than one
SE07	Sweden	Småland	one
SE08	Sweden	Sigtuna	more than one
SE09	Sweden	Småland	one
SE10	Sweden	Småland	more than one
SE11	Sweden	Östergötland	more than one
SE12	Sweden		more than one
SE13	Sweden		more than one
SE14	Sweden	Luleå	one
SE15	Sweden	Norrbotten	one
SE16	Sweden	Norrbotten	one
SE17	Sweden	Lassby	one
SE18	Sweden	Luleå	one
SE19	Sweden		more than one

Table S2. Primers used in the metabarcoding of different gene regions. The 16S rRNA gene region is labelled here for short by 16Sa and 16Sb for the two primer sets, as those are used as names in the current study. In the primer sequence the tag is shown in small letters and the actual gene region specific primer with capital letters.

Gene	Target	Tagged primer name	Primer sequence with tags	Primer reference
ITS2	plants	tagF_ITS2-F	5' - 3' tcgtcggcagcgtcagatgtgtataagagacagATGCGATACTTGGTGTGAAT	[6,7]
		tagR_ITS2-R	5' - 3' gtctcgtgggctcggagatgtgtataagagacagTCCTCCGCTTATTGATATGC	
*rbc*L*a*	plants	tagF_rbcLa-F	5' - 3' tcgtcggcagcgtcagatgtgtataagagacagATGTCACCACAAACAGAGACTAAAGC	[8,9]
		tagR_rbcLa-R	5' - 3' gtctcgtgggctcggagatgtgtataagagacagCGGTCCAYACAGYBGTCCAKGTACC	
*trn*L	plants	tagF_trnL-c	5' - 3' tcgtcggcagcgtcagatgtgtataagagacagCGAAATCGGTAGACGCTACG	[10]
		tagR_trnL-h	5' - 3' gtctcgtgggctcggagatgtgtataagagacagCCATTGAGTCTCTGCACCTATC	
16Sa	bacteria	tagF_16S_515FB	5' - 3' tcgtcggcagcgtcagatgtgtataagagacagGTGYCAGCMGCCGCGGTAA	[11]
		tagR_16S_806RB	5' - 3' gtctcgtgggctcggagatgtgtataagagacagGGACTACNVGGGTWTCTAAT	
16Sb	bacteria	tagF_16S_F-341F	5' - 3' tcgtcggcagcgtcagatgtgtataagagacagCCTACGGGNGGCWGCAG	[12]
		tagR_16S_R-805R	5' - 3' gtctcgtgggctcggagatgtgtataagagacagGACTACHVGGGTATCTAATCC	
ITS	fungi	tagF_ITS3_KYO2	5' - 3' tcgtcggcagcgtcagatgtgtataagagacagAHCGATGAAGAACRYAG	[13]
		tagR_ITS4_KYO3	5' - 3' gtctcgtgggctcggagatgtgtataagagacagCTBTTVCCKCTTCACTCG	

Table S3. Reads at different steps of sequence processing of different gene regions in metabarcoding.

Primer	paired	merged reads	reads passed	reads after	reads after removing	OTUs	reads mapped to
ITS2	3129276	3060503 (97.80%)	2977737 (97.70%)	1584038	123669	855	2725824 (89.2%)
*rbc*L*a*	7987392	7958459 (99.64%)	7880024 (99.81%)	1233276	245201	2000	7401442 (93.0%)
*trn*L	1035934	1029366 (99.37%)	1017632 (99.31%)			427	
16Sa	6948331	6930457 (99.74%)	6893016 (99.96%)	1908622	244313	2971	6377759 (92.1%)
16Sb	4212200	4106298 (97.49%)	4060400 (99.32%)	2609588	144148	1509	2943033 (73.5%)
ITS fungi	6125424	5839102 (95.33%)	5119217 (87.92%)	1976224	170815	2205	4660152 (81.0%)

Table S4. Stress values of the NMDS's for the different datasets for different taxonomic groups and levels, obtained by different methods. The NMDS's were done with three dimensions and maximum 500 iterations and using presence-absence data. For the OTU level analyses singletons and doubletons were excluded, as for the other analyses for this taxonomic level.

Taxonomic group	Taxonomic level	Method	Gene region	stress value
Plants	OTU	Metabarcoding	ITS2	0.1428
Plants	OTU	Metabarcoding	rbcLa	0.1222
Plants	OTU	Metabarcoding	trnL	0.1252
Bacteria	OTU	Metabarcoding	16a	0.1407
Bacteria	OTU	Metabarcoding	16b	0.1325
Fungi	OTU	Metabarcoding	ITS	0.1390
Plants	genera	Morphological		0.1585
Plants	genera	Metabarcoding		0.1337
Plants	genera	Metagenomics		0.0366
Bacteria	genera	Metabarcoding		0.1131
Bacteria	genera	Metagenomics		0.0457
Fungi	genera	Metabarcoding		0.1286
Fungi	genera	Metagenomics		0.0722
Plants	families	Morphological		0.0801
Plants	families	Metabarcoding		0.1590
Plants	families	Metagenomics		0.0367
Bacteria	families	Metabarcoding		0.1056
Bacteria	families	Metagenomics		0.0382
Fungi	families	Metabarcoding		0.1171
Fungi	families	Metagenomics		0.0655

Table S5. Variance partitioning results from the joint species distribution models applied to the datasets obtained for the taxonomic groups and at the examined taxonomic levels by the different methods. Tjur's R^2 values indicate the models' explanatory power and the next columns indicate the proportion of variance explained by the country of origin, the treatment (filtering) and the read count (sequencing depth). Latter columns indicate the proportion of variance explained by the different variables, as a proportion of the total explanatory power of the model for each dataset.

Taxonomic group	Taxonomic level	Method	Gene region	Tjur R^2	AUC	Country	Read count	Treatment	Tjur R^2: Country	Tjur R^2: Read count	Tjur R^2: Treatment	Tjur R^2: unexplained
Plants	OTU	Metabarcoding	ITS2	8.8	78.2	74.4	16.7	8.9	6.5	1.5	0.8	91.2
Plants	OTU	Metabarcoding	*rbc*L*a*	10.8	78.3	77.3	18.3	4.4	8.3	2.0	0.5	89.2
Plants	OTU	Metabarcoding	*trn*L	14	79.9	76.3	18.9	4.7	10.7	2.6	0.7	86.0
Bacteria	OTU	Metabarcoding	16Sa	4.5	72.7	69.8	14.4	15.8	3.1	0.6	0.7	95.5
Bacteria	OTU	Metabarcoding	16Sb	7.2	76.8	76.8	13.1	10.1	5.5	0.9	0.7	92.8
Fungi	OTU	Metabarcoding	ITS2	10.6	81.3	78.8	8.8	12.4	8.4	0.9	1.3	89.4
Plants	genera	Morphological		3.0	65.6	100	NA	NA	3.0	NA	NA	97.0
Plants	genera	Metabarcoding		7.7	76.1	71.6	28.4	NA	5.5	2.2	NA	92.3
Plants	genera	Metagenomics		26.2	87	59.2	40.8	NA	15.5	10.7	NA	73.8
Bacteria	genera	Metabarcoding		3.8	71.6	77.6	22.4	NA	2.9	0.9	NA	96.2
Bacteria	genera	Metagenomics		43.4	91.1	14.3	85.7	NA	6.2	37.2	NA	56.6
Fungi	genera	Metabarcoding		5.7	75.9	79.4	20.6	NA	4.5	1.2	NA	94.3
Fungi	genera	Metagenomics		28	83.5	20.8	79.2	NA	5.8	22.2	NA	72.0
All	genera	Metabarcoding		5	76.8	75.5	24.5	NA	3.8	1.2	NA	95.0
All	genera	Metagenomics		35.9	88.4	22.8	77.2	NA	8.2	27.7	NA	64.1
Plants	families	Morphological		3.9	69.8	100.0	NA	NA	3.9	NA	NA	96.1
Plants	families	Metabarcoding		7.8	74.7	62.3	37.7	NA	4.9	2.9	NA	92.2
Plants	families	Metagenomics		32.9	89.0	47.4	52.9	NA	15.6	17.4	NA	67.1
Bacteria	families	Metabarcoding		5.6	69.2	73.8	26.2	NA	4.1	1.5	NA	94.4
Bacteria	families	Metagenomics		42.6	90.4	17.3	82.7	NA	7.4	35.2	NA	57.4
Fungi	families	Metabarcoding		6.8	77.0	78.2	21.8	NA	5.3	1.5	NA	93.2
Fungi	families	Metagenomics		32.9	85.9	24.0	76.0	NA	7.9	25.0	NA	67.1

Table S6. Plant genera based on metagenomics ordered as in the plot (Fig. 5). The beta values of the taxa to the covariates of the joint distribution model fitted to the data, supported by high posterior probability (>95%) are shown.

order in plot	genus	intercept	Finland	Sweden	Read count
1	*Physcomitrella*	-33.16	1.13	1.41	2.22
2	*Marchantia*	-36.70		1.85	2.21
3	*Equisetum*				
4	*Calciphilopteris*				
5	*Salvinia*				
6	*Zamia*				
7	*Ginkgo*	-36.32		1.87	2.19
8	*Gnetum*				
9	*Araucaria*	-36.27		1.89	2.18
10	*Tetraclinis*	-36.23		1.84	2.18
11	*Cupressus*	-33.55		1.87	2.06
12	*Cryptomeria*	-35.51		2.01	2.20
13	*Chamaecyparis*	-31.82		1.60	2.00
14	*Juniperus*	-39.73		2.33	2.50
15	*Sciadopitys*				
16	*Larix*	-33.30		1.81	2.03
17	*Picea*	-36.76	1.20	1.41	2.50
18	*Pinus*	-29.16	1.44	1.20	2.03
19	*Amborella*	-30.51	1.10	1.28	2.06
20	*Schisandra*	-43.70	0.86	2.58	2.81
21	*Aegopodium*				
22	*Donnellsmithia*				
23	*Angelica*				
24	*Anthriscus*				
25	*Aciphylla*				
26	*Petroselinum*			1.43	
27	*Anginon*			1.41	
28	*Bupleurum*	-20.75	1.04	1.87	1.34
29	*Daucus*	-27.55	1.32		1.94
30	*Panax*	-29.56	0.90	1.78	1.93
31	*Artemisia*				
32	*Chrysanthemum*	-30.90		1.17	2.02
33	*Piofontia*	-21.22			
34	*Diplostephium*	-24.43		1.85	1.51
35	*Helianthus*	-27.85	1.33		1.96
36	*Centaurea*				
37	*Cirsium*				
38	*Carthamus*				
39	*Cynara*	-31.14	1.01		2.16
40	*Cichorium*				

41	*Hypochaeris*	-39.27		2.11	2.45
42	*Crepis*	-39.90		2.02	2.48
43	*Taraxacum*		1.19	1.18	
44	*Lactuca*	-28.03	1.35		1.98
45	*Lobelia*				
46	*Campanula*	-22.93			
47	*Monopsis*	-20.98			
48	*Burmeistera*				
49	*Codonopsis*	-36.23		2.06	2.26
50	*Platycodon*	-38.18		2.49	2.40
51	*Menyanthes*				
52	*Roussea*	-26.06			1.61
53	*Stylidium*				
54	*Viburnum*	-33.69		1.90	2.07
55	*Sambucus*	-38.87		2.15	2.43
56	*Valeriana*				
57	*Patrinia*				
58	*Lonicera*	-40.21		1.99	2.56
59	*Cornus*	-36.47		1.89	2.23
60	*Hydrangea*	-26.07			1.62
61	*Actinidia*	-38.58		1.39	2.47
62	*Clethra*	-21.29			
63	*Shortia*	-21.19			
64	*Diospyros*	-35.29		1.65	2.27
65	*Erica*				
66	*Calluna*				
67	*Rhododendron*				
68	*Monotropa*	-33.09	1.08	1.81	2.16
69	*Pyrola*	-36.43		1.87	2.19
70	*Vaccinium*		1.12	1.33	
71	*Gustavia*	-21.55			
72	*Anagallis*	-36.48		1.88	2.20
73	*Primula*	-19.52		1.62	
74	*Styrax*	-36.71		1.86	2.21
75	*Camellia*		1.11		
76	*Borago*	-37.02		1.87	2.23
77	*Arnebia*	-36.42		1.88	2.19
78	*Lithospermum*	-36.33		1.96	2.24
79	*Echium*	-20.46		1.47	
80	*Cynoglossum*				
81	*Greeneocharis*	-36.74		1.88	2.21
82	*Eremocarya*	-36.92		1.89	2.22
83	*Cryptantha*	-36.88		1.97	2.28
84	*Oreocarya*	-36.31		1.85	2.19
85	*Pectocarya*	-36.66		1.85	2.21
86	*Plagiobothrys*	-36.78		2.00	2.27
87	*Bothriospermum*	-38.48		1.96	2.36
88	*Omphalodes*	-36.45		1.86	2.20

89	*Trigonotis*	-38.73		1.94	2.38
90	*Myosotis*	-26.83			1.67
91	*Ellisia*				
92	*Phacelia*			1.41	
93	*Asclepias*	-35.22		2.15	2.20
94	*Cynanchum*	-40.48		2.28	2.54
95	*Rhazya*	-39.99		2.42	2.52
96	*Thevetia*				
97	*Halenia*				
98	*Guettarda*	-21.02			
99	*Coffea*		1.00		
100	*Avicennia*	-36.79		1.86	2.22
101	*Handroanthus*				
102	*Primulina*				
103	*Dorcoceras*	-36.38		2.18	2.27
104	*Haberlea*	-44.62		2.53	2.83
105	*Marrubium*				
106	*Salvia*	-39.33		2.26	2.47
107	*Ajuga*	-36.86		1.86	2.22
108	*Utricularia*	-41.23		2.37	2.60
109	*Forsythia*	-33.09		1.96	2.04
110	*Ligustrum*	-36.67		1.88	2.21
111	*Osmanthus*	-36.56		1.86	2.20
112	*Syringa*	-29.54		1.50	1.86
113	*Hesperelaea*	-39.77		2.34	2.50
114	*Chionanthus*	-30.03	0.77	1.61	1.93
115	*Olea*	-30.48	1.46	1.21	2.12
116	*Phelipanche*	-35.92		2.02	2.23
117	*Aphyllon*	-39.93		2.41	2.52
118	*Orobanche*	-24.41	1.33	2.03	1.61
119	*Castilleja*	-40.61		2.27	2.60
120	*Melampyrum*				
121	*Neobartsia*	-37.05	0.76	2.06	2.37
122	*Sesamum*	-33.86	1.09	1.59	2.29
123	*Erythranthe*	-30.74	0.99	1.57	2.06
124	*Antirrhinum*	-40.55		2.15	2.53
125	*Plantago*	-22.29		1.59	1.32
126	*Cuscuta*	-30.47		1.42	1.91
127	*Ipomoea*	-27.77	1.33		1.96
128	*Nicotiana*	-22.38	1.04		1.57
129	*Petunia*	-36.23		1.87	2.22
130	*Capsicum*	-33.99	1.08	1.57	2.30
131	*Physochlaina*	-29.27		1.84	1.84
132	*Solanum*		1.00		
133	*Lampranthus*	-21.53			
134	*Delosperma*			1.51	
135	*Sesuvium*	-25.16			1.59
136	*Celosia*	-21.03			

137	*Amaranthus*	-35.55		2.00	2.20
138	*Asteropeia*	-36.82		1.88	2.22
139	*Eriosyce*			-0.96	
140	*Dianthus*	-21.06			
141	*Silene*	-25.99		1.29	1.72
142	*Beta*	-30.49	1.22	1.48	2.08
143	*Spinacia*	-29.48	1.38	1.49	2.04
144	*Chenopodium*	-30.60	1.35	1.47	2.09
145	*Aldrovanda*			1.41	
146	*Pharnaceum*	-23.68			1.38
147	*Nepenthes*	-39.47		2.35	2.48
148	*Fagopyrum*				
149	*Fallopia*	-29.75		1.19	1.91
150	*Rheum*	-33.42		1.83	2.03
151	*Rumex*	-32.67	0.86	2.52	2.07
152	*Monteverdia*				
153	*Citrullus*	-39.27		2.32	2.47
154	*Cucumis*	-26.63	1.58		1.89
155	*Cucurbita*	-29.19	1.53	1.45	2.01
156	*Momordica*	-33.63	1.28	1.80	2.25
157	*Senna*	-35.75		2.08	2.22
158	*Leucaena*	-36.56		2.21	2.28
159	*Acacia*	-39.57		2.32	2.49
160	*Prosopis*	-32.13	1.15	1.52	2.18
161	*Spatholobus*				
162	*Robinia*	-36.40		1.86	2.19
163	*Trigonella*				
164	*Ammopiptanthus*	-25.70		1.26	1.62
165	*Caragana*	-34.63		1.84	2.12
166	*Millettia*	-23.42	0.83	1.78	1.48
167	*Sophora*	-23.54	1.05	2.25	1.49
168	*Lathyrus*				
169	*Lens*		0.72		
170	*Melilotus*	-15.40			1.01
171	*Phaseolus*	-24.38	1.17	1.11	1.69
172	*Abrus*	-25.63	1.17	1.13	1.77
173	*Cajanus*	-32.90	1.56	1.51	2.26
174	*Pisum*	-19.57	0.90		1.38
175	*Arachis*	-28.13	1.35		1.98
176	*Glycine*	-25.31	1.21		1.83
177	*Lotus*		1.12		
178	*Vicia*				
179	*Lupinus*		1.41		
180	*Vigna*		1.38		
181	*Cicer*		1.01		
182	*Medicago*		1.00		
183	*Trifolium*		1.40		
184	*Styphnolobium*	-30.61	0.86	2.25	1.94

185	*Polygala*				
186	*Corylus*	-31.41			1.97
187	*Alnus*	-36.63		1.87	2.21
188	*Betula*	-28.11	1.32	2.16	1.85
189	*Casuarina*	-36.43		1.87	2.19
190	*Castanea*	-36.77		1.93	2.25
191	*Quercus*	-27.72	1.34		1.95
192	*Pterocarya*	-40.33		1.72	2.57
193	*Platycarya*	-19.74			1.23
194	*Juglans*	-29.50	1.22	1.21	2.04
195	*Chrysobalanus*				
196	*Claoxylon*	-21.19			
197	*Ricinus*	-30.80	1.14	1.60	2.05
198	*Vernicia*				
199	*Jatropha*	-30.36	1.23	1.49	2.07
200	*Manihot*	-30.66	1.24	1.74	2.05
201	*Hevea*	-30.41	1.12	1.26	2.09
202	*Euphorbia*	-32.93		1.89	2.02
203	*Excoecaria*	-36.59		1.85	2.21
204	*Hypericum*	-19.53			1.22
205	*Linum*	-25.92	1.22		1.87
206	*Turnera*	-30.34	0.88	2.11	1.94
207	*Passiflora*	-27.44	1.00	1.71	1.80
208	*Salix*	-23.41	1.42	1.12	1.64
209	*Populus*		1.01		
210	*Viola*	-19.13			
211	*Oxalis*	-36.87		1.94	2.26
212	*Barbeya*	-20.98			
213	*Parasponia*	-21.23			
214	*Trema*	-21.46			
215	*Humulus*	-36.46		1.83	2.20
216	*Cannabis*	-34.04	1.25	1.54	2.32
217	*Elaeagnus*				
218	*Hippophae*	-21.94			
219	*Ficus*	-20.66		1.48	
220	*Morus*	-31.93	1.36	1.74	2.16
221	*Ziziphus*	-24.87	1.17	1.12	1.72
222	*Spyridium*	-21.12			
223	*Ceanothus*	-21.49			
224	*Oreoherzogia*	-21.17			
225	*Berchemia*	-20.96		1.46	
226	*Berchemiella*	-20.71		1.47	
227	*Rhamnus*	-21.88		1.60	1.29
228	*Frangula*	-39.18		2.40	2.47
229	*Prunus*		1.00		
230	*Coleogyne*				
231	*Osteomeles*				
232	*Aronia*				

233	*Vauquelinia*	-33.50		1.82	2.04
234	*Dichotomanthes*	-33.33		1.81	2.03
235	*Chaenomeles*	-34.65		1.91	2.13
236	*Pourthiaea*	-35.45		2.04	2.19
237	*Cotoneaster*	-25.17			1.58
238	*Photinia*	-35.40		2.02	2.19
239	*Pyracantha*	-35.35		2.00	2.19
240	*Amelanchier*	-25.64		1.11	1.61
241	*Crataegus*	-39.71		2.33	2.50
242	*Eriobotrya*	-19.12			1.22
243	*Sorbus*	-17.58	0.80		1.13
244	*Pyrus*				
245	*Malus*		0.98		
246	*Exochorda*				
247	*Sorbaria*	-33.26		1.88	2.04
248	*Kelseya*	-36.77		1.85	2.22
249	*Spiraea*	-22.87			1.41
250	*Purshia*	-21.32			
251	*Geum*			1.30	
252	*Sibbaldia*			1.49	
253	*Sibbaldianthe*			1.51	
254	*Alchemilla*			1.56	
255	*Comarum*			1.24	
256	*Fragaria*	-34.25	1.68	1.79	2.34
257	*Potentilla*	-36.48	0.79	2.31	2.33
258	*Filipendula*				
259	*Rubus*	-25.05	1.52	1.79	1.69
260	*Rosa*		1.10		
261	*Sanguisorba*			1.44	
262	*Boehmeria*	-21.15			
263	*Batis*	-29.90		1.44	1.91
264	*Draba*				
265	*Arabis*	-28.00	1.35		1.97
266	*Boechera*	-35.98	0.79	1.69	2.33
267	*Schrenkiella*	-24.20			1.52
268	*Erucastrum*				
269	*Sinapis*	-20.04			1.39
270	*Raphanus*				
271	*Brassica*		1.39		
272	*Bunias*	-38.47		1.94	2.36
273	*Camelina*	-33.20	1.57	1.51	2.28
274	*Capsella*	-18.15	1.01		1.31
275	*Arabidopsis*		1.02		
276	*Barbarea*	-36.64		1.85	2.21
277	*Chorispora*	-36.86		1.85	2.22
278	*Ionopsidium*				
279	*Eutrema*	-33.44	1.05	1.35	2.29
280	*Lepidium*	-36.72		1.85	2.22

281	*Vasconcellea*	-21.53			
282	*Carica*	-28.11	1.34		1.98
283	*Cleomella*			1.45	
284	*Tarenaya*	-28.75	1.03	1.70	1.93
285	*Pelargonium*	-39.66		2.06	2.46
286	*Geranium*	-33.07		2.01	2.10
287	*Helianthemum*				
288	*Bombax*	-44.77		2.52	2.84
289	*Herrania*	-39.27		1.32	2.60
290	*Theobroma*	-27.74	1.34		1.96
291	*Corchorus*	-32.60	1.09	1.96	2.14
292	*Durio*	-27.78	1.09	1.21	1.91
293	*Hibiscus*	-37.02		1.97	2.29
294	*Gossypioides*	-28.53	1.35		2.01
295	*Gossypium*		1.39		
296	*Tilia*	-26.05			1.62
297	*Lythrum*				
298	*Lagerstroemia*	-36.53		2.31	2.33
299	*Ossaea*				
300	*Eucalyptus*	-31.17	0.98	1.35	2.09
301	*Rhodamnia*	-31.51	1.05	1.56	2.12
302	*Campomanesia*	-36.02		1.82	2.17
303	*Syzygium*	-30.05	1.36	1.43	2.04
304	*Chamaenerion*			1.59	
305	*Epilobium*				
306	*Oenothera*				
307	*Mangifera*	-36.43		1.86	2.19
308	*Canarium*	-37.00		1.92	2.23
309	*Zanthoxylum*	-36.52		1.84	2.20
310	*Citrus*	-47.89	0.78	2.28	3.13
311	*Litchi*	-36.63		1.88	2.21
312	*Dimocarpus*	-33.79		1.82	2.06
313	*Dipteronia*				
314	*Acer*	-32.27		1.09	2.11
315	*Handeliodendron*	-36.49		1.88	2.20
316	*Aesculus*	-41.84		2.39	2.64
317	*Parthenocissus*	-38.42		1.94	2.36
318	*Vitis*	-26.00	1.23		1.87
319	*Lophophytum*	-35.44		2.00	2.19
320	*Viscum*	-40.53		2.18	2.59
321	*Aeonium*				
322	*Cynomorium*	-30.40	1.28	2.41	1.97
323	*Ribes*	-24.44			1.51
324	*Hamamelis*	-33.34		1.90	2.04
325	*Paeonia*	-39.24		2.10	2.44
326	*Penthorum*	-21.15			
327	*Heuchera*	-34.37	0.84	1.80	2.22
328	*Spirodela*	-24.87	1.58		1.82

329	*Butomus*	-35.95		2.02	2.23
330	*Stratiotes*	-36.62		1.87	2.20
331	*Allium*	-44.87		2.62	2.85
332	*Asparagus*	-31.18	0.87	1.37	2.10
333	*Scilla*	-36.90		2.21	2.30
334	*Crocus*	-19.32		1.58	
335	*Iris*	-36.62		1.88	2.21
336	*Apostasia*	-31.21			1.96
337	*Cymbidium*	-36.25		1.85	2.18
338	*Erycina*	-35.33		1.33	2.25
339	*Encyclia*	-37.26		1.91	2.25
340	*Gastrodia*	-24.57		2.11	1.50
341	*Dendrobium*	-28.89	0.99	1.60	1.92
342	*Phalaenopsis*	-43.87	0.98	1.73	2.90
343	*Cocos*	-39.46		2.26	2.47
344	*Elaeis*	-25.53	0.86		1.78
345	*Metroxylon*				
346	*Phoenix*	-29.53	1.23	1.72	1.97
347	*Ananas*		1.39		
348	*Carex*			1.34	
349	*Cyperus*				
350	*Eleocharis*	-34.60		1.83	2.11
351	*Joinvillea*	-22.75			1.47
352	*Pleioblastus*	-19.89		1.46	
353	*Phyllostachys*	-39.63		1.95	2.53
354	*Bambusa*				
355	*Oryza*	-27.61	1.34		1.95
356	*Brachypodium*	-28.25	1.35		1.99
357	*Avena*	-31.28	0.87	2.53	1.97
358	*Phleum*				
359	*Festuca*	-36.09		1.83	2.17
360	*Poa*	-23.61			1.45
361	*Dactylis*				
362	*Agrostis*	-23.66		1.90	1.46
363	*Lolium*	-31.98	1.32	2.68	2.07
364	*Leymus*				
365	*Pseudoroegneria*				
366	*Secale*	-37.80		1.93	2.43
367	*Aegilops*	-32.15	1.05	1.55	2.16
368	*Hordeum*	-27.28	1.21	1.65	1.84
369	*Triticum*	-30.16	1.45	1.22	2.10
370	*Eleusine*				
371	*Zaqiqah*				
372	*Zoysia*	-23.41			1.36
373	*Pseudopentameris*	-36.93		1.88	2.23
374	*Sorghum*	-38.27	0.86	1.66	2.54
375	*Saccharum*	-29.37	0.99	1.60	1.95
376	*Zea*	-25.50	1.21	1.15	1.79

377	*Cenchrus*				
378	*Setaria*	-40.00	0.95	1.83	2.63
379	*Panicum*	-31.88	0.93	1.41	2.14
380	*Musa*	-31.40	1.07	1.61	2.09
381	*Zingiber*	-36.73		1.90	2.21
382	*Dioscorea*			-1.34	
383	*Fritillaria*				
384	*Stichoneuron*	-20.26			
385	*Chimonanthus*	-23.48			1.37
386	*Liriodendron*	-33.94		1.91	2.15
387	*Nelumbo*	-26.72	1.23	1.17	1.82
388	*Berberis*	-42.90		2.25	2.69
389	*Eomecon*				
390	*Macleaya*				
391	*Coreanomecon*			1.47	
392	*Papaver*	-27.77	1.34		1.96
393	*Anemone*	-26.38	0.76	2.03	1.66
394	*Helleborus*	-36.85		1.86	2.22
395	*Ranunculus*			1.59	
396	*Leptopyrum*			1.41	
397	*Aquilegia*	-34.24	1.05	2.39	2.20
398	*Nymphaea*	-34.21		1.18	2.17
399	*Selaginella*	-27.64			1.86

Tables S7-S19 provided as a separate supplementary file.

Table S7. Plant genera based on metabarcoding in the alphabetical order. We show the beta parameter values, to the covariates of the joint distribution model fitted to the data, which were statistically supported with high posterior probability (>95%).

Table S8. Plant families based on metabarcoding, as for Table S7.

Table S9. Plant families based on metagenomics, as for Table S7.

Table S10. Plant genera based on morphological identification of pollen, as for Table S7.

Table S11. Plant families based on morphological identification of pollen, as for Table S7.

Table S12. Bacterial genera based on metabarcoding, as for Table S7.

Table S13. Bacterial families based on metabarcoding, as for Table S7.

Table S14. Bacterial genera based on metagenomics, as for Table S7.

Table S15. Bacterial families based on metagenomics, as for Table S7.

Table S16. Fungal genera based on metabarcoding, as for Table S7.

Table S17. Fungal families based on metabarcoding, as for Table S7.

Table S18. Fungal genera based on metagenomics, as for Table S7.

Table S19. Fungal families based on metagenomics, as for Table S7.

Table S20. Comparison of taxa from non-filtered (N) and filtered (F) subsamples, found by different gene regions.

Taxonomic group	Gene region	in N	in F	only in N	only in F
		OTUs			
Bacteria	16Sa	562	831	21 %	46 %
Bacteria	16Sb	511	630	27 %	41 %
Fungi	ITS	566	507	39 %	31 %
Plants	ITS2	335	240	41 %	17 %
Plants	*rbc*L*a*	505	501	19 %	19 %
Plants	*trn*L	142	124	24 %	13 %
		species			
Bacteria	16Sa				
Bacteria	16Sb				
Fungi	ITS	86	60	41 %	15 %
Plants	ITS2	104	102	18 %	17 %
Plants	*rbc*L*a*	51	47	15 %	7 %
Plants	*trn*L	7	5	29 %	0 %
		genera			
Bacteria	16Sa	223	303	16 %	38 %
Bacteria	16Sb	236	279	20 %	32 %
Fungi	ITS	101	70	41 %	14 %

Plants	ITS2	137	132	20 %	16 %
Plants	*rbc*L*a*	129	127	12 %	11 %
Plants	*trn*L	36	37	12 %	14 %
		families			
Bacteria	16Sa	126	142	15 %	25 %
Bacteria	16Sb	118	126	17 %	22 %
Fungi	ITS	74	57	36 %	17 %
Plants	ITS2	52	49	17 %	12 %
Plants	*rbc*L*a*	78	82	9 %	13 %
Plants	*trn*L	46	44	12 %	8 %

Table S21. Comparison of plant taxa from non-filtered and filtered subsamples by metabarcoding (combined from ITS2, *rbc*L*a* and *trn*L) and morphologically identified pollen.

	Methods combined	Morphology		Non-filtered		Filtered	
Taxonomic level	all	all	unique by	all	unique by	all	unique by
species	174	4	0 %	149	19 %	145	17 %
genus	250	37	30 %	203	14 %	210	17 %
family	105	32	9 %	87	16 %	88	17 %

Figure S1. Euler diagrams showing the shared and unique OTUs found in the honey samples among the three different countries. The sizes of the circles present the total amount of OTUs in each country and the numbers give the total numbers for each country and the totals for each overlap. The panels show the DNA data from different gene regions: plants a) ITS2, b) rbcLa, c) trnl, bacteria; d) 16Sa, e) 16Sb, and fungi; f) ITS. ES stands for Estonia, FI for Finland and SE for Sweden. The sizes of the graphs are in proportion to other gene regions.

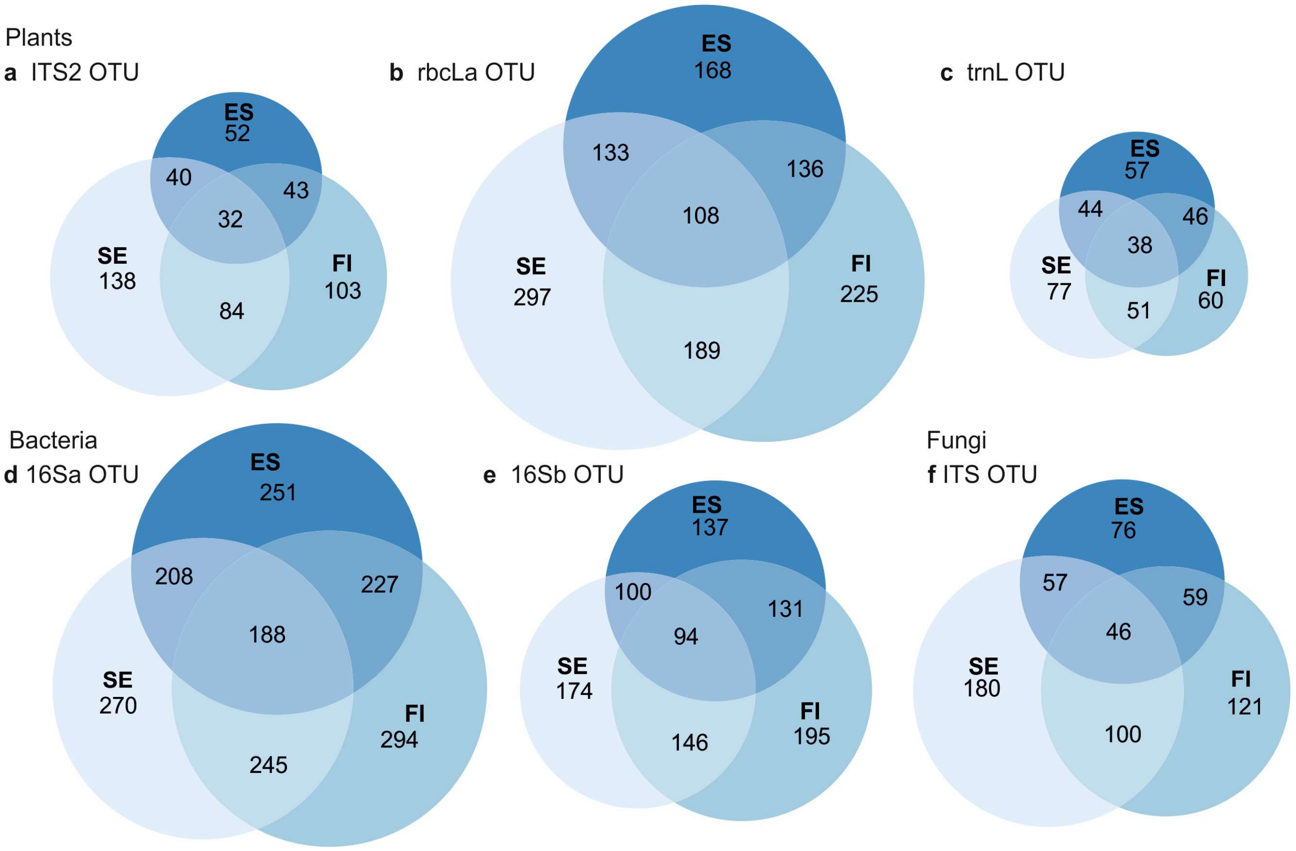

Figure S2. The nonmetric multidimensional scaling plots (NMDS) for plant, bacterial and fungal family community similarities among samples originating from the three different countries. The ellipses confine 75% of the data points. Stress values for the different gene region analyses are given in table S4. The panels show the data from different methods morphology (a), metabarcoding (b-d) and metagenomics (e-g), for different taxonomic groups: a), b) and e) plants, c) and f) bacteria and d) and g) fungi. The samples from Estonia (ES) are shown in gray, from Finland (FI) in blue and from Sweden (SE) in light orange.

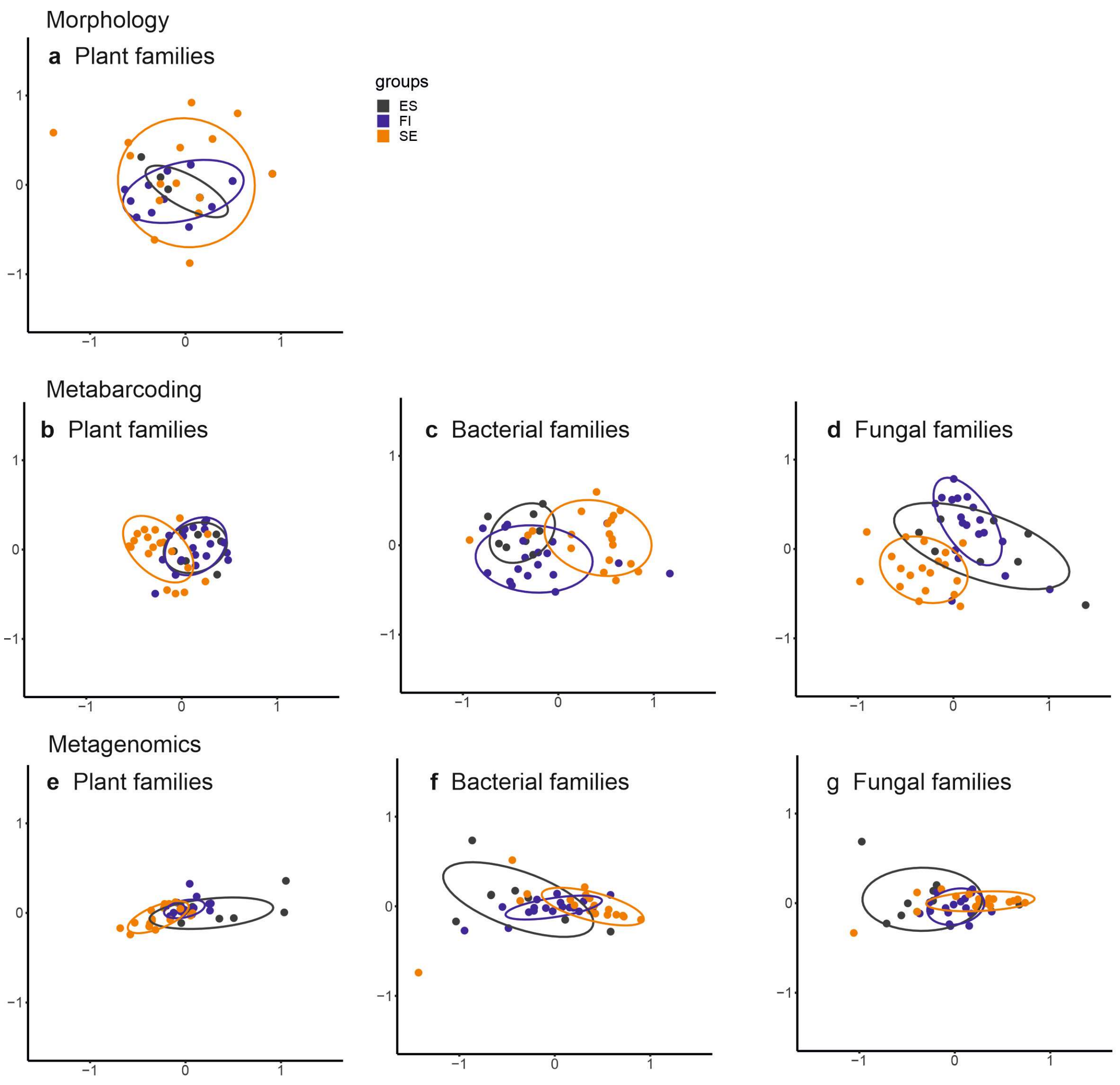

126

Fig. S3. The nonmetric multidimensional scaling plots for plant, bacterial and fungal OTU community similarities among non-filtered (N; purple) and filtered (F; turquoise) samples. The ellipses confine 75% of the data points. Stress values for the different gene region analyses are given in Table S4. The panels show the data from different gene regions: plants: a) ITS2, b) *rbc*L*a*, c) *trn*L, bacteria: d) 16Sa, e) 16Sb, and fungi: f) ITS.

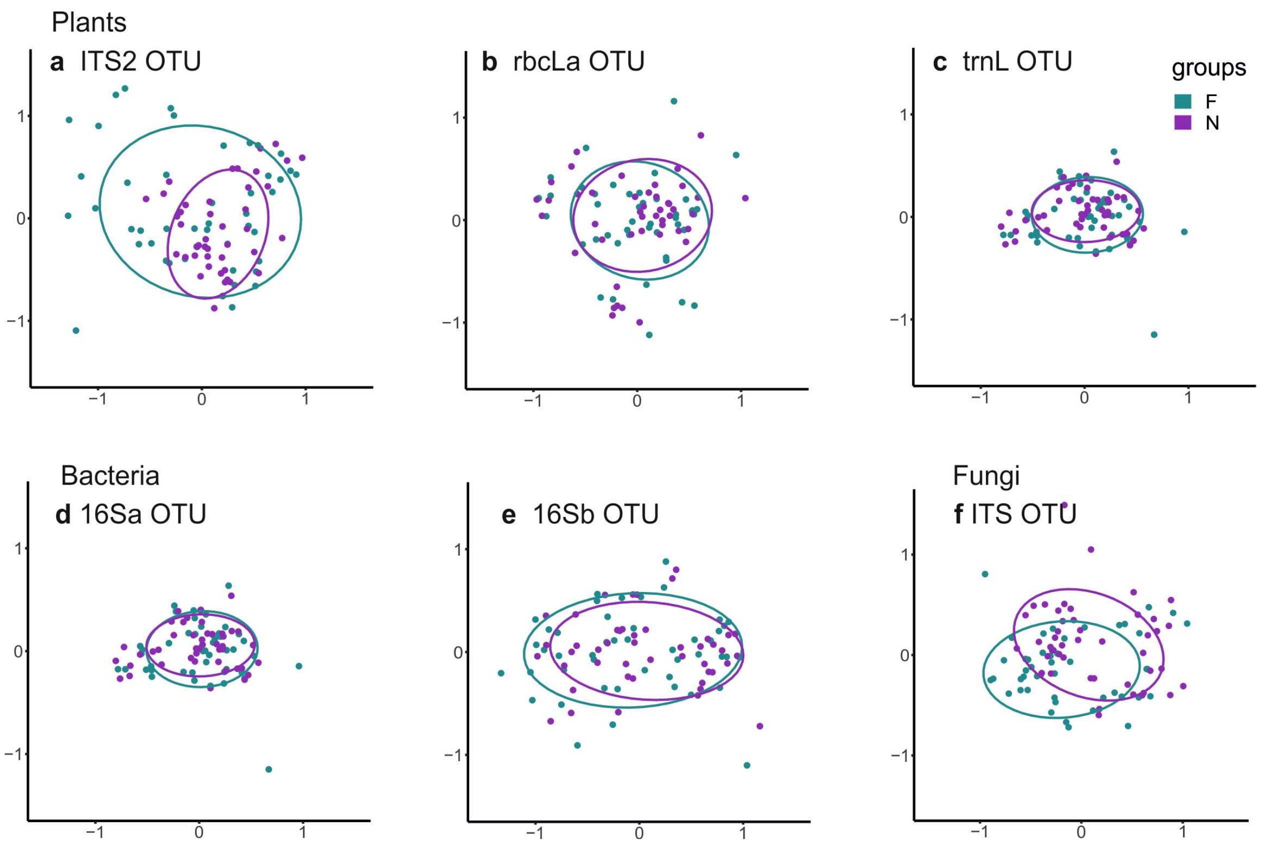

References used in supplementary material

1. GBIF. GBIF.org (17 March 2020) GBIF Occurrence Download. (2020) doi:doi.org/10.15468/dl.wibash.

2. Räty, E. *Viljelykasvien nimistö 2017*. (Puutarhaliitty ry., 2017).

3. Aldén, B. & Ryman, S. *Våra kulturväxters namn : ursprung och användning*. (Forskningsrådet Formas, 2009).

4. Wang, Q., Garrity, G. M., Tiedje, J. M. & Cole, J. R. Naïve Bayesian classifier for rapid assignment of rRNA sequences into the new bacterial taxonomy. *Appl. Environ. Microbiol.* **73**, 5261–5267 (2007).

5. Hawkins, J. *et al.* Using DNA metabarcoding to identify the floral composition of honey: A new tool for investigating honey bee foraging preferences. *PLoS One* **10**, e0134735 (2015).

6. Chen, S. *et al.* Validation of the ITS2 Region as a Novel DNA Barcode for Identifying Medicinal Plant Species. doi:10.1371/journal.pone.0008613.

7. White, T., Bruns, T., Lee, J. & Taylor, M. Amplification and direct sequencing of fungal ribosomal RNA genes for phylogenetics. in *PCR protocols: a guide to methods and applications* 315–322 (Academic Press, 1990).

8. Levin, R. A. *et al.* Family-level relationships of Onagraceae based on chloroplast *rbc L* and *ndh F* data. *Am. J. Bot.* **90**, 107–115 (2003).

9. Ivanova, N. V, Kuzmina, M. L., Braukmann, T. W. A., Borisenko, A. V & Zakharov, E. V. Authentication of Herbal Supplements Using Next-Generation Sequencing. (2016) doi:10.1371/journal.pone.0156426.

10. Taberlet, P. *et al.* Power and limitations of the chloroplast trnL (UAA) intron for plant DNA barcoding. doi:10.1093/nar/gkl938.

11. Walters, W. *et al.* Improved Bacterial 16S rRNA Gene (V4 and V4-5) and Fungal Internal Transcribed Spacer Marker Gene Primers for Microbial Community Surveys crossmark Downloaded from. (2015) doi:10.1128/mSystems.00009-15.

12. Pr Herlemann, D. *et al.* Transitions in bacterial communities along the 2000 km salinity gradient of the Baltic Sea. *ISME J.* **5**, 1571–1579 (2011).

13. Toju, H., Tanabe, A. S., Yamamoto, S. & Sato, H. High-Coverage ITS Primers for the DNA-

Based Identification of Ascomycetes and Basidiomycetes in Environmental Samples. *PLoS One* **7**, 40863 (2012).
