# Supplementary material for: DNA traces the origin of honey by identifying plants, bacteria and fungi
Source: Sci Rep. 2021 Feb 26;11:4798. doi: 10.1038/s41598-021-84174-0 (PMC7910293; doi:10.1038/s41598-021-84174-0)
Supplement: Supplementary file 2 — Supplementary Information 2. [file 41598_2021_84174_MOESM2_ESM.pdf]

Table S7. Plant genera based on metabarcoding in the alphabetical order. We show the beta parameter values, to the covariates of the joint distribution model fitted to the data, which were statistically supported with high posterior probability (>95%).

Genus	intercept (Estonia)	Finland	Sweden	Read count
Abies				
Acer				
Achillea				
Actaea				
Adenocarpus	-14.66			
Aegopodium			-0.97	
Aesculus	-17.18			1.25
Aethionema				
Aizopsis				
Alliaria				
Allium	-17.52		1.46	1.29
Alnus				
Alopecurus				
Alternanthera				
Amaranthus				
Amelanchier	-17.16		1.63	1.27
Ampelocissus				
Andryala		-1.33		
Anemone	-14.49			1.19
Angelica				
Anthoxanthum				
Anthriscus	-14.46			1.16
Antidesma				
Arabidopsis				
Arctium				
Artemisia				
Asparagus				
Astragalus				
Atriplex				
Azara				
Barbarea	-12.73			
Begonia				
Bellis				
Berberis			1.22	
Betula				
Brassica				
Broussaisia	-15.62			
Bunias				
Buxus				

Calluna				0.91
Caltha				
Campynema				
Cannabis		-1.34	-1.18	
Capsella				
Caragana				
Cardamine				
Carduus				
Carex	-13.54			
Carum				
Centaurea				
Ceratodon				
Chaerophyllum				
Chamerion				
Chelidonium				
Chenopodium				
Cicuta				
Cirsium	-12.22			0.94
Clematis	-15.74			
Coccomyxa				
Comarum	-11.16	1.32	1.68	0.89
Comptonia				
Convolvulus				
Coriandrum				
Cornus	-13.22			1.02
Cosmos				
Crataegus	-16.83		1.86	1.28
Crepis				
Crocus				
Cryptotaenia				
Cucumis			-1.23	
Cynoglossum				
Cysticapnos				
Dasiphora	-14.96			
Daucus	-12.57			
Deutzia				
Diplotaxis				
Dovyalis	-15.46		1.47	1.12
Echium				
Epilobium				
Equisetum				
Erodium				
Erysimum				
Euphrasia				
Fagopyrum				
Fagus				

Ficaria				
Ficus				
Filipendula				
Flueggea				
Fragaria				
Frangula	-12.72			
Gagea	-11.83			
Galega		-1.38	-1.20	
Galeopsis				
Galium				
Geranium	-12.49			0.96
Geum				
Glycine				
Habenaria				
Hazenia				
Helianthemum				
Helianthus				
Heracleum				
Hesperis	-14.99			
Hesperomannia				
Hieracium				
Hilleria				
Hordeum				
Hosta				
Hydrangea	-16.83			1.25
Hydrophyllum	-13.70			
Hylotelephium				
Hypericum				
Hypochaeris				
Impatiens				
Juniperus	-16.39		1.41	1.23
Lactuca				
Lagenaria				
Lamium				
Lathraea		-1.39	-1.21	
Lathyrus				
Leucanthemum	-13.73			
Linaria				
Liquidambar				
Lonicera			0.94	
Lotus	-14.48			
Lupinus		1.29		
Lysimachia				
Lythrum				
Maesopsis				
Malus	-15.85			1.18

Malva				
Mathewsia				
Matricaria				
Meconopsis				
Medicago				
Melampyrum				
Melilotus				
Menyanthes				
Menziesia				
Moricandia				
Musa		-1.37	-1.19	
Myosotis	-13.77		1.22	1.03
Myrrhis				
Nerium				
Noccaea	-14.20			
Noronhia				
Nymphaea	-15.99			1.18
Odontites				
Olea	-20.19	1.36	1.94	1.60
Omphalodes	-16.04			1.19
Origanum				
Ornithogalum				
Orthilia		-1.41	-1.25	
Oxalis				
Paeonia				
Papaver				
Pastinaca				
Pelargonium				
Peucedanum				
Phacelia				
Philadelphus	-15.08			
Phleum	-14.70		1.34	1.15
Phragmites				
Picea	-11.15			0.92
Pinus	-11.66			1.08
Pisum				
Plantago	-15.56			1.20
Platanthera	-13.83			
Polemonium				
Polygonatum				
Populus	-15.25		1.34	1.15
Potentilla	-12.87			
Prunella				
Prunus				
Pseudostichococcus				
Pyrus				

Quercus				
Ranunculus	-11.45			
Raphanus				
Rhamnus	-11.91			0.99
Rhododendron				
Rhus				
Ribes	-16.00		1.42	1.22
Robinia				
Rorippa				
Rosa			1.31	
Rubus				1.01
Rumex	-13.83			
Salix				
Sambucus	-13.35			
Saponaria				
Scilla	-15.80			
Scorzoneroides				
Scrophularia				
Sedum				
Senecio				
Senna				
Silene				
Sinapis				
Solanum				
Solidago				
Sonchus				
Sorbaria	-12.94			
Sorbus				0.97
Spergula				
Sphagnum				
Spiraea				
Stellaria				
Sterculia	-10.16			
Streblus				
Streptanthus		-1.27		
Succisa				
Symphoricarpos	-13.28		1.35	
Symphyotrichum				
Syringa	-17.72		1.47	1.30
Tamamschjanella				
Tanacetum				
Taraxacum	-15.38			1.41
Tetrastigma				
Thalictrum				
Thunbergia				
Tilia				

Trebouxia				
Trifolium				
Trigonella				
Tripleurospermum				
Triticum				
Ulmus		-1.41	-1.25	
Urtica				
Vaccinium		1.57	1.06	
Valeriana				
Veronica				
Viburnum	-16.99			1.23
Vicia			-1.38	
Viola	-12.23			
Withania				
Vitis	-13.50			
Zea				

Table S8. Plant families based on metabarcoding, shown as for Table S7.

Family	intercept (Estonia)	Finland	Sweden	Read count
Acanthaceae				
Aceraceae	-15.57			1.35
Adoxaceae	-14.39		1.16	1.16
Alismataceae				
Altingiaceae				
Amaranthaceae				
Amaryllidaceae	-18.54		1.27	1.39
Anacardiaceae				
Apiaceae	-12.90			1.14
Apocynaceae	-16.93			
Asparagaceae	-17.41			
Asteraceae				
Balsaminaceae				
Begoniaceae				
Berberidaceae				
Betulaceae	-11.38			1.03
Bignoniaceae				
Boraginaceae	-16.56			1.33
Brassicaceae				
Buxaceae				
Campanulaceae				
Campynemataceae				
Cannabaceae		-1.02	-1.33	
Capparaceae				
Caprifoliaceae				
Caryophyllaceae				
Casuarinaceae				
Cistaceae				
Coccomyxaceae				
Comandraceae				
Convolvulaceae				
Cordiaceae				
Cornaceae				
Crassulaceae				
Cucurbitaceae				
Cupressaceae	-16.71		1.16	1.27
Cyperaceae	-14.32			
Diapensiaceae				
Ditrichaceae				
Equisetaceae				
Ericaceae				
Fabaceae				

Fagaceae			
Geraniaceae			
Goodeniaceae			
Grossulariaceae	-16.17	1.05	1.27
Hippocastanaceae	-18.32		1.37
Hyacinthaceae		1.22	
Hydrangeaceae	-18.70		1.43
Hydrophyllaceae	-16.10		1.24
Hypericaceae			
Icacinaceae			
Iridaceae			
Juglandaceae			
Lamiaceae			
Liliaceae	-12.34		
Lythraceae			
Malvaceae			
Melastomataceae			
Menyanthaceae			
Metteniusaceae	-17.62		
Moraceae			
Musaceae			
Myricaceae	-16.73		
Not.assigned			
Nymphaeaceae	-17.49		1.33
Oleaceae		1.14	
Onagraceae		-1.21	
Orchidaceae			
Orobanchaceae			
Oxalidaceae			
Paeoniaceae			
Papaveraceae			
Petenaeaceae			
Phyllanthaceae			
Phytolaccaceae			
Picrodendraceae	-16.61		
Pinaceae			
Plantaginaceae			
Poaceae			
Polemoniaceae			
Polygonaceae			
Primulaceae			
Ranunculaceae			
Resedaceae			
Rhamnaceae			
Rosaceae			
Rubiaceae			

Rutaceae			
Salicaceae			
Salvadoraceae	-12.74		1.06
Sapindaceae			
Scrophulariaceae			
Solanaceae			
Sphagnaceae			
Symplocaceae			
Theaceae			
Thymelaeaceae			
Ulmaceae		-1.13	
Urticaceae			
Violaceae			
Vitaceae	-14.10		
Xanthorrhoeaceae			

Table S9. Plant families based on metagenomics, shown as for Table S7.

Genus	intercept (Estonia)	Finland	Sweden	Read count
Acanthaceae	-47.78		1.73	2.96
Actinidiaceae	-49.77		1.52	3.22
Adoxaceae	-49.03		2.22	3.15
Aizoaceae	-33.36		1.15	2.15
Amaranthaceae	-38.91		1.49	2.50
Amaryllidaceae	-51.73		2.06	3.36
Amborellaceae	-35.07	1.09	1.27	2.37
Anacardiaceae	-45.16		1.82	2.86
Apiaceae	-31.57	1.45		2.22
Apocynaceae	-36.48	1.14	2.29	2.41
Araceae	-26.95	1.72		1.97
Araliaceae	-34.32	0.75	1.68	2.26
Araucariaceae	-47.02		1.70	2.91
Arecaceae	-31.26	1.44		2.20
Aristolochiaceae	-47.38		1.72	2.93
Asparagaceae	-35.06	0.79	1.41	2.37
Asteraceae		1.29		
Asteropeiaceae	-47.37		1.70	2.93
Balanophoraceae	-44.42		1.90	2.82
Barbeyaceae	-25.76			1.54
Bataceae	-36.21		1.30	2.34
Berberidaceae	-55.00		2.21	3.53
Betulaceae	-34.84	1.05	1.69	2.35
Bignoniaceae	-45.87		1.80	2.90
Boraginaceae	-41.86		1.63	2.70
Brassicaceae		1.77		
Bromeliaceae		1.77		
Burseraceae	-47.15		1.71	2.91
Butomaceae	-45.06		1.87	2.85
Cactaceae				
Calycanthaceae	-27.67			1.66
Campanulaceae	-41.73		1.19	2.78
Cannabaceae	-39.63	1.23	1.60	2.70
Caprifoliaceae	-46.36		1.90	3.02
Caricaceae	-32.01	1.45		2.25
Caryophyllaceae	-32.88		1.49	2.19
Casuarinaceae	-46.83		1.76	2.94
Celastraceae	-35.77			2.26
Chenopodiaceae	-31.47	1.45		2.21
Chrysobalanaceae	-43.57		1.39	2.81
Cistaceae	-23.89			
Cleomaceae	-34.43	0.92	1.72	2.32

Clethraceae	-25.84			1.54
Convolvulaceae	-31.76	1.44		2.23
Cornaceae	-47.12		1.78	2.96
Crassulaceae	-44.62			2.86
Cucurbitaceae	-30.32	1.61	1.25	2.15
Cupressaceae	-58.72		2.42	3.83
Cynomoriaceae	-35.81	1.01	2.16	2.36
Cyperaceae	-42.25		1.98	2.74
Diapensiaceae	-25.58			1.53
Dioscoreaceae			-1.04	
Droseraceae	-21.35			
Ebenaceae	-43.00		1.53	2.80
Elaeagnaceae	-47.37		1.83	2.99
Equisetaceae				
Ericaceae		1.21	1.02	
Euphorbiaceae	-31.08	1.43		2.19
Fabaceae		1.75		
Fagaceae	-31.56	1.44		2.22
Funariaceae	-38.49	1.10	1.34	2.59
Gentianaceae	-44.83		1.71	2.81
Geraniaceae	-44.70	0.83	2.01	2.94
Gesneriaceae	-48.15		1.80	3.17
Ginkgoaceae	-47.69		1.73	2.95
Gnetaceae				
Grossulariaceae	-30.10			1.90
Hamamelidaceae	-45.56		1.86	2.89
Hyacinthaceae	-51.48		2.28	3.31
Hydrangeaceae	-33.56		1.00	2.15
Hydrocharitaceae	-44.66		1.76	2.82
Hydrophyllaceae	-21.64			
Hypericaceae	-23.96			1.54
Iridaceae	-46.34		1.95	2.95
Joinvilleaceae	-26.98			1.76
Juglandaceae	-34.81	1.49	1.23	2.42
Lamiaceae	-49.51		1.67	3.26
Lauraceae	-46.89		1.76	2.94
Lecythidaceae	-26.08			1.56
Lentibulariaceae	-51.97		2.19	3.34
Liliaceae	-49.06		2.00	3.13
Linaceae	-28.30	1.42		2.03
Lythraceae	-32.85		2.01	2.12
Magnoliaceae	-41.36		1.74	2.67
Malvaceae		1.30		
Marchantiaceae	-47.55		1.71	2.94
Melastomataceae				
Meliaceae	-47.59		1.75	2.94

Menyanthaceae				
Molluginaceae	-28.64			1.73
Moraceae	-37.15	1.25	1.68	2.52
Musaceae	-36.64	0.99	1.57	2.45
Myrtaceae	-38.13	1.56	1.48	2.62
Nelumbonaceae	-29.80	1.24	1.08	2.03
Nepenthaceae	-50.58		2.17	3.24
Nymphaeaceae	-43.40		1.29	2.79
Oleaceae	-34.92	1.49	1.23	2.43
Onagraceae		0.87		
Orchidaceae	-34.28	1.12	1.23	2.34
Orobanchaceae	-26.62	1.30	1.42	1.82
Oxalidaceae	-46.91		1.76	2.94
Paeoniaceae	-50.51		1.97	3.21
Papaveraceae	-31.92	1.44		2.24
Passifloraceae	-31.82	0.93	1.84	2.12
Pedaliaceae	-40.49	1.02	1.67	2.75
Penthoraceae	-25.44			1.52
Phrymaceae	-35.59	0.89	1.55	2.39
Phyllanthaceae	-47.79		1.72	2.96
Pinaceae	-31.70	1.45		2.23
Plantaginaceae	-56.46		2.40	3.64
Poaceae	-28.31	1.45		2.03
Podocarpaceae	-47.76		1.70	2.96
Podostemaceae	-26.11			1.56
Polygalaceae	-21.07			
Polygonaceae	-27.03		1.23	1.80
Primulaceae	-52.28		2.23	3.36
Pteridaceae	-45.74		1.80	2.89
Ranunculaceae	-34.68	1.08	1.70	2.34
Rhamnaceae	-25.14	1.08		1.76
Rhizophoraceae	-47.49		1.72	2.94
Rosaceae		1.33		
Rousseaceae	-31.15			1.96
Rubiaceae		1.03		
Rutaceae	-57.64		2.28	3.79
Salicaceae		1.32		
Salviniaceae	-21.67			
Sapindaceae	-29.03		1.13	1.92
Saxifragaceae	-42.56	0.72	1.77	2.78
Schisandraceae	-52.83		2.38	3.44
Sciadopityaceae				
Selaginellaceae	-32.03			2.15
Solanaceae		1.31		
Stemonaceae	-24.41			
Stylidiaceae				

Styracaceae	-47.09		1.71	2.91
Tamaricaceae	-25.58			1.53
Theaceae		1.29		
Thymelaeaceae	-47.35		1.72	2.93
Ulmaceae	-26.02			1.56
Urticaceae	-46.87		1.83	2.96
Violaceae	-44.88		1.79	2.83
Viscaceae	-50.21		2.12	3.26
Vitaceae	-27.85	1.41		2.00
Zamiaceae				
Zingiberaceae	-47.71		1.73	2.95

Table S10. Plant genera based on morphological identification of pollen, shown as for Table S7.

Genus	intercept (Estonia)	Finland	Sweden
Achillea	-1.18		
Alnus	-1.36		
Arctium	-1.81		
Artemisia			
Betula			
Brassica			
Calluna	-1.61		
Cyanus			
Cynoglossum	-1.03		
Echium	-1.07		
Erica	-0.79		
Fagopyrum			-0.94
Filipendula	1.22		
Fragaria	-1.58		
Genista	-1.82		
Gleditsia			
Juniperus	-1.78		
Ligustrum			
Lotus	-0.88		
Onobrichis	-1.30		
Phacelia	-0.78		
Pinus	-1.33		
Pirus			
Prosopis	-1.63		
Prunus			
Quercus			
Rubus			
Rumex	-0.90		
Serratulla	-0.98		
Sinapis	-0.72		
Taraxacum	-1.42		
Taxus	-1.46		
Tilia	-0.71		
Trifolium	1.49		
Ulex	-1.79		
Vaccinium	-1.80		
Vicia			

Table S11. Plant families based on morphological identification of pollen, shown as for Table S7.

Family	intercept (Estonia)	Finland	Sweden
Aceraceae	-0.99		
Apiaceae	1.00		
Asteraceae	0.68		
Balsaminaceae	-1.68		
Betulaceae	-0.99		
Boraginaceae	2.04		
Brassicaceae	-1.90		
Caryophyllaceae	-2.22		
Chenopodiaceae	-1.77		
Cornaceae	-0.70		
Cyperaceae			
Ericaceae	-1.35		
Fabaceae	-0.74		
Fagaceae	-3.66		
Hippocastanaceae	-3.69		
Hydrophyllaceae	-3.72		
Lamiaceae	-3.67		
Laureaceae	-3.70		
Liliaceae	-3.69		
Malvaceae	-3.69		
Oleaceae	-3.63		
Onagraceae	-3.71		
Papaveraceae	-3.64		
Pinaceae	-3.65		
Plantaginaceae	-3.71		
Poaceae	-3.67		
Polygonaceae	-3.66		
Rhamnaceae	-3.73		
Rosaceae	-3.72		
Salicaceae	-3.71		
Taxaceae	-3.73		
Violaceae	-3.67		

Table S12. Bacterial genera based on metabarcoding, shown as for Table S7.

Family	intercept (Estonia)	Finland	Sweden	Read count
Achromobacter			-0.7890	
Acidicaldus				
Acidiphilium				
Acidisoma				
Aciditerrimonas				
Acidothermus				
Acinetobacter				
Actinomyces			-0.7730	
Aequorivita				
Aerococcus			-0.8095	
Aeromicrobium				
Aeromonas				
Alcaligenes			-0.7939	
Alicyclobacillus				
Alistipes				
Alkaliphilus				
Alloprevotella			-0.8091	
Altererythrobacter				
Ammoniibacillus				
Ammoniphilus				
Amphibacillus				
Anaerobacter			-0.7426	
Anaerococcus				
Anaeromyxobacter				
Anaerorhabdus				
Anaerosalibacter				
Anaerotruncus				
Anaerovorax				
Anaplasma				
Ancylobacter				
Anderseniella				
Aneurinibacillus			-0.7239	
Anoxybacillus			-0.6986	
Anoxybacter				
Aquabacterium				
Aquaspirillum				
Aquicella				
Aquihabitans				
Aquipuribacter				
Aquisphaera				
Arcicella				
Arcobacter				

Arenicella				
Arenimonas				
Aridibacter			-0.8003	
Arsenophonus				
Arthrobacter			-0.7538	
Asaia				
Aureimonas				
Azomonas				
Azonexus				
Azospirillum				
Bacillariophyta				
Bacillus	20.6016			-1.7726
Bacteroides				
Bdellovibrio				
Beijerinckia				
Belnapia			-0.7767	
Bergeyella				
Bifidobacterium				
Blastococcus				
Blastopirellula				
Blautia				
Bombella				
Bordetella				
Brachybacterium				
Bradyrhizobium		-0.6558		
Brevibacillus				
Brevibacterium				
Brevifollis				
Brevundimonas				
Brochothrix				
Buchnera				
Burkholderia				
Byssovorax			-0.8003	
Candidatus Carsonella			-0.8103	
Capnocytophaga			-0.7835	
Carnimonas				
Carnobacterium				
Caulobacter				
Cedecea				
Cellulomonas			-0.8071	
Cellulosilyticum				
Cellvibrio				
Chelatococcus				
Chitinophaga				
Chlorophyta				
Chondromyces			-0.7905	

Chryseobacterium				
Chryseolinea				
Chthonomonas Armatimonadetes_gp3				
Cloacibacterium			-0.7414	
Clostridium III				
Clostridium IV				
Clostridium sensu stricto	26.2593		-0.6382	-2.2642
Clostridium XI				
Clostridium XII				
Clostridium XlVa				
Clostridium XlVb				
Clostridium XVIII				
Cohnella			-0.7811	
Collinsella				
Comamonas				
Corynebacterium		-0.6451		
Craurococcus			-0.8262	
Cryptanaerobacter			-0.8012	
Cryptomonadaceae				
Cupriavidus			-0.7631	
Curvibacter	13.3254		-0.6667	-1.1884
Daeguia				
Dechloromonas				
Deefgea				
Deinococcus				
Delftia				
Dermabacter				
Dermacoccus				
Desulfitobacterium			-0.7958	
Desulfosporosinus				
Dialister				
Dietzia				
Diplorickettsia				
Dongia				
Dorea				
Duganella				
Dyadobacter				
Elizabethkingia			-0.8086	
Empedobacter				
Emticicia				
Enhydrobacter				
Ensifer				
Enterobacter		-0.6731		
Enterococcus			-0.8396	
Erwinia				
Ethanoligenens				

Exiguobacterium				
Faecalibacterium				
Falsibacillus				
Ferrimicrobium				
Ferruginibacter				
Fervidicella				
Fibrobacter			-0.7867	
Fictibacillus				
Finegoldia				
Flavobacterium	25.7214		-0.8816	-2.1995
Flexivirga				
Fluviicola			-0.7916	
Frischella				
Frondihabitans			-0.6136	
Fructobacillus				
Fulvimonas				
Fusicatenibacter				
Fusobacterium			-0.8080	
Gaiella				
Gallicola				
Gemella			-0.8097	
Gemmata				
Gemmatimonas			-0.7927	
Gemmobacter				
Geobacillus		-0.6457		
Geothermobacter				
Giesbergeria				
Gluconacetobacter				
Gluconobacter				
Gp10				
Gp6				
GpV				
GpXIII				
Gracilibacillus				
Gracilibacter				
Granulicatella			-0.8079	
Granulicella				
Guggenheimella				
Haemophilus				
Hafnia				
Haliangium				
Haliea			-0.8108	
Haliscomenobacter			-0.8184	
Halobacteriovorax				
Halocella			-0.7955	
Hydrogenispora				

Hydrogenophaga			
Hydrogenophilus			
Hymenobacter			
Hyphomicrobium			
Ignatzschineria			
Ilumatobacter			
Intestinimonas			
Isoptericola			
Jahnella			
Janibacter		-0.7954	
Janthinobacterium			
Jeotgalicoccus			
Kineococcus			
Kingella		-0.8151	
Kocuria		-0.8030	
Kofleria			
Komagataeibacter		-0.7957	
Ktedonobacter			
Kytococcus			
Labedella			
Labilithrix			
Laceyella		-0.7938	
Lachnoanaerobaculum			
Lactobacillus			
Lactococcus			
Legionella			
Leifsonia			
Leminorella			
Lentibacillus			
Leucobacter		-0.7731	
Leuconostoc			
Limnohabitans			
Lonsdalea			
Luteibacter			
Luteolibacter			
Lysinibacillus	23.4360	-0.7031	-2.0467
Macellibacteroides			
Magnetospirillum			
Malikia			
Marihabitans			
Marmoricola			
Massilia			
Meiothermus			
Melghiribacillus		-0.8131	
Melghirimyces			
Melissococcus	20.4987	-0.8925	-1.8249

Mesoplasma		-0.6645		
Methanobacterium				
Methylobacillus				
Methylobacterium			-0.7400	
Methylocystis				
Methylophilus				
Methylosinus				
Methylovirgula				
Microbacterium				
Micrococcus				
Micromonospora				
Minicystis				
Modestobacter				
Moheibacter				
Moraxella				
Morganella				
Mucilaginibacter			-0.7850	
Mycobacterium				
Mycoplasma				
Nakamurella				
Neisseria				
Neochlamydia			-0.8082	
Neorhizobium				
Nesterenkonia				
Nguyenibacter				
Niastella				
Nitrosomonas				
Nitrosopumilus				
Nitrospira				
Nitrospirillum				
Nocardioides				
Novosphingobium			-0.7888	
Oceanobacillus	23.5082			-2.1013
Ochrobactrum				
Oligoflexus				
Olsenella				
Opitutus				
Orbus				
Ornithinicoccus				
Ornithinimicrobium				
Ornithobacterium				
Oscillibacter			-0.7509	
Oxobacter				
Paenibacillus	23.8925		-0.8192	-2.0573
Paludibacter				
Pannonibacter			-0.7953	

Pantoea	
Parachlamydia	
Paracoccus	
Patulibacter	
Pedobacter	
Pelagibius	
Pelomonas	-0.8171
Peptoniphilus	-0.7915
Peredibacter	
Petrimonas	
Phaeodactylibacter	
Phenylobacterium	
Phycicoccus	-0.8001
Pilimelia	
Pirellula	
Planctomicrobium	
Planifilum	
Planococcus	
Plesiocystis	
Plesiomonas	
Polaromonas	
Polymorphobacter	
Polynucleobacter	
Pontibacter	
Porphyrobacter	
Porphyromonas	
Portibacter	
Povalibacter	
Prevotella	
Promicromonospora	
Propionibacterium	-0.8042
Prosthecobacter	
Proteus	
Providencia	
Pseudarcicella	
Pseudobacteroides	-0.8079
Pseudochelatococcus	
Pseudoclavibacter	
Pseudolabrys	
Pseudomonas	
Pseudonocardia	
Pseudoxanthomonas	-0.7704
Psychrobacter	
Psychroflexus	
Psychroglaciecola	
Psychrosinus	

Pullulanibacillus		
Ramlibacter		
Raoultella		
Rheinheimera		
Rhizobacter		
Rhizobium	-0.6528	
Rhizomicrobium		-0.7895
Rhodococcus		
Rhodoferax		
Rhodoluna		-0.8268
Rickettsia		
Romboutsia		
Rosenbergiella		
Roseomonas		-0.8381
Rothia		
Rubellimicrobium		
Rubinisphaera		
Ruminococcus		-0.7887
Ruminococcus2		-0.8161
Rummeliibacillus		
Saccharibacillus		-0.8054
Saccharibacter		
Saccharicrinis		
Salinibacillus		
Salinimicrobium		
Salinispira		
Samsonia		
Sandaracinobacter		
Sanguibacter		
Schlegelella		
Schlesneria		
Sedimentibacter		
Sediminibacterium		-0.8096
Segetibacter		
Serratia		
Shewanella		-0.7845
Sideroxydans		
Silvimonas		
Simkania		
Singulisphaera		-0.7621
Skermanella		
Snodgrassella		
Sodalis		
Solitalea		
Sphaerotilus		-0.8220
Sphingobacterium		

Sphingomonas				
Sphingopyxis			-0.7879	
Sphingorhabdus				
Spirillum				
Spiroplasma				
Sporobacter				
Sporolactobacillus			-0.8097	
Sporomusa				
Sporosarcina				
Staphylococcus				
Stenotrophomonas				
Streptococcus				
Streptomyces				
Streptophyta				
Subtercola				
Symbiobacterium		-0.5966	-0.8845	
Syntrophaceticus				
Tahibacter				
Taibaiella				
Tanticharoenia				
Tepidanaerobacter				
Tepidibacillus			-0.7942	
Tepidicella				
Tepidimonas				
Tepidiphilus				
Terribacillus				
Terriglobus				
Terrisporobacter				
Tetrasphaera			-0.8072	
Thermicanus				
Thermoactinomyces			-0.7641	
Thermobacillus			-0.8140	
Thermogutta				
Thermoleophilum				
Thermus				
Thiothrix				
Tissierella	19.3377			-1.6871
Tolumonas				
Tomitella				
Trichococcus				
Tumebacillus				
Turicella			-0.7804	
Turicibacter				
Undibacterium			-0.7740	
Ureibacillus				
Vampirovibrio			-0.8122	

Variovorax			
Veillonella			
Weissella		-0.8005	
Verrucomicrobium			
Verrucosispora			
Williamsia			
Virgibacillus	17.5197		-1.5629
Vogesella			
Xanthomonas			
Yersinia		-0.7373	
Zavarzinella			
Zoogloea			
Zymobacter			

Table S13. Bacterial families based on metabarcoding, shown as for Table S7.

Family	intercept (Estonia)	Finland	Sweden	Read count
Acetobacteraceae				
Acidimicrobiaceae				
Acidothermaceae				
Actinomycetaceae			-0.93	
Aerococcaceae			-0.88	
Aeromonadaceae				
Alcaligenaceae				
Alicyclobacillaceae				
Alteromonadaceae			-0.92	
Anaerolineaceae				
Anaplasmataceae				
Anoxybacter				
Ardenticatenaceae				
Arenicellaceae				
Aridibacter			-0.90	
Armatimonadaceae			-0.91	
Aurantimonadaceae				
Bacillaceae	27.06			-2.35
Bacillaceae 1	34.57		-1.02	-2.96
Bacillaceae 2	20.78		-0.70	-1.84
Bacteriovoracaceae				
Bacteroidaceae				
Bdellovibrionaceae			-0.88	
Beijerinckiaceae				
Beutenbergiaceae				
Bifidobacteriaceae				
Bogoriellaceae				
Bradyrhizobiaceae				
Brevibacteriaceae				
Brevinemataceae				
Brucellaceae				
Bryobacter			-0.87	
Burkholderiaceae				
Campylobacteraceae				
Candidatus Brocadiaceae				
Candidatus Carsonella	16.77		-0.96	-1.49
Candidatus Endomicrobium				
Carnobacteriaceae				
Caulobacteraceae		-0.58		
Cellulomonadaceae			-0.92	
Chitinophagaceae				
Chloroplast				

Christensenellaceae				
Chromatiaceae				
Chryseolinea				
Chthonomonadaceae				
Clostridiaceae	18.14		-0.92	-1.58
Clostridiaceae 1	34.91		-0.86	-2.99
Clostridiaceae 2				
Clostridiaceae 3				
Clostridiaceae 4				
Cohaesibacteraceae				
Comamonadaceae			-0.66	
Coriobacteriaceae				
Corynebacteriaceae		-0.65	-0.89	
Coxiellaceae				
Cryomorphaceae			-0.95	
Cryptosporangiaceae				
Cyclobacteriaceae				
Cystobacteraceae			-0.86	
Cytophagaceae				
Defluviitaleaceae				
Dehalococcoidaceae			-0.92	
Deinococcaceae				
Dermabacteraceae			-0.85	
Dermacoccaceae			-0.89	
Dermatophilaceae				
Desulfobacteraceae				
Desulfobulbaceae			-0.90	
Dietziaceae				
Dissulfuribacter				
Ectothiorhodospiraceae				
Edaphobacter				
Eilatimonas				
Elusimicrobiaceae			-0.89	
Enterobacteriaceae				
Enterococcaceae	24.69		-1.07	-2.18
Entomoplasmataceae		-0.72		
Erysipelotrichaceae				
Erythrobacteraceae				
Fibrobacteraceae			-0.88	
Flavobacteriaceae	31.85		-1.01	-2.70
Fusobacteriaceae			-0.91	
Gaiellaceae				
Gallionellaceae				
Geminicoccus				
Gemmatimonadaceae			-0.89	
Geobacteraceae				

Geodermatophilaceae			-0.86	
Gracilibacteraceae			-0.85	
Granulicella				
Halanaerobiaceae			-0.95	
Haliangiaceae				
Halomonadaceae				
Haloplasmataceae				
Heliobacteriaceae				
Hydrogenophilaceae				
Hyphomicrobiaceae			-0.85	
Hyphomonadaceae				
Iamiaceae			-0.88	
Intrasporangiaceae			-1.01	
Kineosporiaceae				
Kofleriaceae				
Ktedonobacteraceae				
Labilitrichaceae				
Lachnospiraceae	30.68		-0.71	-2.66
Lactobacillaceae				
Legionellaceae				
Leuconostocaceae				
Listeriaceae				
Marinilabiliaceae				
Methanobacteriaceae				
Methylobacteriaceae				
Methylocystaceae				
Methylophilaceae				
Microbacteriaceae		-0.55	-0.94	
Micrococcaceae			-0.88	
Micromonosporaceae				
Mooreiaceae				
Moraxellaceae				
Mycobacteriaceae				
Mycoplasmataceae				
Nakamurellaceae				
Nannocystaceae				
Natranaerovirga	28.41			-2.35
Nautiliaceae			-0.90	
Neisseriaceae		-0.73		
Nitrosomonadaceae				
Nitrosopumilaceae				
Nitrospiraceae				
Nocardiaceae				
Nocardioidaceae			-0.86	
Nocardiopsaceae				
Oligoflexaceae				

Opitutaceae				
Orbaceae				
Oxalobacteraceae				
Paenibacillaceae	24.28		-1.22	-2.09
Paenibacillaceae 1	31.10		-1.05	-2.66
Paenibacillaceae 2			-0.93	
Parachlamydiaceae				
Pasteurellaceae				
Patulibacteraceae				
Peptococcaceae				
Peptococcaceae 1				
Peptoniphilaceae			-0.88	
Peptostreptococcaceae	26.53		-0.87	-2.29
Phyllobacteriaceae				
Piscirickettsiaceae			-0.90	
Planctomycetaceae				
Planococcaceae	32.76		-0.79	-2.80
Polyangiaceae			-0.89	
Porphyromonadaceae				
Prevotellaceae			-0.88	
Prolixibacteraceae				
Promicromonosporaceae				
Propionibacteriaceae			-0.90	
Pseudomonadaceae				
Pseudonocardiaceae			-0.87	
Puniceicoccaceae				
Rhizobiaceae		-0.60		
Rhizomicrobium			-0.92	
Rhodobacteraceae				
Rhodobiaceae				
Rhodocyclaceae				
Rhodospirillaceae				
Rickettsiaceae				
Rikenellaceae				
Ruminococcaceae	16.24		-0.79	-1.47
Sanguibacteraceae				
Saprospiraceae			-1.02	
Schleiferiaceae				
Shewanellaceae			-0.91	
Simkaniaceae				
Sinobacteraceae				
Sphaerobacteraceae				
Sphingobacteriaceae				
Sphingomonadaceae	16.64			-1.45
Spirillaceae				
Spirochaetaceae				

Spiroplasmataceae	
Sporolactobacillaceae	-0.94
Staphylococcaceae	
Streptococcaceae	
Streptomycetaceae	
Succinivibrionaceae	
Synergistaceae	
Syntrophaceae	
Syntrophobacteraceae	-0.86
Terriglobus	
Thermaceae	
Thermoactinomycetaceae	
Thermoactinomycetaceae 1	-0.93
Thermoactinomycetaceae 2	
Thermoanaerobacteraceae	
Thermoleophilaceae	
Thiotrichaceae	
Trueperaceae	
Umboniibacter	
Vallitalea	
Veillonellaceae	
Verrucomicrobiaceae	
Vulgatibacteraceae	
Xanthobacteraceae	
Xanthomonadaceae	

Table S14. Bacterial genera based on metagenomics, shown as for Table S7.

Genus	intercept (Estonia)	Finlan	Swed	Read count
Acaryochloris	-84.52			5.50
Acetoanaerobium	-84.24			5.52
Acetobacter	-32.99		1.23	2.26
Acetobacterium	-74.84		1.21	4.94
Acetohalobium	-48.64			3.19
Acetomicrobium	-29.73			
Acholeplasma	-74.18	0.90	1.74	4.95
Achromobacter	-65.54		1.33	4.32
Acidaminococcus	-102.74			6.75
Acidibrevibacterium	-80.19			5.31
Acidiferrobacter	-31.30			1.98
Acidihalobacter	-57.50			3.77
Acidiphilium	-86.51		1.18	5.68
Acidipropionibacterium	-83.85			5.49
Acidisarcina	-99.14		1.25	6.56
Acidithiobacillus	-113.08	0.81	1.74	7.48
Acidobacterium	-29.51			
Acidovorax	-74.25			4.91
Acinetobacter				
Actinoalloteichus	-103.61		1.90	6.84
Actinobacillus	-68.79		1.27	4.59
Actinomadura	-21.88			1.53
Actinomyces	-52.09	0.86	1.32	3.57
Actinoplanes	-91.34	0.83	1.80	6.07
Actinopolymorpha	-86.00			5.59
Actinopolyspora	-84.60			5.50
Actinosynnema	-76.28			5.04
Adlercreutzia	-29.43			
Advenella	-102.19		1.47	6.75
Aequorivita	-103.21			6.78
Aeribacillus	-75.05			4.92
Aerococcus	-83.78	0.88	1.57	5.61
Aeromicrobium	-91.07		1.69	6.02
Aeromonas	-41.29		1.30	2.78
Afipia	-90.94			5.98
Agarilytica	-84.22			5.52
Agarivorans	-102.89		1.46	6.79
Aggregatibacter	-76.18		1.15	5.08
Agrobacterium	-46.58	0.81	1.51	3.13
Agrococcus	-89.17			5.86
Agromyces	-111.85		1.61	7.37
Ahniella	-99.08			6.49

Akkermansia	-96.94		1.77	6.41
Alcaligenes	-115.62		1.72	7.67
Alcanivorax	-94.86	0.92	1.84	6.32
Algibacter	-87.22			5.74
Algoriphagus	-59.28			3.88
Alicyclobacillus	-84.26			5.55
Aliiarcobacter	-84.55			5.50
Aliivibrio	-62.80	0.79	1.74	4.18
Alistipes	-72.67		1.15	4.83
Alkaliphilus	-64.90	0.71	1.33	4.34
Alkalitalea	-73.92			4.90
Alloactinosynnema	-29.70			
Allofrancisella	-65.98		1.07	4.35
Allokutzneria	-30.97			1.96
Altererythrobacter	-83.15		1.64	5.51
Alteromonas	-74.71	0.93	1.59	5.01
Aminipila	-48.96			3.18
Aminobacter	-48.55			3.22
Amphibacillus	-76.46			5.01
Amycolatopsis	-81.28	0.89	1.67	5.45
Anabaena	-79.84	0.93	1.93	5.30
Anabaenopsis	-85.15			5.54
Anaerobutyricum	-84.70			5.51
Anaerococcus	-97.99			6.42
Anaerolinea				
Anaeromyxobacter	-69.21			4.53
Anaeroplasma				
Anaerostipes	-91.87		1.46	6.08
Anaerotignum	-43.78			2.85
Anaplasma	-41.11			2.70
Anderseniella	-84.71			5.51
Aneurinibacillus	-106.42			7.02
Anoxybacillus	-59.16			3.95
Anoxybacter	-88.31			5.81
Anseongella	-85.32			5.59
Antarcticibacterium	-104.07		1.18	6.86
Antarctobacter	-84.68			5.51
Apibacter	-116.58		1.32	7.70
Aquabacterium	-77.83			5.08
Aquaspirillum	-66.02			4.35
Aquibacillus	-83.26			5.51
Aquicella	-84.89			5.52
Aquifex	-30.46			1.93
Aquiflexum	-59.25			3.88
Aquimarina	-80.19		1.20	5.31
Aquisphaera	-101.47		1.34	6.68

Aquitalea	-97.60		1.43	6.43
Arabia	-30.64			1.93
Arachidicoccus	-73.32	1.01	1.77	4.88
Archangium	-84.81			5.58
Arcobacter				
Arcticibacterium	-63.48		1.22	4.20
Arenibacter	-81.27			5.32
Arenimonas	-85.44			5.60
Arsenicicoccus	-22.04			
Arsenophonus	-27.02			1.88
Arthrobacter	-39.65		1.13	2.68
Asaia				
Asticcacaulis	-82.32			5.41
Atlantibacter	-72.04		1.20	4.76
Aureimonas	-97.22		1.27	6.39
Auricoccus	-68.25			4.49
Avibacterium	-115.73		1.54	7.68
Azoarcus	-115.97	1.06	2.18	7.71
Azorhizobium	-26.91			1.71
Azospira				
Azospirillum	-80.46	1.19	1.98	5.39
Azotobacter	-94.80			6.22
Bacillus	-25.68	1.00	1.46	1.84
Bacterioplanes	-86.61			5.67
Bacteriovorax	-102.87			6.76
Bacteroides	-65.72	1.01	1.63	4.42
Baekduia	-87.61			5.73
Barnesiella	-29.58			
Bartonella				
Basfia	-97.84		1.45	6.44
Basilea	-55.04			3.60
Bdellovibrio	-86.50		1.40	5.77
Beggiatoa	-100.50		1.32	6.61
Beijerinckia	-84.63			5.50
Belliella	-71.82		1.14	4.71
Bernardetia	-70.68	0.96	1.90	4.70
Beutenbergia	-87.65			5.73
Bibersteinia	-97.08		1.42	6.39
Bifidobacterium	-52.01	1.29	1.97	3.54
Blastochloris	-97.97			6.42
Blastomonas	-85.05			5.53
Blattabacterium	-79.31		1.40	5.24
Blautia	-78.53	0.89	1.72	5.23
Bombella	-60.16			3.95
Bordetella	-65.87	0.83	1.50	4.40
Borrelia	-49.76		1.11	3.35

Borreliella	-72.84	0.78	1.36	4.86
Bosea	-93.72		1.60	6.19
Boseongicola	-89.04			5.85
Brachybacterium	-102.70		1.45	6.78
Brachyspira	-73.31	0.72	1.42	4.88
Bradymonas	-83.91			5.46
Bradyrhizobium	-66.53	1.09	2.10	4.45
Brenneria	-59.88	0.82	1.09	4.02
Breoghania				
Brevibacillus	-82.62	0.92	1.71	5.53
Brevibacterium	-81.20		1.10	5.38
Brevundimonas			0.94	
Brochothrix	-47.62	0.80	1.35	3.18
Brucella	-103.90		1.34	6.85
Buchnera	-61.08	1.07	1.61	4.13
Burkholderia	-25.93	1.00	1.46	1.86
Buttiauxella	-68.68		1.00	4.55
Butyricimonas	-85.17			5.58
Butyrivibrio	-79.93			5.29
Bythopirellula	-88.43			5.82
Caldanaerobacter	-99.94			6.55
Caldicellulosiruptor	-92.37	0.89	1.52	6.16
Calditerrivibrio				
Caloramator	-87.51			5.72
Calothrix	-56.68	0.98	1.29	3.87
Caminibacter	-85.69			5.57
Campylobacter	-53.91	1.04	1.28	3.69
Candidatus Actinomarina	-84.87			5.52
Candidatus Amoebophilus	-73.52			4.80
Candidatus Annandia	-84.05			5.47
Candidatus Aquiluna	-83.96			5.46
Candidatus Arthromitus	-71.70	1.00	2.03	4.77
Candidatus Azobacteroides	-85.59			5.61
Candidatus Babela	-84.91			5.56
Candidatus Bipolaricaulis	-58.13			3.79
Candidatus Blochmannia	-74.39		1.56	4.91
Candidatus Cardinium	-111.88		1.81	7.41
Candidatus Carsonella				
Candidatus Cytomitobacter	-86.57			5.66
Candidatus Deianiraea	-83.41			5.42
Candidatus Desulfofervidus	-84.94			5.56
Candidatus Desulforudis				
Candidatus Doolittlea	-96.63		1.45	6.36
Candidatus Endolissoclinum	-83.49			5.47
Candidatus Evansia	-85.52			5.56
Candidatus Fokinia	-83.04			5.40

Candidatus Fonsibacter				
Candidatus Fukatsuia	-115.91	0.83	1.78	7.66
Candidatus Gullanella	-104.27		1.19	6.85
Candidatus Hamiltonella	-78.06		1.71	5.16
Candidatus Hepatoplasma	-84.07			5.47
Candidatus Hoaglandella	-102.90			6.76
Candidatus Ishikawaella	-103.87		1.17	6.84
Candidatus Izimaplasma	-84.99			5.57
Candidatus Kinetoplastibacterium	-44.74			2.97
Candidatus Kuenenia	-84.92			5.56
Candidatus Methylopumilus	-79.15		1.41	5.26
Candidatus Midichloria	-85.58			5.57
Candidatus Mikella	-87.49			5.72
Candidatus Moanabacter	-78.74			5.17
Candidatus Moranella	-87.03			5.69
Candidatus Nanopelagicus	-83.67			5.48
Candidatus Nardonella	-85.71			5.57
Candidatus Nitrosoglobus	-84.51			5.50
Candidatus Nitrotoga	-112.82		1.64	7.44
Candidatus Paracaedibacter	-85.78			5.58
Candidatus Paracaedimonas	-25.07			
Candidatus Pelagibacter	-73.97	0.73	1.28	4.92
Candidatus Peribacter	-85.61			5.57
Candidatus Phaeomarinobacter	-80.55			5.28
Candidatus Phytoplasma	-91.93		1.51	6.06
Candidatus Planktophila	-95.98			6.33
Candidatus Portiera	-85.01			5.53
Candidatus Profftella	-84.70			5.51
Candidatus Promineofilum	-56.20			3.66
Candidatus Protochlamydia	-63.87			4.21
Candidatus Puniceispirillum	-85.67			5.57
Candidatus Regiella	-98.60		1.46	6.49
Candidatus Riesia	-84.31			5.55
Candidatus Ruthia	-44.20			2.86
Candidatus Sneabacter	-30.87			1.95
Candidatus Solibacter	-65.74			4.33
Candidatus Sulcia	-64.19			4.18
Candidatus Sumerlaea	-29.74			
Candidatus Symbiobacter	-31.23			1.97
Candidatus Tachikawaea	-85.73			5.61
Candidatus Tenderia	-84.46			5.49
Candidatus Thioglobus	-69.16		1.13	4.56
Candidatus Tokpelaia	-99.08			6.49
Candidatus Tremblaya	-84.25			5.48
Candidatus Westeberhardia	-86.94			5.69
Candidatus Xiphinematobacter	-22.44			

Capnocytophaga	-67.69	0.94	1.48	4.55
Caproiciproducens	-74.80			4.88
Carboxydocella	-85.92			5.59
Carboxydothermus	-84.86			5.52
Cardiobacterium	-99.74			6.53
Carnobacterium				
Castellaniella	-83.79			5.45
Catenovulum	-59.02			3.91
Catenulispora	-105.09		1.20	6.90
Caulobacter	-90.56	0.82	2.08	5.99
Cedecea	-42.66	0.94	1.85	2.89
Celeribacter	-104.35		1.30	6.91
Cellulomonas	-101.71		1.31	6.72
Cellulophaga	-70.45		1.15	4.68
Cellulosilyticum	-93.35		1.74	6.20
Cellulosimicrobium	-84.12			5.51
Cellvibrio	-106.19		1.83	7.01
Cetia	-85.22			5.54
Chamaesiphon	-102.81			6.75
Changchengzhania	-69.88		1.02	4.60
Chania	-68.80		0.98	4.57
Chelativorans	-90.70		1.49	5.98
Chelatococcus	-101.48			6.68
Chitinolyticbacter	-84.14			5.47
Chitinophaga	-63.99	0.81	1.43	4.29
Chlamydia	-70.53	0.79	1.52	4.72
Chloracidobacterium	-85.35			5.55
Chlorobium	-59.77			3.95
Chloroflexus	-86.64			5.67
Chloroherpeton	-85.05			5.53
Chondrocystis	-87.43			5.72
Chondromyces	-86.56			5.66
Christensenella	-89.95			5.91
Chromobacterium	-98.67	0.85	1.83	6.56
Chryseobacterium	-62.75	1.08	1.76	4.26
Chryseolinea	-86.64			5.67
Citricoccus	-84.87			5.52
Citrobacter	-46.81	0.89	1.55	3.20
Clavibacter	-29.37			1.93
Cloacibacillus	-84.89			5.52
Clostridioides	-55.26	0.90	1.18	3.71
Clostridium	-55.39	1.11	1.48	3.81
Cnuibacter				
Cobetia	-85.92			5.59
Cohaesibacter	-103.58		1.17	6.83
Cohnella	-66.38			4.38

Collimonas	-95.38		1.74	6.32
Collinsella	-83.81			5.45
Colwellia	-76.87	0.72	1.63	5.14
Comamonas	-117.17		1.63	7.75
Commensalibacter				
Conexibacter	-84.37			5.48
Coprococcus	-98.65			6.46
Coprothermobacter	-85.27			5.59
Corallococcus	-102.28		1.35	6.73
Corynebacterium	-65.47	1.02	1.78	4.40
Coxiella	-85.45			5.59
Crassaminicella	-111.37		1.76	7.35
Crinalium	-84.51			5.54
Croceibacter	-27.12			
Croceicoccus	-103.21		1.34	6.81
Crocosphaera	-86.45			5.65
Cronobacter	-61.72	0.87	1.75	4.16
Cryobacterium	-104.69		1.18	6.90
Cupriavidus	-70.18	1.12	1.83	4.70
Curtobacterium	-33.22		1.36	2.19
Curvibacter	-105.52		1.92	6.97
Cyanobacterium	-80.17		1.11	5.30
Cyanobium	-87.45			5.72
Cyanothece	-99.62			6.53
Cyclobacterium	-61.99		1.03	4.12
Cycloclasticus	-67.71			4.46
Cylindrospermum	-115.36	0.81	1.77	7.63
Cystobacter	-71.31	0.76	1.57	4.72
Cytophaga	-75.62			4.96
Dactylococcopsis	-85.41			5.56
Dechloromonas	-101.84		1.34	6.70
Defluviitoga	-84.86			5.52
Dehalobacter	-85.11			5.54
Dehalobacterium	-77.33			5.10
Dehalococcoides	-70.96			4.64
Dehalogenimonas				
Deinococcus	-57.80		0.95	3.92
Delftia	-99.21		1.48	6.53
Denitratisoma	-27.21			
Denitrovibrio	-80.21			5.25
Dermabacter				
Desulfallas	-84.42			5.53
Desulfatibacillum	-85.33			5.58
Desulfitobacterium	-31.39			2.08
Desulfobacterium	-102.78		1.48	6.78
Desulfobacula	-78.09			5.13

Desulfobulbus	-86.40			5.67
Desulfocapsa	-84.36			5.49
Desulfococcus	-96.67			6.36
Desulfofarcimen	-51.62			3.38
Desulfofundulus	-84.20			5.51
Desulfoglaeba	-29.43			
Desulfomicrobium	-52.87			3.49
Desulfomonile	-84.95			5.52
Desulfosporosinus	-91.23	0.87	1.64	6.08
Desulfotalea	-107.66		1.20	7.10
Desulfotomaculum	-103.22		1.31	6.82
Desulfovibrio	-54.81		1.07	3.66
Desulfurivibrio	-28.19			1.80
Desulfurobacterium	-52.84			3.44
Desulfuromonas	-98.75			6.47
Devosia	-70.52	0.79	1.40	4.68
Dialister	-98.36		1.31	6.47
Dichelobacter	-84.97			5.52
Dickeya	-45.13	0.77	1.56	3.06
Dictyoglomus	-67.14			4.42
Dietzia	-92.73	0.93	1.93	6.17
Dokdonella	-30.36			
Dokdonia	-76.60			5.05
Dolichospermum	-101.16		1.35	6.65
Dolosigranulum	-30.88			1.95
Draconibacterium	-83.83			5.45
Duncaniella	-29.06			1.84
Dyadobacter	-107.14	0.92	2.02	7.09
Dyella	-93.49		1.51	6.16
Echinicola	-37.05		1.26	2.46
Ectothiorhodospira	-27.11			
Edwardsiella	-50.95			3.44
Eggerthella	-111.92		1.62	7.38
Egibacter	-29.39			
Egicoccus	-83.15			5.40
Ehrlichia	-75.94		1.00	5.06
Eikenella	-90.76			5.99
Elizabethkingia	-68.16		1.24	4.55
Elusimicrobium	-23.97			1.53
Emcibacter	-77.49			5.10
Emticicia	-83.02			5.40
Endomicrobium	-99.27		1.43	6.55
Endozoicomonas	-77.91		1.07	5.11
Ensifer	-71.94	0.75	1.43	4.77
Enterobacter				
Enterococcus	-59.28	0.95	1.45	4.06

Enterovibrio	-106.18				7.00
Entomoplasma	-49.96	0.69	1.36		3.36
Epibacterium	-105.33		1.17		6.94
Ereboglobus	-25.97				
Erwinia					
Erysipelothrix	-70.14		1.10		4.64
Erythrobacter	-68.13	0.77	1.66		4.51
Escherichia	-52.58	1.21	1.94		3.57
Eubacterium	-72.92		1.50		4.82
Euhalothece	-86.61				5.66
Euzebya	-84.40				5.49
Euzebyella	-98.57				6.45
Exiguobacterium	-72.01				4.79
Ezakiella	-85.84				5.58
Fabibacter	-89.75				5.90
Faecalibacterium	-79.62				5.22
Faecalitalea	-87.25				5.71
Fermentimonas	-99.69				6.53
Ferrimonas					
Ferriphaselus	-84.59				5.50
Ferrovibrio	-89.38				5.88
Fervidobacterium	-116.59		1.30		7.70
Fibrella	-67.38		1.65		4.46
Fibrobacter					
Fictibacillus	-49.76				3.28
Filifactor					
Filimonas	-100.90		1.34		6.64
Filomicrobium	-87.13				5.70
Fimbriimonas	-72.52				4.73
Finegoldia	-59.79				3.92
Fischerella	-103.29		1.71		6.84
Flagellimonas	-84.79				5.55
Flammeovirga	-70.29		1.18		4.66
Flaviflexus	-84.56				5.50
Flavisolibacter	-87.36		1.17		5.79
Flavivirga	-80.27				5.27
Flavobacterium	-93.55				6.14
Flexistipes	-84.79				5.51
Fluviicola	-94.13		1.35		6.21
Formosa	-93.40	0.86	1.70		6.21
Francisella	-64.58	1.09	1.61		4.35
Frankia					
Frateuria	-85.21				5.54
Friedmanniella	-98.98				6.48
Frischella	-27.14		1.15		1.87
Frondihabitans	-74.00	0.74	1.66		4.89

Fructobacillus	-30.41			1.93
Fuerstia	-84.76			5.51
Fusobacterium	-48.94	0.75	1.24	3.35
Gallaecimonas	-103.84			6.84
Gallibacterium	-111.16		1.75	7.34
Gallionella	-81.71			5.35
Gardnerella				
Geitlerinema	-84.94			5.52
Gemella	-56.28		1.15	3.75
Geminocystis	-113.95	0.93	2.01	7.54
Gemmata	-81.39			5.36
Gemmatimonas	-82.11			5.39
Gemmatirosa	-99.84			6.54
Geobacillus	-82.30		1.36	5.45
Geobacter	-78.67	0.97	1.66	5.28
Georgenia	-107.15		1.21	7.08
Geosporobacter	-66.27			4.35
Gibbsiella	-87.80		1.31	5.84
Gilliamella	-35.81	0.69	1.31	2.45
Gillisia	-86.23			5.64
Glaciecola	-48.82		1.03	3.23
Glaesserella	-81.45		1.34	5.40
Gloeobacter	-83.39		1.40	5.50
Gloeocapsa	-99.26			6.50
Gloeothece	-38.66			2.63
Gluconacetobacter	-100.04		1.82	6.62
Gluconobacter				
Glutamicibacter	-116.92		1.31	7.73
Glycocaulis	-27.04			
Gordonia	-92.88		1.45	6.14
Gordonibacter	-100.65			6.59
Gottschalkia	-73.26		1.43	4.83
Gramella	-79.00		1.29	5.24
Granulibacter	-82.22		1.52	5.44
Granulicella	-24.78		0.86	1.60
Granulosicoccus	-89.15			5.86
Grimontia	-108.82			7.19
Gryllotalpicola				
Gynuella				
Haematobacter	-83.69			5.44
Haematospirillum	-26.20			
Haemophilus	-54.09	0.88	1.46	3.65
Hafnia	-40.55	0.67	1.13	2.74
Hahella	-70.25			4.66
Halanaerobium	-95.58		1.25	6.31
Haliangium	-84.74			5.55

Halioglobus	-80.77		1.10	5.30
Haliscomenobacter	-80.53		1.09	5.29
Halobacillus	-42.81			2.89
Halobacteriovorax	-92.58		1.35	6.11
Halobacteroides	-101.76			6.70
Halocella	-106.30			7.01
Halocynthiibacter	-82.76			5.42
Halomonas	-67.75	0.94	1.64	4.55
Halorhodospira	-86.37			5.68
Halotalea	-88.22			5.79
Halothermothrix	-83.94			5.46
Halothiobacillus	-87.83			5.75
Hartmannibacter	-84.73			5.51
Hathewaya	-59.90			3.92
Helicobacter	-60.33	1.02	1.46	4.10
Herbaspirillum				
Herbinix	-98.79			6.47
Herminiimonas	-104.93		1.43	6.95
Herpetosiphon	-35.12			2.33
Hirschia	-99.10			6.52
Histophilus	-118.67		1.50	7.85
Hoeflea	-67.71			4.45
Hoyosella	-85.69			5.57
Humibacter	-84.70			5.51
Hungateiclostridium	-70.62	0.80	1.39	4.73
Hungatella	-84.97			5.57
Hydrogenobaculum	-84.83			5.58
Hydrogenophaga				
Hydrogenophilus	-26.87			
Hydrogenovibrio	-117.07		1.34	7.73
Hydromonas	-85.69			5.61
Hylemonella	-27.89			1.78
Hymenobacter	-78.64	0.86	1.69	5.24
Hyphomicrobium	-101.17		1.34	6.66
Hyphomonas	-69.34			4.57
Idiomarina	-102.29		1.57	6.78
Ignatzschineria	-26.95			
Ignavibacterium	-98.37		1.31	6.47
Ilyobacter	-77.98			5.09
Immundisolibacter	-26.70			
Indioceanicola	-83.27			5.46
Inhella	-84.09			5.51
Intrasporangium	-84.99			5.53
Iodobacter	-97.40			6.40
Isoptericola	-21.34			1.37
Isosphaera	-85.20			5.54

Izhakiella	-72.17		1.20	4.77
Janibacter				1.14
Jannaschia	-86.13			5.60
Janthinobacterium	-107.15	0.92	2.02	7.10
Jatrophihabitans	-26.98			
Jeotgalibaca	-63.07	0.74	1.32	4.21
Jeotgalibacillus	-84.25			5.52
Jiangella	-62.31			4.14
Jonesia	-85.68			5.57
Kangiella	-72.16		1.29	4.77
Ketobacter	-25.84			1.68
Ketogulonicigenium	-80.33			5.27
Kibdelosporangium				
Kineococcus	-98.95			6.48
Kingella	-98.66			6.49
Kitasatospora	-85.74			5.69
Klebsiella				
Kluyvera	-61.11		1.02	4.08
Kocuria	-85.15			5.58
Komagataeibacter				
Kordia	-116.98		1.62	7.74
Kosakonia	-63.68	0.80	1.40	4.27
Kosmotoga	-84.82			5.56
Kozakia	-61.81	0.76	1.08	4.13
Kribbella				
Kurthia	-65.75			4.38
Kushneria	-73.97			4.91
Kutzneria	-38.84			2.57
Kyrpidia	-83.72			5.44
Labilithrix	-108.40			7.17
Labrenzia	-87.07	0.87	1.39	5.78
Labrys	-39.84			2.62
Lachnoanaerobaculum	-83.20		1.47	5.49
Lachnoclostridium	-70.38	0.87	1.52	4.72
Lacimicrobium	-101.73		1.36	6.69
Lacinutrix	-95.69		1.44	6.30
Lactobacillus				
Lactococcus				
Lacunisphaera	-79.22			5.18
Laribacter	-84.90			5.52
Lautropia				
Lawsonella	-30.27			1.92
Lawsonia	-74.83			4.91
Leadbetterella	-85.26			5.54
Leclercia	-55.72		1.22	3.71
Legionella	-66.40	1.28	1.74	4.50

Leifsonia	-79.09			5.23
Leisingera	-96.30		1.30	6.33
Lelliottia				
Leminorella	-111.37		1.75	7.35
Lentibacillus	-99.87			6.54
Lentzea	-89.88			5.91
Leptolyngbya	-104.62		1.34	6.90
Leptospira	-55.58		1.06	3.77
Leptospirillum	-100.54			6.59
Leptothrix	-77.68			5.08
Leptotrichia	-68.80	0.95	1.82	4.62
Leuconostoc				
Liberibacter	-94.72		1.24	6.29
Limnobaculum	-110.85	0.85	1.86	7.33
Limnohabitans				
Listeria	-54.92	0.84	1.47	3.71
Litoreibacter	-84.34			5.52
Litorilituus	-73.22		1.06	4.82
Lonsdalea	-80.41		1.24	5.34
Luteibacter	-38.91		1.12	2.57
Luteimonas	-105.48		1.17	6.97
Luteipulveratus	-85.38			5.59
Luteitalea	-100.39			6.61
Lutibacter	-61.04			4.04
Lysinibacillus	-57.80	1.07	1.57	3.95
Lysobacter	-74.65		1.33	4.96
Macrococcus	-79.28	0.97	1.90	5.27
Magnetococcus	-84.16			5.51
Magnetospira	-98.87			6.47
Magnetospirillum	-74.59		1.15	4.93
Mahella	-85.88			5.59
Mannheimia	-61.21		1.58	4.04
Maribacter	-85.32	0.99	1.79	5.70
Marichromatium	-102.06			6.72
Mariniblastus	-65.70		1.28	4.35
Mariniflexile	-67.37			4.40
Marinilactibacillus	-46.45			3.07
Marinitoga	-44.26			2.87
Marinobacter	-61.47	0.83	1.42	4.14
Marinobacterium	-86.42			5.65
Marinomonas	-68.79	0.70	1.25	4.60
Marinovum	-79.58		1.08	5.22
Mariprofundus	-36.35			2.42
Maritalea	-83.56			5.43
Marivirga	-49.64			3.24
Martelella	-99.71		1.37	6.60

Massilia	-68.83			4.59
Megamonas	-78.45		1.21	5.19
Megasphaera				
Melaminivora	-112.17		1.62	7.39
Melioribacter	-29.76			1.89
Melissococcus				
Melittangium	-71.32		1.15	4.68
Mesoplasma	-53.38	0.93	1.64	3.61
Mesorhizobium	-80.72	1.19	2.00	5.41
Mesotoga	-31.18			1.99
Metakosakonia	-87.59			5.73
Methylacidiphilum	-98.91			6.52
Methylibium	-85.29			5.55
Methylobacterium	-90.40	0.80	1.80	6.01
Methylocaldum	-84.70			5.51
Methyloceanibacter	-83.58			5.43
Methylocella	-109.44		1.20	7.24
Methylococcus				
Methylocystis	-102.37			6.72
Methylomicrobium	-66.79		1.02	4.42
Methylomonas	-64.56		1.38	4.27
Methylomusa	-85.48			5.56
Methylophaga	-72.83			4.81
Methylophilus	-97.13		1.45	6.39
Methylorubrum	-87.15		1.24	5.76
Methylosinus	-99.42			6.51
Methylotenera	-58.72			3.89
Methylovirgula	-90.82			5.96
Methylovorus	-84.09			5.47
Methylovulum	-85.38			5.55
Micavibrio	-80.91			5.30
Microbacterium	-110.33	0.87	2.15	7.32
Microbulbifer	-72.47		1.51	4.82
Microchaete	-105.83		1.51	7.00
Microcoleus	-94.88			6.23
Microcystis	-60.58		1.00	4.03
Microlunatus	-104.09		1.32	6.88
Micromonospora	-61.75	0.75	1.41	4.14
Micropruina				
Microterricola	-28.27			1.81
Microvirga	-101.50		1.35	6.68
Microvirgula	-84.43			5.49
Minicystis	-104.77		1.34	6.91
Miniimonas				
Mitsuaria	-84.27			5.52
Mixta	-49.96		0.91	3.33

Modestobacter	-98.08			6.42
Moorea	-76.68		1.71	5.07
Moorella	-31.02			1.98
Moraxella	-76.55		1.38	5.08
Mordavella	-85.39			5.55
Morganella	-75.01			5.00
Moritella	-80.40		1.41	5.34
Mucilaginibacter	-76.88	1.08	1.95	5.14
Mucinivorans	-75.67			4.94
Muribaculum	-76.63		1.19	5.03
Muricauda	-84.77			5.51
Muriicola				
Mycetocola	-48.47			3.20
Mycoavidus	-88.62			5.83
Mycobacterium	-54.12	1.19	1.79	3.64
Mycobacteroides	-103.04		1.77	6.84
Mycolicibacter	-89.21			5.87
Mycolicibacterium	-60.82	1.01	1.87	4.09
Mycoplasma				
Myroides	-80.65		1.66	5.33
Myxococcus	-68.71		1.02	4.56
Nakamurella	-106.62			7.03
Natranaerobius	-85.49			5.56
Nautilia	-62.14		1.00	4.10
Neisseria	-81.20	0.99	1.85	5.44
Neoasaia	-77.98		1.19	5.16
Neochlamydia	-53.79			3.52
Neokomagataea	-55.95		1.03	3.77
Neorhizobium	-65.64		1.48	4.35
Nesterenkonia	-84.18			5.47
Niabella	-88.91			5.86
Niastella	-87.23			5.71
Nibricoccus	-85.90			5.63
Nissabacter	-63.47		1.23	4.19
Nitratifractor	-85.10			5.53
Nitratireductor	-109.18		1.19	7.22
Nitrobacter	-87.03			5.69
Nitrosococcus	-89.36		1.95	5.90
Nitrosomonas	-84.44	0.83	1.77	5.60
Nitrosospira	-101.81		1.17	6.69
Nitrospira	-63.43		1.23	4.20
Nitrospirillum				
Niveispirillum	-81.21			5.35
Nocardia	-46.59	0.76	1.32	3.13
Nocardioides	-98.33		1.70	6.53
Nocardiopsis	-96.87		1.25	6.40

Nodularia	-79.18			5.22
Nonlabens	-69.17		1.29	4.59
Nonomuraea	-101.25		1.35	6.66
Nostoc	-43.06	0.79	1.20	2.93
Novibacillus	-31.21			1.97
Noviherbaspirillum	-27.26			
Novosphingobium	-83.15	0.92	1.76	5.54
Obesumbacterium	-69.13			4.58
Oblitimonas	-66.53			4.41
Oceanicoccus	-88.23			5.77
Oceanicola	-42.06			2.76
Oceanimonas	-86.48			5.66
Oceanisphaera	-65.61		0.99	4.31
Oceanispirochaeta	-85.54			5.60
Oceanobacillus	-59.94			3.98
Ochrobactrum	-89.00	1.00	1.90	5.91
Octadecabacter	-88.91		1.27	5.91
Odoribacter	-77.31			5.08
Oenococcus	-46.00		1.36	3.06
Oerskovia	-85.15			5.54
Oleiphilus	-63.27			4.16
Oleispira	-95.03			6.24
Oligella	-100.08			6.55
Oligotropha	-28.66			1.83
Olivibacter	-85.58			5.57
Olleya	-92.42		1.48	6.10
Olsenella	-73.67			4.81
Opitutus	-83.58			5.48
Orientia	-73.84		1.58	4.88
Ornithinimicrobium	-53.82			3.53
Ornithobacterium	-99.18			6.50
Orrella	-84.30			5.48
Oryzomicrobium	-25.24			
Oscillatoria	-108.95		1.22	7.21
Oscillibacter	-100.05			6.55
Ottowia	-84.17			5.47
Owenweeksia	-84.85			5.52
Oxalobacter	-84.12			5.47
Paenalcaligenes	-103.47		1.15	6.82
Paenarthrobacter	-29.18			1.84
Paenibacillus				
Paeniclostridium	-97.48		1.35	6.46
Paenisporosarcina	-57.13			3.79
Paludibacter	-68.31			4.45
Paludisphaera	-30.89			2.06
Panacibacter	-106.76			7.04

Pandoraea	-83.40	0.97	1.76	5.57
Pannonibacter	-99.09			6.53
Pantoea				
Parabacteroides	-102.05	0.93	2.08	6.76
Paraburkholderia	-33.65	1.17	1.77	2.34
Parachlamydia	-98.81			6.47
Paracoccus	-118.77	0.87	1.89	7.88
Parageobacillus	-31.03			1.96
Paraglaciecola	-97.08		1.73	6.43
Paraliobacillus	-111.17		1.61	7.33
Paraphotobacterium	-104.00		1.20	6.83
Paraprevotella	-99.36			6.51
Pararhodospirillum	-27.97			1.79
Parasaccharibacter				
Parashewanella	-41.64		1.23	2.72
Parvimonas	-115.86		1.48	7.65
Pasteurella	-69.33		0.91	4.62
Paucibacter	-78.56			5.13
Paucimonas	-38.61	0.77	1.50	2.59
Pectobacterium	-76.41	1.13	1.83	5.16
Pediococcus				
Pedobacter	-53.55		1.18	3.60
Pelagibaca	-84.29			5.52
Pelagibacterium	-26.71			
Pelobacter	-92.01		1.46	6.11
Pelodictyon	-85.12			5.60
Pelolinea	-84.83			5.52
Pelosinus	-68.44		0.97	4.55
Pelotomaculum	-84.66			5.50
Peptoclostridium	-82.00			5.37
Peptoniphilus	-98.54			6.50
Persephonella	-85.31			5.55
Petrimonas	-86.39			5.65
Petrocella	-87.01			5.69
Petrotoga	-85.29			5.59
Phaeobacter	-88.73		1.17	5.89
Phascolarctobacterium	-85.69			5.57
Phaseolibacter	-26.88			
Phenylobacterium	-101.23		1.32	6.66
Photobacterium	-58.94		0.89	3.94
Photorhabdus	-67.49	0.82	1.49	4.52
Phreatobacter	-73.50		1.36	4.87
Phycicoccus	-99.00			6.48
Phyllobacterium	-68.24		1.28	4.50
Phytobacter	-74.08			4.88
Pigmentiphaga	-95.83		1.26	6.36

Pirellula	-57.32			3.76
Piscirickettsia	-58.05		1.02	3.81
Pistricoccus	-62.59			4.08
Planctomyces	-96.94			6.40
Planctopirus				
Planktomarina	-24.23			
Planktothrix	-103.39		1.34	6.82
Planococcus	-65.69	0.88	1.60	4.41
Planomicrobium	-83.92			5.46
Plantactinospora	-106.56		1.19	7.04
Plantibacter	-81.26		1.30	5.36
Pleomorphomonas	-81.81			5.36
Plesiomonas	-111.84	0.84	1.85	7.40
Pleurocapsa	-87.61			5.73
Pluralibacter	-62.89		1.27	4.17
Polaribacter	-56.29	1.01	1.64	3.77
Polaromonas	-104.44		1.42	6.92
Polymorphum				
Polynucleobacter	-102.09		1.33	6.73
Pontibacter	-73.95		1.47	4.91
Porphyrobacter	-123.05	0.84	1.79	8.16
Porphyromonas	-102.13		1.26	6.76
Pragia	-44.41		1.13	2.94
Prevotella	-80.88	1.07	1.85	5.41
Prochlorococcus	-71.24	0.79	1.40	4.79
Propionibacterium	-86.26			5.67
Prosthecochloris	-60.85			3.99
Proteiniphilum	-81.85			5.36
Proteus			0.81	
Providencia	-48.39	0.85	1.64	3.28
Pseudanabaena	-101.24		1.35	6.66
Pseudarcicella	-85.49			5.56
Pseudarthrobacter	-71.44		1.13	4.75
Pseudescherichia	-97.43		1.83	6.43
Pseudoalteromonas	-70.44	1.32	2.02	4.77
Pseudobacter	-105.71		1.19	6.98
Pseudoclostridium	-65.70			4.31
Pseudodesulfovibrio	-86.93			5.72
Pseudoflavitalea	-106.41			7.02
Pseudoflavonifractor	-29.39	-0.69		
Pseudogulbenkiania	-81.37			5.34
Pseudohongiella	-86.77			5.68
Pseudolabrys	-74.18			4.92
Pseudomonas				
Pseudonocardia	-37.83			2.53
Pseudopedobacter	-85.09			5.57

Pseudorhodobacter	-27.04			
Pseudorhodoplanes	-83.28			5.41
Pseudothermotoga	-75.82			4.99
Pseudovibrio	-63.76			4.20
Pseudoxanthomonas	-97.24		1.09	6.40
Psychrobacter	-84.59	1.03	1.93	5.65
Psychroflexus	-84.88			5.52
Psychromonas	-81.46		1.26	5.39
Psychroserpens	-85.26			5.55
Pusillimonas	-91.86			6.05
Rahnella				
Ralstonia	-67.21	1.02	1.63	4.52
Ramlibacter	-96.98		1.43	6.39
Raoultella				
Raphidiopsis	-85.59			5.61
Rathayibacter	-105.94		1.19	6.99
Reinekea	-31.28			2.02
Renibacterium				
Rheinheimera	-58.01		1.22	3.84
Rhizobacter	-77.27			5.05
Rhizobium	-29.59			2.07
Rhizorhabdus	-29.22			
Rhodanobacter	-51.41			3.38
Rhodobaca	-84.76			5.51
Rhodobacter	-113.04	0.80	1.80	7.50
Rhodococcus	-73.56		1.20	4.89
Rhodoferax	-112.59		1.71	7.46
Rhodoluna				
Rhodopirellula	-26.99			
Rhodoplanes	-86.17			5.64
Rhodopseudomonas	-81.11	0.92	2.11	5.38
Rhodospirillum	-101.32		1.35	6.67
Rhodovulum	-55.32			3.63
Rickettsia				
Rickettsiella	-107.37		1.22	7.06
Riemerella	-105.15		1.36	6.94
Rippkaea	-88.51			5.82
Rivularia	-42.21			2.77
Robiginitalea	-26.79			
Rodentibacter	-110.42		1.75	7.29
Romboutsia				
Roseburia	-88.70		1.08	5.87
Roseibacterium	-29.48			1.86
Roseiflexus	-89.16			5.86
Roseimaritima	-73.37			4.85
Roseitalea	-97.34			6.40

Rosenbergiella	-24.77			
Roseobacter	-96.17			6.33
Roseomonas	-82.80		1.19	5.48
Roseovarius	-98.19			6.43
Rothia	-103.18		1.32	6.82
Ruania	-84.43			5.49
Rubinisphaera	-85.42			5.56
Rubrivivax	-78.07			5.10
Rubrobacter	-85.64			5.60
Ruegeria	-65.86			4.36
Rufibacter	-101.90		1.33	6.71
Rugosibacter	-85.65			5.61
Ruminiclostridium	-84.61			5.54
Ruminococcus	-53.52		0.95	3.57
Rummeliibacillus				
Runella	-89.96	0.94	2.18	5.99
Saccharibacter	-26.37			
Saccharomonospora	-86.26			5.64
Saccharophagus	-87.09			5.70
Saccharospirillum	-27.26			
Saccharothrix	-85.99			5.65
Sagittula	-58.73			3.84
Salegentibacter	-105.81			6.98
Salilacibacter	-88.61			5.82
Salinibacter	-52.12			3.44
Salinibacterium	-86.66			5.67
Salinicoccus	-84.43			5.49
Salinimonas	-81.63		1.33	5.42
Salinisphaera	-99.23			6.50
Salinispora	-101.22			6.66
Salinivibrio	-121.25		1.65	8.04
Salinivirga	-84.49			5.53
Salipiger				
Salmonella	-36.81	0.76	1.24	2.54
Sandaracinus	-78.27			5.11
Sanguibacter	-97.56			6.39
Saprospira				
Schaalia	-75.27			4.97
Scytonema	-97.40		1.17	6.46
Scytonematopsis	-29.01			
Sebaldella	-101.06			6.65
Sedimentisphaera	-98.65			6.46
Sedimentitalea	-84.29			5.48
Sediminicola	-90.49			5.95
Sediminispirochaeta	-85.61			5.57
Segniliparus	-29.24			

Selenomonas	-109.14			7.22
Seonamhaeicola	-84.98			5.53
Serinicoccus	-104.26		1.35	6.88
Serpentinomonas	-85.52			5.56
Serratia				
Shewanella	-74.09	1.21	1.92	5.03
Shigella	-83.83		1.06	5.55
Shimwellia	-80.24		1.29	5.32
Shinella	-99.58			6.52
Siansivirga	-84.67			5.51
Sideroxydans	-85.69			5.57
Silicimonas	-29.84			
Silvanigrella	-116.16	0.80	1.74	7.70
Simiduia	-26.93			
Simonsiella	-55.18			3.60
Singulisphaera	-68.98	0.73	1.54	4.57
Sinomonas	-87.81			5.74
Sinorhizobium	-45.35	0.76	1.82	3.00
Sneathia	-84.58			5.50
Snodgrassella				
Sodalis	-52.11		1.18	3.47
Soehngenia	-85.06			5.53
Solibacillus	-116.46		1.47	7.71
Solitalea	-97.54		1.44	6.42
Sorangium	-63.97		0.93	4.25
Sphaerobacter	-84.92			5.52
Sphaerochaeta	-83.83			5.49
Sphaerospermopsis	-85.24			5.54
Sphaerotilus	-103.80		1.36	6.85
Sphingobacterium	-85.88	1.17	2.08	5.75
Sphingobium	-75.97	1.05	1.60	5.09
Sphingomonas	-39.61		1.27	2.65
Sphingopyxis	-87.67		1.52	5.82
Sphingorhabdus	-84.58		1.34	5.62
Sphingosinicella	-105.09		1.19	6.92
Spiribacter	-80.69			5.28
Spirochaeta	-78.29		1.29	5.19
Spiroplasma				
Spirosoma	-85.15	1.13	1.79	5.70
Spongiibacter	-104.54			6.89
Sporanaerobacter	-105.62			6.97
Sporolactobacillus	-97.35		1.29	6.40
Sporomusa	-87.68			5.76
Sporosarcina	-77.97	0.95	1.78	5.23
Stackebrandtia	-25.16			
Stanieria	-76.68			5.06

Staphylococcus	-62.38	0.98	1.60	4.21
Stappia	-83.19			5.41
Starkeya	-84.38			5.49
Stella	-69.39		1.30	4.59
Stenotrophomonas				
Stigmatella	-79.96			5.26
Streptacidiphilus	-90.20			5.90
Streptobacillus	-87.45			5.72
Streptococcus	-31.56			2.19
Streptomonospora	-87.67			5.76
Streptomyces				
Streptosporangium	-87.22		1.09	5.77
Sulfitobacter	-82.49	0.75	1.39	5.49
Sulfobacillus	-77.69			5.10
Sulfuricaulis	-85.23			5.54
Sulfuricella	-28.71			1.84
Sulfuricurvum	-79.23			5.20
Sulfuriflexus	-83.49			5.43
Sulfurifustis	-84.87			5.52
Sulfurihydrogenibium	-86.78			5.68
Sulfurimonas	-50.93			3.32
Sulfuritalea	-60.67		1.05	4.00
Sulfuritortus	-29.31			1.85
Sulfurivermis	-82.15			5.38
Sulfurospirillum	-81.00	1.07	1.99	5.41
Sulfurovum	-97.87			6.41
Sutterella	-29.54			
Swingsia	-61.40	0.79	1.44	4.10
Symbiobacterium	-81.43			5.33
Synechococcus	-43.54	1.35	2.05	2.99
Syntrophobotulus	-47.49			3.08
Syntrophomonas	-31.51			2.02
Tabrizicola	-85.61			5.57
Tamlana	-84.43			5.53
Tannerella	-108.13		1.34	7.16
Tardiphaga	-84.43			5.53
Tateyamaria	-27.71			
Tatlockia	-104.33		1.17	6.87
Tatumella	-54.68			3.68
Taylorella	-103.20		1.48	6.81
Tenacibaculum	-63.45		1.28	4.22
Tepidanaerobacter	-89.32			5.86
Terasakiella	-99.36			6.54
Teredinibacter	-72.88		1.17	4.78
Terribacillus	-74.51			4.92
Terriglobus	-89.06	0.89	2.02	5.92

Tessaracoccus	-69.54		1.03	4.58
Tetragenococcus	-57.32	0.67	1.47	3.85
Thalassococcus	-101.71			6.71
Thalassolituus	-64.16			4.21
Thalassospira	-103.74		1.27	6.87
Thalassotalea	-83.65		1.49	5.55
Thauera	-104.80		1.35	6.91
Thermaerobacter	-84.96			5.57
Thermanaeromonas	-30.86			1.95
Thermoanaerobacter	-86.08		1.67	5.68
Thermoanaerobacterium	-75.69	0.74	1.44	5.04
Thermobacillus	-29.52			1.87
Thermobaculum				
Thermobifida	-81.26			5.32
Thermoclostridium	-21.88			
Thermocrinis	-73.93			4.87
Thermodesulfatator	-83.86			5.45
Thermodesulfobacterium	-26.61			1.68
Thermodesulfobium	-75.70			4.96
Thermodesulfovibrio	-99.87			6.57
Thermogemmatispora	-80.81			5.29
Thermogutta	-85.28			5.59
Thermomicrobium	-29.92			1.89
Thermomonas	-54.70		1.44	3.62
Thermomonospora	-30.14			
Thermosediminibacter	-84.64			5.50
Thermosipho	-50.49			3.34
Thermosporothrix	-84.91			5.56
Thermosulfidibacter	-30.04			
Thermosynechococcus	-97.11			6.39
Thermotoga	-88.69		1.21	5.83
Thermus	-88.01			5.80
Thioalkalivibrio	-69.78		1.02	4.59
Thiodictyon	-87.34			5.71
Thiohalobacter	-29.41			
Thiomicrorhabdus	-56.61			3.74
Thiomicrospira	-103.23		1.15	6.78
Thiomonas	-64.12			4.23
Thioploca	-106.08			6.99
Tistrella		-0.63		
Tolumonas	-96.42		1.27	6.34
Treponema	-60.80		1.27	4.06
Trichodesmium	-109.63		1.20	7.25
Trichormus	-69.92		1.15	4.64
Trueperella	-26.46			1.69
Tsukamurella	-70.76		1.03	4.67

Tumebacillus	-106.97			7.06
Turicibacter	-52.96			3.49
Turneriella	-84.51			5.50
Undibacterium	-86.31			5.65
Ureaplasma	-86.50		1.25	5.70
Ureibacillus	-57.07			3.75
Waddlia	-79.74			5.22
Vagococcus	-57.56	0.88	1.79	3.86
Variovorax	-93.13		1.53	6.14
Weeksella	-29.36			1.86
Veillonella	-64.65			4.29
Weissella	-41.19		1.25	2.83
Wenyingzhuangia	-75.79		1.14	4.99
Wenzhouxiangella	-84.82			5.52
Verrucomicrobium	-85.47			5.56
Verrucosispora	-84.11			5.47
Vibrio	-47.62	1.07	1.65	3.27
Wigglesworthia	-97.95		1.72	6.46
Winogradskyella	-84.01		1.27	5.57
Virgibacillus	-68.03	0.94	1.63	4.57
Vitreoscilla	-105.95		1.20	6.99
Vogesella	-81.67			5.35
Wolbachia	-46.40	0.69	1.66	3.10
Wolinella	-26.89			
Xanthobacter	-84.55			5.50
Xanthomonas	-45.09	0.89	1.62	3.03
Xenorhabdus	-44.29	0.88	1.48	2.98
Xylanibacterium	-83.74			5.48
Xylella	-111.16		1.24	7.36
Yangia	-88.13			5.80
Yersinia	-51.04	0.94	1.62	3.48
Yoonia	-85.63			5.57
Zhongshania	-31.40			1.98
Zobellella	-85.18			5.57
Zobellia	-84.56			5.50
Zoogloea				
Zunongwangia	-84.95			5.57
Zymobacter	-113.28		1.48	7.46
Zymomonas	-100.64	0.83	1.66	6.67

Table S15. Bacterial families based on metagenomics, shown as for Table S7.

Family	intercept (Estonia)	Finland	Sweden	Read count
Acaryochloridaceae	-92.81			6.04
Acetobacteraceae				
Acholeplasmataceae	-66.15	0.99	1.51	4.44
Acidaminococcaceae	-112.14		1.67	7.39
Acidiferrobacteraceae	-110.55		1.52	7.28
Acidithiobacillaceae	-114.92		1.83	7.60
Acidobacteriaceae	-61.84	0.93	2.10	4.15
Actinomycetaceae	-55.89	0.86	1.41	3.81
Actinopolysporaceae	-92.79			6.05
Aerococcaceae	-72.22	0.84	1.52	4.84
Aeromonadaceae	-45.54		1.24	3.07
Akkermansiaceae	-100.96		1.82	6.68
Alcaligenaceae	-69.99	0.84	1.63	4.70
Alcanivoracaceae	-54.96	0.75	1.79	3.68
Alicyclobacillaceae	-118.24		1.60	7.83
Alteromonadaceae	-51.83	0.94	1.63	3.53
Amoebophilaceae	-113.27		1.92	7.50
Anaerolineaceae	-55.67			3.69
Anaeromyxobacteraceae	-75.73			4.97
Anaeroplasmataceae	-23.24			
Anaplasmataceae	-49.99	0.84	1.58	3.40
Aphanizomenonaceae	-73.64	0.90	1.42	4.93
Aphanothecaceae	-40.97			2.81
Aquificaceae	-90.62		1.32	6.02
Archangiaceae	-94.64	1.06	2.06	6.32
Ardenticatenaceae	-58.13			3.80
Atopobiaceae	-102.76		1.54	6.77
Aurantimonadaceae	-115.01	0.93	2.26	7.64
Azonexaceae	-106.79		1.46	7.03
Bacillaceae	-31.77	1.28	1.57	2.27
Bacteriovoracaceae	-107.92		1.28	7.09
Bacteroidaceae	-69.97	1.06	1.63	4.71
Baekduiaceae	-92.91			6.09
Balneolaceae	-92.00			5.99
Barnesiellaceae	-31.91	-0.84		2.04
Bartonellaceae				
Bdellovibrionaceae	-86.35		1.44	5.76
Beijerinckiaceae	-102.58	0.83	1.90	6.82
Bernardetiaceae	-77.36	0.93	1.87	5.15
Beutenbergiaceae	-41.53			2.70
Bifidobacteriaceae	-38.16	1.32	1.68	2.68
Blattabacteriaceae	-83.01		1.38	5.49

Bogoriellaceae	-106.62		1.29	7.04
Borreliaceae	-56.02	1.08	1.46	3.80
Brachyspiraceae	-77.02		1.41	5.13
Bradymonadaceae	-93.54			6.09
Bradyrhizobiaceae	-82.56	1.23	2.14	5.54
Brevibacteriaceae	-83.11		1.13	5.51
Brucellaceae	-91.02	1.08	1.86	6.07
Budviciaceae	-56.69		1.38	3.78
Burkholderiaceae	-30.53	1.28	1.52	2.19
Caedimonadaceae	-98.34		1.13	6.48
Campylobacteraceae				
Candidatus Actinomarinaceae	-93.85			6.11
Candidatus Babeliaceae	-86.69			5.68
Candidatus Brocadiaceae	-85.69			5.61
Candidatus Deianiraeaceae	-92.30			6.01
Candidatus Desulfofervidaceae	-84.97			5.57
Candidatus Midichloriaceae	-91.66			5.97
Candidatus Nanopelagicaceae	-95.04			6.27
Candidatus Paracaedibacteraceae	-92.92			6.05
Candidatus Sumerlaeaceae	-31.54	-0.83		2.01
Cardiobacteriaceae	-105.43			6.91
Carnobacteriaceae				
Catenulisporaceae	-107.33		1.28	7.05
Caulobacteraceae	-62.30		1.87	4.14
Cellulomonadaceae	-112.38		1.91	7.45
Cellvibrionaceae	-86.11		1.52	5.71
Chamaesiphonaceae	-109.04		1.31	7.17
Chelatococcaceae	-101.20			6.66
Chitinophagaceae	-70.65	1.06	1.64	4.76
Chlamydiaceae	-73.48	0.81	1.55	4.92
Chlorobiaceae	-73.98		1.20	4.94
Chloroflexaceae	-93.86			6.15
Christensenellaceae	-91.52			6.02
Chromatiaceae	-83.41		1.88	5.57
Chromobacteriaceae	-71.65	0.98	1.66	4.82
Chroococcaceae	-114.80	0.94	2.28	7.61
Clostridiaceae	-58.73	1.32	1.55	4.04
Clostridiales Family XIII Incertae S	-59.56			3.94
Clostridiales Family XVI Incertae S	-92.68			6.04
Clostridiales Family XVII Incertae	-77.15			5.09
Cohaesibacteraceae	-105.55		1.40	6.97
Coleofasciculaceae	-93.05			6.06
Colwelliaceae	-81.07	0.86	1.83	5.43
Comamonadaceae	-83.52		1.09	5.54
Conexibacteraceae	-92.87			6.05
Coprothermobacteraceae	-86.35			5.66

Coriobacteriaceae	-92.47			6.02
Corynebacteriaceae	-70.49	1.05	1.81	4.74
Coxiellaceae	-73.27		1.55	4.85
Crocinitomicaceae	-96.17		1.38	6.35
Cryomorphaceae	-92.15			6.00
Cyanobacteriaceae	-82.85		1.12	5.48
Cyanothecaceae	-105.83			6.94
Cyclobacteriaceae	-49.71	0.70	1.61	3.36
Cytophagaceae	-81.92	1.63	2.00	5.53
Deferribacteraceae	-78.71		1.23	5.23
Dehalococcoidaceae	-77.17			5.06
Deinococcaceae	-57.46		0.98	3.90
Dermabacteraceae	-92.26	0.77	1.72	6.12
Dermacoccaceae	-100.35		1.46	6.64
Dermocarpellaceae	-78.82			5.21
Desulfobacteraceae	-101.34	0.93	1.95	6.75
Desulfobulbaceae	-93.09		1.26	6.18
Desulfomicrobiaceae	-57.37			3.79
Desulfovibrionaceae	-70.97	0.98	1.48	4.77
Desulfurellaceae	-93.40			6.08
Desulfurobacteriaceae	-54.18			3.56
Desulfuromonadaceae	-87.09	0.81	1.82	5.80
Dictyoglomaceae	-69.15			4.55
Dietziaceae	-95.48	0.90	1.97	6.36
Dysgonamonadaceae	-83.19			5.46
Ectothiorhodospiraceae	-84.27	0.93	1.89	5.61
Eggerthellaceae	-112.72		1.81	7.46
Egibacteraceae	-32.22	-0.82		2.06
Egicoccaceae	-94.40			6.15
Elusimicrobiaceae	-26.83			1.73
Emcibacteraceae	-81.45		1.03	5.37
Endomicrobiaceae	-102.84		1.51	6.79
Endozoicomonadaceae	-84.56			5.56
Enterobacteriaceae				
Enterococcaceae				
Entomoplasmataceae	-56.92	0.97	1.46	3.88
Erwiniaceae				
Erysipelotrichaceae	-66.09		1.30	4.41
Erythrobacteraceae	-88.95	1.22	2.08	5.96
Eubacteriaceae	-76.63	0.73	1.53	5.11
Euzebyaceae	-92.97			6.06
Ferrimonadaceae				
Fervidobacteriaceae	-72.39	0.68	1.29	4.84
Fibrobacteraceae	-24.58			1.56
Fimbriimonadaceae	-80.39			5.26
Flammeovirgaceae	-64.82		1.20	4.32

Flavobacteriaceae	-93.74	1.01	1.90	6.26
Francisellaceae	-69.59	1.17	1.62	4.69
Frankiaceae	-19.58			1.38
Fusobacteriaceae	-51.19	0.83	1.27	3.50
Gallionellaceae	-91.38		1.46	6.07
Gemmataceae	-101.22		1.36	6.69
Gemmatimonadaceae	-106.37		1.29	7.02
Geobacteraceae	-81.69	1.01	1.69	5.48
Geodermatophilaceae	-99.67			6.55
Gloeobacteraceae	-87.38		1.40	5.77
Glycomycetaceae	-90.07			5.93
Gomontiellaceae	-86.33			5.66
Gordoniaceae	-95.00		1.50	6.29
Gottschalkiaceae	-78.40		1.42	5.19
Granulosicoccaceae	-106.90			7.05
Hafniaceae	-45.82	0.93	1.37	3.16
Hahellaceae	-71.64			4.76
Halanaerobiaceae	-95.02		1.31	6.31
Halieaceae	-86.71		1.14	5.70
Haliscomenobacteraceae	-85.52		1.12	5.62
Halobacteriovoraceae	-96.09		1.36	6.34
Halobacteroidaceae	-55.63			3.68
Halomonadaceae	-71.89	1.05	1.66	4.86
Halothiobacillaceae	-92.82			6.08
Hapalosiphonaceae	-105.55		1.80	6.99
Helicobacteraceae	-64.50	1.15	1.52	4.38
Herpetosiphonaceae	-38.37			2.55
Holosporaceae	-75.05		1.03	4.95
Hungateiclostridiaceae	-74.33	1.21	1.90	5.01
Hydrogenophilaceae	-29.41			1.88
Hydrogenothermaceae	-93.75			6.14
Hyellaceae	-93.57			6.13
Hymenobacteraceae	-76.51	1.01	1.85	5.12
Hyphomicrobiaceae	-73.53	0.88	1.66	4.92
Hyphomonadaceae	-71.30		1.21	4.74
Idiomarinaceae	-83.29		1.35	5.53
Ignavibacteriaceae	-103.63		1.42	6.82
Immundisolibacteraceae	-30.04			1.92
Intrasporangiaceae	-88.89		1.46	5.90
Isosphaeraceae	-58.76	0.75	1.35	3.97
Jiangellaceae	-62.77			4.17
Jonesiaceae	-91.43			5.96
Kangiellaceae	-77.47		1.29	5.13
Kineosporiaceae	-103.48			6.78
Kofleriaceae	-86.17			5.65
Kosmotogaceae	-86.94			5.74

Labilitrichaceae	-104.35			6.89
Lachnospiraceae	-64.95	1.15	1.62	4.40
Lactobacillaceae				
Legionellaceae	-70.54	1.40	1.74	4.79
Leptolyngbyaceae	-106.02		1.41	7.00
Leptospiraceae	-57.64	0.69	1.10	3.91
Leptotrichiaceae	-62.46	0.92	1.54	4.22
Leuconostocaceae				
Listeriaceae	-65.17	1.06	1.78	4.41
Magnetococcaceae	-85.51			5.60
Marinifilaceae	-72.95		1.19	4.83
Marinilabiliaceae	-76.36		1.01	5.06
Mariprofundaceae	-39.16			2.62
Melioribacteraceae	-32.17	-0.84		2.05
Methylacidiphilaceae	-99.26			6.54
Methylobacteriaceae	-86.36		1.68	5.76
Methylococcaceae	-75.73		1.51	5.04
Methylocystaceae	-88.69		1.41	5.86
Methylophilaceae	-66.32	1.00	1.24	4.47
Microbacteriaceae	-54.40		1.52	3.63
Microbulbiferaceae	-75.72	0.70	1.49	5.05
Micrococcaceae	-119.90	0.94	2.24	7.96
Microcoleaceae	-76.21	0.89	1.60	5.11
Microcystaceae	-64.44		0.97	4.29
Micromonosporaceae	-72.03	1.09	1.77	4.83
Moraxellaceae				
Morganellaceae				
Moritellaceae	-82.44		1.42	5.48
Muribaculaceae	-75.36		1.06	5.00
Mycobacteriaceae	-83.32	1.54	2.31	5.63
Mycoplasmataceae				
Myxococcaceae	-87.77		1.51	5.83
Nakamurellaceae	-105.78			6.97
Natranaerobiaceae	-93.16			6.07
Nautiliaceae	-76.08		1.32	5.05
Neisseriaceae	-59.72	1.25	1.88	4.07
Nitrosomonadaceae	-86.42	0.82	1.98	5.74
Nitrospiraceae	-60.32		0.90	4.02
Nocardiaceae	-75.20		1.27	5.01
Nocardioidaceae	-30.31	0.91	1.33	2.13
Nocardiopsaceae	-103.24		1.95	6.86
Nostocaceae	-41.46	0.84	1.13	2.85
Oceanospirillaceae	-77.34	0.96	1.61	5.19
Odoribacteraceae	-104.12		1.39	6.88
Oleiphilaceae	-67.86			4.47
Opitutaceae	-122.50	0.97	2.29	8.14

Orbaceae	-45.90	0.91	1.37	3.16
Oscillatoriaceae	-86.21	0.89	1.66	5.75
Oscillospiraceae	-108.34		1.30	7.12
Oxalobacteraceae	-39.95			2.75
Paenibacillaceae				
Paludibacteraceae	-74.33			4.86
Parachlamydiaceae	-63.88		0.93	4.27
Pasteurellaceae	-45.47	1.04	1.41	3.14
Pectobacteriaceae				
Pelagibacteraceae	-65.36		0.94	4.37
Peptococcaceae	-44.42	0.80	1.17	3.04
Peptoniphilaceae	-65.49			4.37
Peptostreptococcaceae	-79.22	1.48	1.90	5.38
Petrotogaceae	-53.93			3.57
Phyllobacteriaceae	-78.20	1.36	1.81	5.28
Piscirickettsiaceae	-67.11	0.78	1.18	4.50
Planctomycetaceae	-80.56	1.24	1.99	5.42
Planococcaceae	-67.41	1.41	1.92	4.58
Polyangiaceae	-70.03		1.18	4.66
Porphyromonadaceae	-114.64	0.84	2.02	7.61
Prevotellaceae	-83.10	1.15	1.98	5.57
Prochloraceae	-72.94	0.82	1.42	4.91
Prolixibacteraceae	-93.06			6.06
Promicromonosporaceae	-39.01			2.58
Propionibacteriaceae	-92.55			6.03
Pseudanabaenaceae	-106.99		1.45	7.04
Pseudoalteromonadaceae	-74.11	1.42	2.05	5.02
Pseudomonadaceae				
Pseudonocardiaceae	-47.33	0.95	1.41	3.26
Psychromonadaceae	-84.08		1.26	5.57
Puniceicoccaceae	-96.42			6.35
Rhizobiaceae	-37.95	0.87		2.65
Rhodanobacteraceae	-47.60		1.26	3.18
Rhodobacteraceae	-72.21	0.97	1.66	4.85
Rhodobiaceae	-98.96		1.56	6.54
Rhodocyclaceae	-107.51		1.65	7.13
Rhodospirillaceae				
Rhodothermaceae	-67.60		1.18	4.49
Rickettsiaceae				
Rikenellaceae	-87.61	0.89	1.80	5.84
Rivulariaceae	-56.52	1.19	1.28	3.87
Roseiflexaceae	-89.81			5.90
Ruaniaceae	-92.79			6.04
Rubrobacteraceae	-92.59			6.06
Ruminococcaceae	-82.96	1.36	1.89	5.59
Saccharospirillaceae	-23.44			1.66

Salinisphaeraceae	-105.63			6.92
Salinivirgaceae	-85.23			5.58
Sandaracinaceae	-85.32			5.59
Sanguibacteraceae	-104.75			6.86
Saprospiraceae				
Scytonemataceae	-96.28		1.22	6.38
Sedimentisphaeraceae	-105.13			6.89
Segniliparaceae	-32.12	-0.85		2.05
Selenomonadaceae	-72.95		1.10	4.86
Shewanellaceae	-55.02	1.17	1.76	3.75
Silvanigrellaceae	-114.47		1.79	7.59
Sinobacteraceae	-83.50			5.49
Solibacteraceae	-69.98			4.62
Sphaerobacteraceae	-92.93			6.05
Sphingobacteriaceae	-63.93	0.79	1.41	4.31
Sphingomonadaceae	-84.36	1.32	1.92	5.68
Spirochaetaceae	-57.24	0.77	1.15	3.86
Spiroplasmataceae				
Spongiibacteraceae	-116.83		1.86	7.73
Sporolactobacillaceae	-103.66		1.40	6.82
Sporomusaceae	-79.89	0.98	1.64	5.36
Staphylococcaceae	-66.06	1.01	1.59	4.46
Sterolibacteriaceae	-75.05		1.52	4.98
Streptococcaceae				
Streptomycetaceae				
Streptosporangiaceae	-85.89		1.15	5.70
Sutterellaceae	-32.08	-0.84		2.05
Symbiobacteriaceae	-83.73			5.49
Synechococcaceae	-53.01	1.54	2.12	3.65
Synergistaceae	-100.55			6.61
Syntrophaceae	-107.68		1.30	7.08
Syntrophobacteraceae	-85.94			5.63
Syntrophomonadaceae	-111.87		1.34	7.35
Tannerellaceae	-93.07	0.85	2.08	6.20
Thermaceae	-99.19		1.44	6.56
Thermoactinomycetaceae	-116.98		1.75	7.71
Thermoanaerobacteraceae	-66.78		1.20	4.47
Thermoanaerobacterales Family III Incertae Sedis				
Thermoanaerobacterales Family IV	-92.35			6.02
Thermodesulfobacteriaceae	-103.91		1.41	6.84
Thermodesulfobiaceae	-78.32			5.15
Thermogemmatisporaceae	-83.60			5.48
Thermomicrobiaceae	-31.76	-0.84		2.03
Thermomonosporaceae	-22.71			1.60
Thermosporotrichaceae	-86.63			5.67
Thermotogaceae	-111.15		2.01	7.37

Thioalkalispiraceae	-84.23			5.52
Thiobacillaceae	-85.67			5.61
Thiotrichaceae	-72.16		0.98	4.78
Tissierellaceae	-107.16			7.06
Tsukamurellaceae	-74.55		1.00	4.93
Waddliaceae	-83.19			5.46
Vallitaleaceae	-92.72			6.07
Veillonellaceae	-18.13			1.28
Wenzhouxiangellaceae	-91.85			5.98
Verrucomicrobiaceae	-92.05			5.99
Vibrionaceae	-53.06	1.20	1.65	3.64
Vicinamibacteraceae	-100.97			6.64
Xanthobacteraceae	-64.38		1.26	4.31
Xanthomonadaceae				
Yersiniaceae				
Zoogloeaceae	-57.70	1.08	2.01	3.89

Table S16. Fungal genera based on metabarcoding, shown as for Table S7.

Genus	intercept (Estonia)	Finland	Sweden	Read count
Alatospora				
Alternaria				
Amphiporthe				
Antrodia				
Ascobolus				
Ascosphaera				
Aspergillus				
Aureobasidium			1.25	
Batcheloromyces				
Blumeria			1.11	
Botrytis			0.96	
Candida	-11.71		1.23	1.06
Capnobotryella				
Capronia				
Celosporium			1.13	
Ceramothyrium				
Chaetomium				
Cistella				
Citeromyces				
Claviceps				
Clavispora				
Colpoma				
Coniothyrium				
Coprinopsis				
Coprinus				
Cryptococcus				
Cryptodiscus				
Cystofilobasidium				
Davidiella			1.73	
Debaryomyces				
Devriesia				
Dinemasporium				
Diplodia				
Dwayaangam				
Elsinoe				
Embellisia				
Emmonsia				
Endoconidioma			1.28	
Engyodontium				
Epichloe				
Eremascus				
Erysiphe			1.16	

Eucasphaeria		
Exobasidium		
Exophiala		
Fusarium		
Fusicladium		
Golovinomyces		
Gymnoascus		
Gyromitra		
Hanseniaspora		
Hypogymnia		
Knufia		
Kodamaea	-1.05	
Lecanicillium		
Lecidella		
Lecythophora		
Leucosporidiella		
Lophodermium		
Lycoperdon		
Melampsora	-1.05	
Melampsoridium	-1.12	
Metschnikowia	0.94	1.27
Microdochium		
Minimedusa		
Monilinia		
Mortierella		
Mucor		
Mycocentrospora		
Mycosphaerella		
Neoerysiphe		
Neostagonospora		
Ochroconis		
Oculimacula		
Oidiodendron		
Penicillium		
Phacidium		
Phaeococcomyces		1.05
Phaeomoniella		
Phaeotheca		
Phialemonium		
Phlebiopsis		
Piedraia		
Pleospora		
Podosphaera		0.88
Podospora		
Powellomyces		
Preussia		

Pringsheimia		
Pseudogymnoascus		
Pseudoplectania		
Puccinia		
Pucciniastrum		
Radulidium		
Ramularia		
Resinicium		
Rhizophydium	-1.07	
Rhizopus		
Rhizosphaera		1.06
Rhodotorula		
Saccharomyces		
Sawadaea		
Schizophyllum		
Sclerotinia		
Sporobolomyces		
Starmerella		
Thanatephorus		
Thekopsora		
Tilletiopsis		
Tremella		
Truncatella		
Tumularia		
Urocystis		
Wallemia		
Vuilleminia		
Xylodon		
Yarrowia		
Zygosaccharomyces		

Table S17. Fungal families based on metabarcoding, shown as for Table S7.

Family	intercept (Estonia)	Finland	Sweden	Read count
Agaricaceae				
Ajellomycetaceae				
Amanitaceae				
Amphisphaeriaceae				
Ascobolaceae				
Ascosphaeraceae				
Boletaceae				
Botryosphaeriaceae				
Cephalothecaceae				
Ceratobasidiaceae				
Chaetomiaceae		-1.05		
Chaetothyriaceae				
Clavicipitaceae				
Coniochaetaceae				
Cordycipitaceae				
Corticiaceae				
Cortinariaceae				
Cystofilobasidiaceae				
Davidiellaceae			1.97	
Debaryomycetaceae				
Dipodascaceae		-0.97		
Discinaceae				
Dothideaceae			1.31	
Dothioraceae			1.68	
Elsinoaceae				
Eremascaceae				
Erysiphaceae			1.28	
Exobasidiaceae				
Filobasidiaceae		-0.89		
Fomitopsidaceae				
Glomeraceae		-1.06		
Gnomoniaceae				
Gymnoascaceae				
Helotiaceae				
Helvellaceae		-1.03		
Herpotrichiellaceae			1.21	
Hyaloscyphaceae				
Hymenochaetaceae				
Lasiosphaeriaceae				
Lecanoraceae				
Leotiaceae				
Leptosphaeriaceae				

Leucosporidiaceae		
Melampsoraceae	-1.05	
Metschnikowiaceae	0.91	1.45
Microascaceae		
Microbotryaceae		
Mortierellaceae		
Mucoraceae		
Mycosphaerellaceae		
Myxotrichaceae		
Nectriaceae		
Ophiocordycipitaceae		
Orbiliaceae		
Parmeliaceae		
Pezizaceae		
Phacidiaceae		
Phaeosphaeriaceae		
Phanerochaetaceae		
Piedraiaceae		
Pleosporaceae		
Psathyrellaceae	-1.04	
Pseudeurotiaceae		
Pucciniaceae		
Pucciniastraceae	-1.04	
Rhizophydiaceae	-1.02	
Rhizopodaceae	-1.03	
Rhytismataceae		
Russulaceae		
Saccharomycetaceae		
Saccharomycodaceae		
Sarcosomataceae		
Schizophyllaceae		
Schizoporaceae		
Sclerotiniaceae		1.28
Sebacinaceae		
Spizellomycetaceae		
Sporormiaceae		
Stictidaceae		
Synchytriaceae		
Teloschistaceae		
Teratosphaeriaceae		
Thelephoraceae		
Trichocomaceae		
Urocystidaceae		
Wallemiaceae		
Valsaceae		
Venturiaceae 1		1.23

Table S18. Fungal genera based on metagenomics, shown as for Table S7.

Genus	intercept (Estonia)	Finland	Sweden	Read count
Acaromyces	-26.64			1.83
Agaricus	-77.41	0.89	1.57	5.19
Alternaria	-47.10	1.16	1.05	3.27
Ambrosiozyma		-0.85		
Amorphotheca	-69.57			4.60
Annulohypoxylon				
Anthracocystis	-89.54		1.02	5.91
Apiotrichum	-65.59			4.34
Arthrobotrys	-46.42			3.12
Ascoidea	-26.12			1.78
Ascosphaera	-39.93			2.66
Aspergillus	-45.23	1.27	1.30	3.12
Aureobasidium	-27.72	1.18	1.12	1.99
Babjeviella	-80.34			5.31
Batrachochytrium	-50.46			3.32
Baudoinia	-60.22			3.97
Beauveria	-60.82			4.03
Berkeleyomyces				
Bettsia				
Bipolaris	-45.08		1.22	3.05
Blastomyces	-79.81		0.87	5.30
Blumeria	-54.41	1.02	1.27	3.71
Botrytis	-31.57			2.15
Brigantiaea		-0.85		
Byssochlamys	-65.00			4.30
Cairneyella				
Candida				
Candida.1	-67.86			4.52
Capronia	-86.49	1.12	1.62	5.81
Ceraceosorus	-80.69			5.29
Cercospora	-41.76			2.83
Chaetomium	-23.70			1.64
Chionosphaera				
Cladonia	-67.32			4.38
Cladophialophora	-85.29	0.89	1.59	5.72
Cladosporium	-25.50			1.61
Claviceps				
Clavispora	-41.15	0.93	1.18	2.82
Clonostachys				
Coccidioides	-56.13		0.89	3.79
Colletotrichum	-61.40	1.34	1.28	4.16
Coniophora	-83.77		1.21	5.56

Coniosporium	-71.83			4.72
Coprinopsis	-89.19		1.00	5.91
Cordyceps	-37.69		0.72	2.52
Cryptococcus				
Cutaneotrichosporon	-56.94			3.76
Cyberlindnera	-61.20		0.76	4.11
Cyniclomyces				
Cyphellophora	-68.90			4.55
Debaryomyces				
Dichomitus	-64.74			4.30
Diplodia	-84.60		1.14	5.61
Encephalitozoon	-75.90			5.00
Endocarpon	-102.21		1.09	6.77
Epichloe	-71.54		0.91	4.78
Eremothecium				
Exophiala	-42.84	0.97		2.96
Exserohilum				1.19
Fibroporia	-77.15			5.09
Flammulina		-0.84		
Fomitiporia	-95.86		1.01	6.35
Fonsecaea	-68.63	0.91	1.13	4.60
Fusarium	-37.83	1.14	1.08	2.64
Gaeumannomyces	-70.44			4.67
Glarea	-50.06			3.33
Gloeophyllum	-62.42			4.12
Grosmannia	-93.61		1.27	6.20
Heterobasidion	-59.94			3.98
Hirsutella	-67.50			4.39
Histoplasma	-69.70			4.58
Holtermannia		-0.86		
Hyaloscypha	-91.53	0.87	1.58	6.13
Hyphopichia				
Jaminaea	-90.15		1.01	5.97
Juglanconis	-66.56			4.33
Kalmanozyma	-71.48			4.68
Kazachstania				
Kluyveromyces				
Kockovaella	-74.89			4.93
Komagataella	-37.99			2.55
Kuraishia	-40.03			2.63
Kwoniella	-67.03	0.77	1.40	4.50
Laccaria	-56.12			3.71
Lachancea				
Leptosphaeria				
Lichtheimia	-50.49		0.99	3.41
Lipomyces		-0.85		

Lobosporangium	-81.21		1.17	5.41
Lodderomyces	-55.15			3.68
Malassezia	-76.40			5.04
Marssonina	-60.55		0.93	4.05
Meira	-70.17			4.66
Melampsora	-62.96			4.24
Melanopsichium	-83.70		1.04	5.53
Metarhizium	-68.37	0.86	1.31	4.59
Metschnikowia	-47.77	0.99	1.17	3.26
Meyerozyma	-54.31			3.62
Microbotryum				
Microsporum	-58.12			3.84
Millerozyma	-50.91			3.39
Mitosporidium				
Mixia	-59.34			3.92
Moesziomyces	-56.78			3.78
Nakaseomyces				
Nakazawaea		-0.84		
Nannizzia	-75.41			4.97
Naumovozyma				
Nematocida	-25.97			1.66
Neurospora	-101.90		1.36	6.78
Nosema	-66.24			4.38
Ogataea	-47.80			3.16
Paracoccidioides	-81.82		1.02	5.41
Paraphaeosphaeria	-74.56	0.85	1.21	4.99
Parastagonospora	-59.39		1.12	4.00
Peltigera				
Penicilliopsis	-48.65			3.22
Penicillium				
Pestalotiopsis	-56.48			3.75
Phaeoacremonium	-68.50			4.52
Phakopsora	-40.37			2.62
Phanerochaete	-75.26			4.96
Phialocephala	-68.96			4.57
Phialophora	-26.32			1.74
Phycomyces	-73.73	0.75	1.03	4.93
Pichia	-56.85		0.80	3.84
Pneumocystis	-86.18		1.04	5.74
Pochonia				
Podospora	-68.02	1.03	1.25	4.59
Postia	-79.19			5.25
Proxipyricularia				
Pseudocercospora	-82.34		0.92	5.45
Pseudogymnoascus	-75.11	1.55	1.59	5.07
Pseudomicrostroma	-42.33			2.78

Pseudozyma	-84.50			5.60
Puccinia	-66.27		0.83	4.43
Punctularia	-87.13		0.98	5.77
Purpureocillium	-72.42		1.10	4.83
Pyrenophora	-84.90		0.94	5.63
Pyricularia	-42.88	0.87	1.14	2.92
Pyronema				
Ramularia	-91.42		1.21	6.08
Rasamsonia	-98.42		1.35	6.54
Rhinocladiella	-66.09			4.39
Rhizophagus				
Rhizopus	-67.58		0.86	4.51
Rhodotorula	-68.32		0.86	4.56
Russula			-0.88	
Saccharomyces				
Saccharomycopsis				
Saitoella	-68.62			4.55
Sarocladium				
Scedosporium	-94.82		1.10	6.29
Scheffersomyces	-32.83			2.18
Schizophyllum	-75.40			4.97
Schizosaccharomyces	-58.16	0.82	1.31	3.95
Schwanniomyces		-0.83		
Sclerotinia	-27.81	1.16	1.09	1.99
Serpula	-70.00			4.61
Sodiomyces	-81.29		0.99	5.40
Sordaria	-58.56			3.87
Sparassis	-91.49		0.93	6.06
Spathaspora	-60.26			4.01
Sphaerulina	-51.40			3.43
Spizellomyces	-91.24			6.03
Sporisorium				
Sporothrix	-32.34			2.14
Stereum	-83.82			5.55
Strobilomyces				
Sugiyamaella	-50.48			3.35
Suhomyces	-54.48			3.64
Talaromyces	-43.06	1.24	1.25	2.98
Tetrapisispora				
Thermothelomyces	-80.24		1.14	5.35
Thermothielavioides	-74.75		0.95	4.99
Tilletiaria	-72.04		0.94	4.80
Tilletiopsis	-75.75			5.00
Torulaspora				
Trametes	-59.41		0.91	3.97
Tremella	-70.21			4.63

Trichoderma	-40.74	0.87	0.91	2.79
Trichophyton	-74.40		1.14	4.97
Trichosporon	-74.18			4.89
Tuber	-68.63			4.49
Umbelopsis		-0.85		
Uncinocarpus	-91.95			6.08
Ustilago	-71.24		1.07	4.78
Wallemia		-0.74		
Vanderwaltozyma				
Venturia	-83.95	1.03	1.67	5.64
Verruconis	-86.94			5.73
Verticillium	-83.38	1.15	1.47	5.60
Wickerhamia	-20.05			
Wickerhamiella	-81.80			5.38
Wickerhamomyces				
Xanthophyllomyces	-72.65		1.11	4.85
Xylona	-77.42			5.13
Yamadazyma	-39.74			2.63
Yarrowia	-72.85			4.83
Zygosaccharomyces				
Zymoseptoria	-41.52		1.20	2.82

Table S19. Fungal families based on metagenomics, shown as for Table S7.

Family	intercept (Estonia)	Finland	Sweden	Read count
Agaricaceae	-81.10	1.00	1.65	5.44
Ajellomycetaceae	-58.86		0.93	3.93
Arthrodermataceae	-59.08	1.14	0.91	3.98
Ascoideaceae	-28.01			1.91
Ascosphaeraceae	-42.05			2.79
Aspergillaceae	-43.08	1.52	1.15	3.00
Bionectriaceae				
Boletaceae				
Bondarzewiaceae	-61.55			4.08
Botryosphaeriaceae	-91.14		1.33	6.06
Brachybasidiaceae	-72.23		0.89	4.79
Brigantiaeaceae				
Ceraceosoraceae	-83.22			5.45
Ceratocystidaceae				
Chaetomiaceae	-32.00	1.13		2.24
Chionosphaeraceae				
Cladoniaceae	-71.63			4.65
Cladosporiaceae	-27.05			1.71
Clavicipitaceae	-32.92	1.67	1.19	2.36
Coniophoraceae	-84.66		1.28	5.62
Cordycipitaceae	-44.95	0.92	1.08	3.02
Cryphonectriaceae	-68.59			4.48
Cryptobasidiaceae	-30.26			2.08
Cryptococcaceae		1.46		
Cuniculitremaceae	-78.62			5.17
Cyphellophoraceae	-68.75			4.54
Dacryobolaceae	-79.14			5.25
Debaryomycetaceae		1.09		
Dermateaceae	-62.57		0.97	4.18
Didymosphaeriaceae	-77.00	0.96	1.27	5.16
Dipodascaceae	-72.69			4.82
Erysiphaceae	-57.74	1.17	1.32	3.93
Gloeophyllaceae	-63.40			4.18
Glomeraceae				
Glomerellaceae	-64.51	1.57	1.33	4.37
Helotiaceae	-55.25	0.74	0.79	3.69
Herpotrichiellaceae	-37.13	1.43		2.61
Hyaloscyphaceae	-92.52	0.94	1.62	6.19
Hymenochaetaceae	-102.79		1.35	6.82
Hypocreaceae	-46.74	1.24	0.98	3.19
Hypoxylaceae				
Juglanconidaceae	-71.47			4.64

Leptosphaeriaceae		1.43		
Lichtheimiaceae	-53.02	0.72	1.02	3.58
Lipomycetaceae				
Magnaporthaceae	-71.41			4.73
Malasseziaceae	-77.12			5.09
Melampsoraceae	-64.74			4.36
Metschnikowiaceae	-47.23	1.58	1.30	3.26
Microascaceae	-94.23		1.13	6.25
Microbotryaceae				
Mixiaceae	-59.31			3.91
Mortierellaceae	-86.49		1.35	5.76
Mrakiaceae	-75.49		1.18	5.04
Mycosphaerellaceae	-37.85	1.44		2.66
Myxotrichaceae	-69.42			4.58
Nectriaceae	-43.25	1.52	1.16	3.01
Nosematidae	-69.50		0.98	4.60
Onygenaceae	-93.83			6.20
Ophiocordycipitaceae	-71.74		1.39	4.80
Ophiostomataceae	-32.56			2.21
Orbiliaceae	-50.44	0.81		3.39
Parmeliaceae	-83.20			5.45
Peltigeraceae				
Phaeosphaeriaceae	-62.55		1.17	4.21
Phaffomycetaceae				
Phakopsoraceae	-40.81			2.65
Phanerochaetaceae	-77.67			5.12
Phycomycetaceae	-74.74	0.86	1.07	4.99
Physalacriaceae				
Pichiaceae				
Plectosphaerellaceae	-74.10	1.52	1.57	4.99
Pleosporaceae	-33.18	1.71	1.23	2.37
Pleurotaceae	-70.85			4.60
Pneumocystidaceae	-87.21		1.09	5.80
Polyporaceae	-56.35	0.95	1.16	3.78
Psathyrellaceae	-92.76		1.11	6.15
Pseudeurotiaceae	-78.36	1.81	1.68	5.29
Pucciniaceae	-67.23			4.49
Punctulariaceae	-88.75		1.06	5.88
Pyriculariaceae	-56.87	1.31	1.41	3.87
Pyronemataceae				
Ramalinaceae				
Rhizopodaceae	-70.29			4.69
Russulaceae			-0.94	
Saccharomycetaceae		1.04		
Saccharomycopsidaceae		0.95		
Saccotheciaceae	-33.50	1.72	1.24	2.40

Sarocladiaceae				
Schizophyllaceae	-78.26			5.16
Schizosaccharomycetaceae	-62.09	0.89	1.33	4.21
Sclerotiniaceae	-33.28	1.72	1.24	2.38
Serpulaceae	-71.81			4.73
Sordariaceae	-77.01		1.22	5.14
Sparassidaceae	-93.80		1.02	6.21
Spizellomycetaceae	-93.67			6.19
Sporidiobolaceae	-77.43		1.06	5.16
Sporocadaceae	-57.80			3.84
Stereaceae	-83.54			5.53
Sympoventuriaceae	-89.94			5.93
Teratosphaeriaceae	-61.21			4.03
Thermoascaceae	-65.13			4.31
Tilletiariaceae	-73.71		0.99	4.91
Togniniaceae	-68.58			4.53
Tremellaceae	-71.64			4.72
Trichocomaceae	-47.92	1.59	1.30	3.31
Tricholomataceae	-67.35			4.46
Trichomonascaceae	-52.48			3.51
Trichosporonaceae	-50.39			3.36
Tuberaceae	-71.73			4.69
Umbelopsidaceae				
Unikaryonidae	-78.93			5.20
Ustilaginaceae		1.41		
Wallemiaceae		-0.77		
Venturiaceae	-88.40	1.15	1.78	5.93
Verrucariaceae	-104.26		1.16	6.90
Xylonaceae	-78.49			5.20
